# Total synthesis of the reported structure of 13a-hydroxytylophorine

**DOI:** 10.1038/s41598-017-17015-8

**Published:** 2017-12-05

**Authors:** Hui Zhang, Gang Li, Bo Su, Meng Deng, Yu-Xiu Liu, Yu-Cheng Gu, Qing-Min Wang

**Affiliations:** 1 State Key Laboratory of Elemento-Organic Chemistry, Research Institute of Elemento-Organic Chemistry, College of Chemistry, Nankai University, Tianjin, 300071 People’s Republic of China; 2 Syngenta, Jealott’s Hill International Research Centre, Bracknell, Berks RG42 6EY UK; 3 Collaborative Innovation Center of Chemical Science and Engineering (Tianjin), Tianjin, 300071 People’s Republic of China

## Abstract

The first total synthesis of the reported structure of 13a-hydroxytylophorine was accomplished. The key step was an unprecedented NaBH_4_-promoted one-pot reductive cyclization cascade that efficiently yielded a hydroxyl azonane intermediate. The indolizidine framework was obtained by means of oxidation and a subsequent unexpected protecting-group migration. This total synthesis revealed that the reported structure of the naturally isolated compound is incorrect.

## Introduction

**Figure 1:**
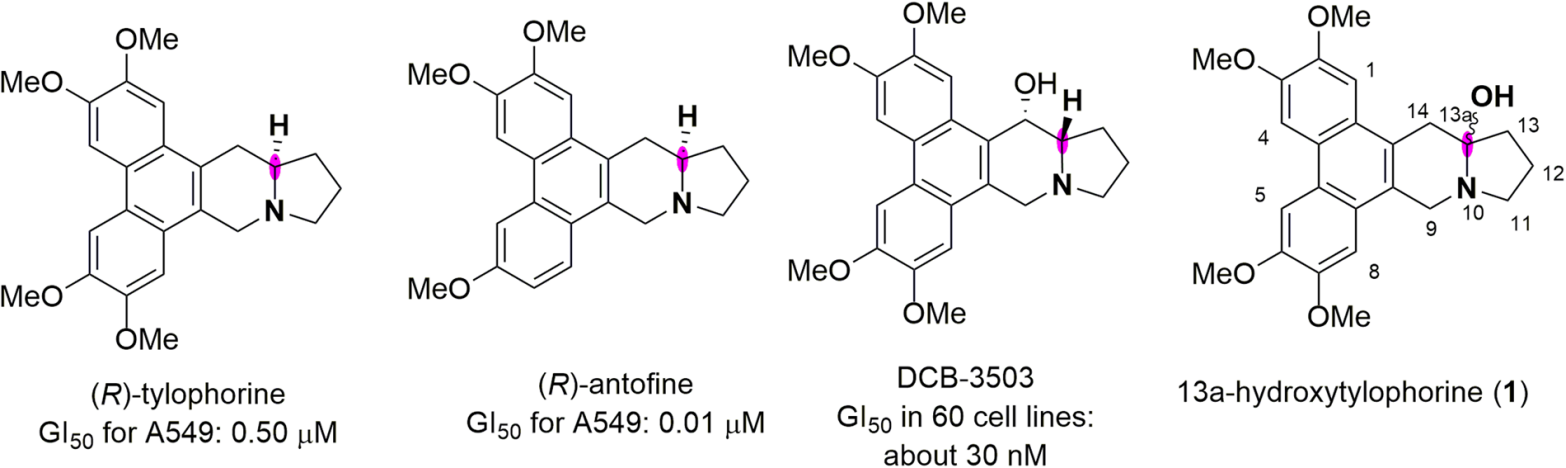
Representative phenanthroindolizidine alkaloids.

Phenanthroindolizidine alkaloids, which are isolated from plants of the *Cynanchum*, *Pergularia*, and *Tylophora* genera, as well as some genera of the *Asclepiadaceae* family^1,2^, are a group of pentacyclic natural products. To date, more than 60 members of this group have been isolated and characterized, and they have been the subject of considerable research attention because they exhibit a variety of bioactivities^3–6^, including anticancer, anti-inflammatory, and antifungal activities. For example, (*R*)-tylophorine and (*R*)-antofine show potent anticancer activity against human lung cancer cells (A549), with 50% growth inhibition concentrations (GI_50_) of 0.50 and 0.01 μM, respectively^7^. In addition, the synthetic analogue DCB-3503, for example, is reported to have an average GI_50_ ofapproximately 30 nM in 60 cell lines^8^ (Fig. 1). Apart from these common C13a-H compounds, a unique compound with a C-13a hydroxyl group, 13a-hydroxytylophorine (**1**), was isolated from *Tylophora hirsuta* by Bhutani *et al*.^9,10^ in 1984.

The 13a-hydroxytylophorine molecule has an aza-hemiacetal motif, and because aza-hemiacetals are usually acid- and base-sensitive, an effective method for construction of this motif constitutes the major challenge in the synthesis of 13a-hydroxytylophorine. Although numerous synthetic approaches to 13a-H phenanthroindolizidine alkaloids have been reported^11–15^, along with a few syntheses of 13a-methyl compounds^16–19^, the total synthesis of 13a-hydroxytylophorine has not yet been achieved. As part of our ongoing research^20–23^ on the synthesis of phenanthroindolizidine alkaloids and systematic evaluation of their biological activities, we herein report the first total synthesis of the reported structure of 13a-hydroxytylophorine.

## Results

**Figure 2:**
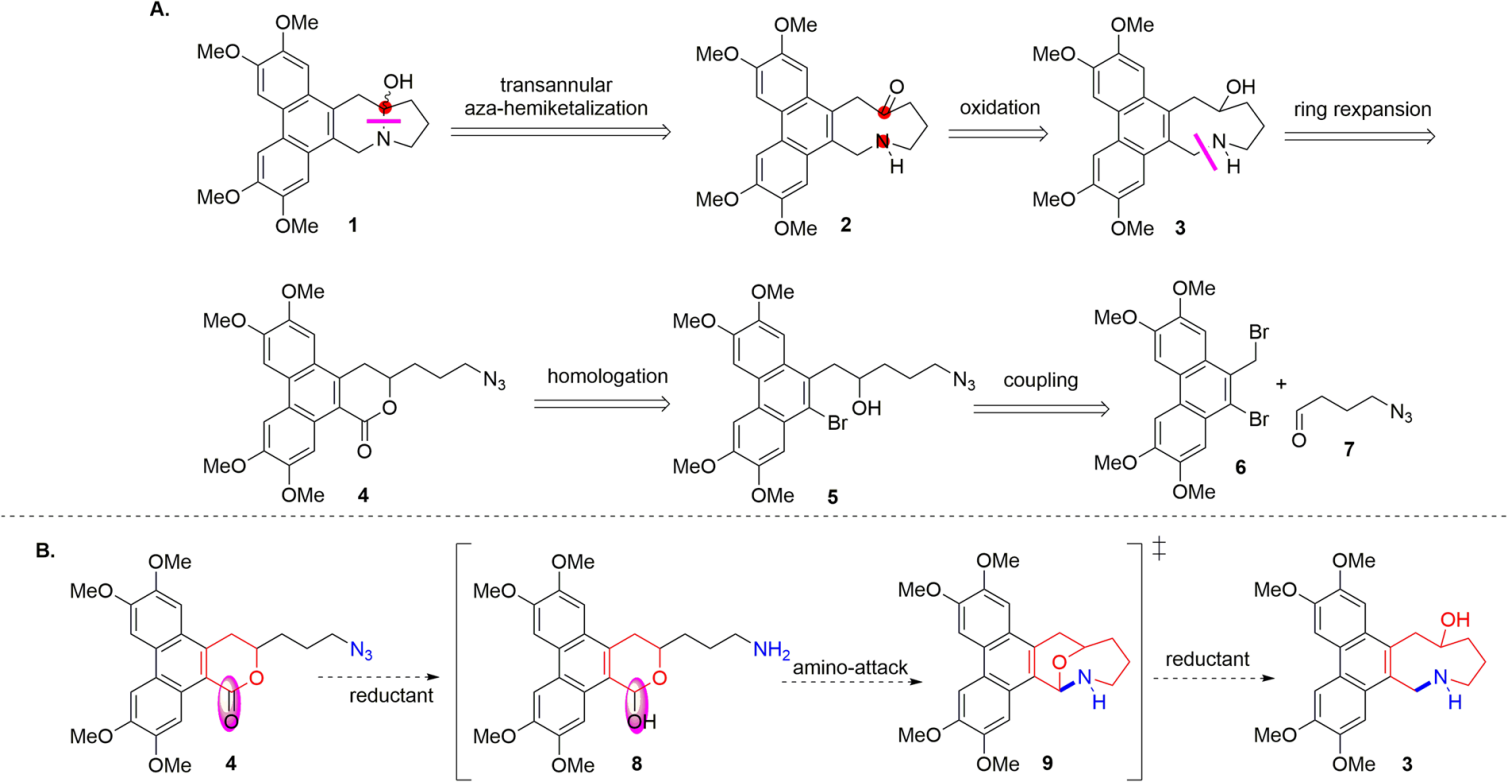
(**A**) Retrosynthetic analysis of 13a-hydroxytylophorine **1** and (**B**) proposed one-pot reductive cyclization cascade.

Our retrosynthetic analysis is presented in Fig. 2A. To circumvent the problem of the lability of the aza-hemiacetal motif, we decided that a novel strategy involving late-stage transannular aza-hemiketalization reaction of amino ketone **2** to form the N–C13a bond would make sense. However, synthesis of the strained phenanthro-annulated nine-membered-ring azonane motif in **2** presented a challenge. Traditional strategies for the preparation of this core architecture involve direct methods such as N-substitution reactions^24^ (e.g., Mitsunobu reaction and alkylation), lactamization^25^, and ring closure metathesis^26^. Indirect methods involving ring expansion^27–29^ via fragmentation of azabicycles offered another option, although syntheses using such methods tend to be complex. We suspected that the rigidity and steric bulk of the phenanthrene ring might render the direct methods unfeasible for our purposes. In addition, because the ring-expansion strategy might be favoured by release of transannular strain, we adopted this indirect strategy.

We expected that amino ketone **2** could be obtained by oxidation of alcohol **3**, which could be prepared from phenanthro-δ-lactone **4** by a ring-expansion reaction. Compound **4** would be prepared by a simple homologation reaction of functionalized precursor **5**, which would in turn besynthesized via a coupling reaction between known dibromo compound^30,31^ **6** and 4-azido-aldehyde **7**. As mentioned above, the ring-expansion reaction is the key step in this strategy. We hoped to prepare key hydroxy-substituted azonane intermediate **3** in one pot by means of a reductive cyclization cascade reaction of lactone **4** (Fig. 2B). Specifically, in the presence of a suitable reductant, lactone **4** would be reduced to lactol^32^ **8**, which would undergo rapid intramolecular attack by the amino group formed *in situ* to generate hemiaminal **9**. Hemiaminal **9** would then be further reduced to required compound **3**. That is, the azonane ring of **3** would be constructed from a six-membered-ring lactone with the necessary oxygen atom in the correct position.

**Figure 3:**
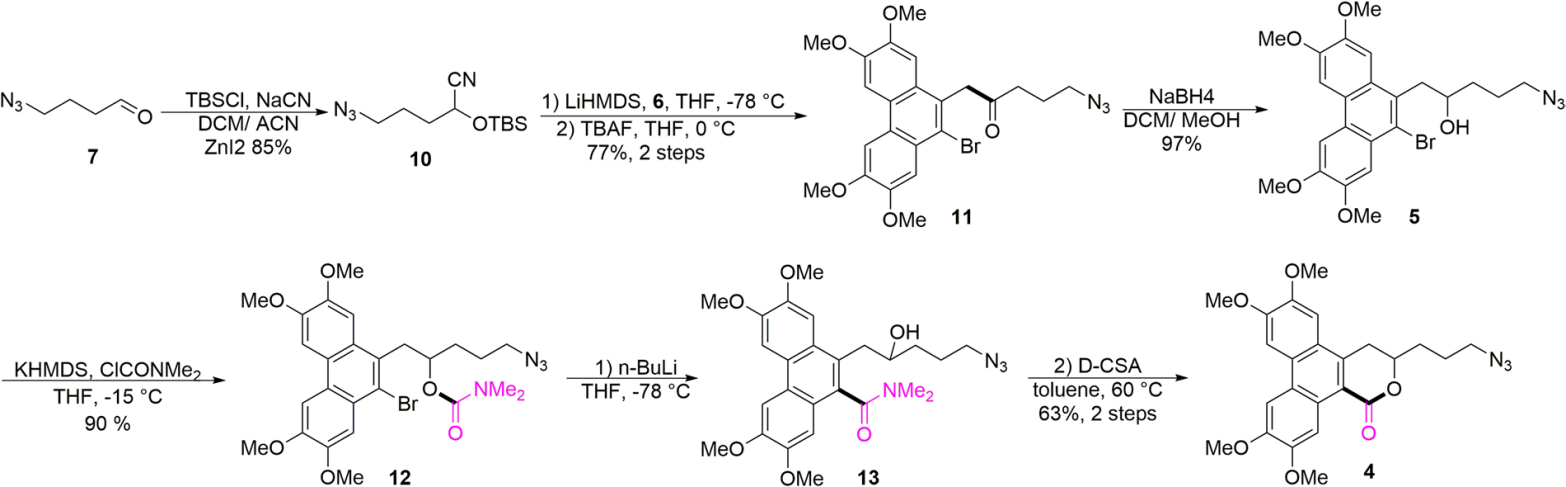
Synthesis of lactone **4**.

We began by constructing the required lactone skeleton (Fig. 3). To make the functionalized precursor of **4**, we used an umpolung strategy. An acyl anion equivalent, namely, *tert*-butyldimethylsilyl-protected cyanohydrin **10**, was prepared in 85% yield via reaction of known 4-azido-aldehyde **7** with NaCN and TBSCl in the presence of a catalytic amount of ZnI_2_. Cyanohydrin **10** was then smoothly coupled with phenanthryl bromide **6** in the presence of LiHMDS as a base. The resulting coupled adduct was treated directly with TBAF to afford ketone **11** in 77% yield over two steps. Subsequent reduction with NaBH_4_ gave alcohol **5** in 97% yield. Next, we needed to install the last one-carbon unit of the target molecule framework. Knowing that lithium-halogen exchange can be used to generate a phenanthryl anionic species, we planned to introduce the necessary carbonyl group via an intramolecular acyl rearrangement from the oxygen atom of the hydroxyl group to the phenanthrene ring. Treatment of alcohol **5** with dimethylcarbamoyl chloride in the presence of KHMDS smoothly gave carbamate **12** in 90% yield. Satisfyingly, reaction of **12** with *n*-BuLi effected the desired acyl rearrangement to afford carbamoyl compound **13**. Subsequent lactonization catalysed by CSA (camphorsulfonic acid) gave phenanthro-δ-lactone **4** in 63% yield over two steps. With **4** in hand, we explored our proposed one-pot reductive cascade sequence.

**Figure 4:**
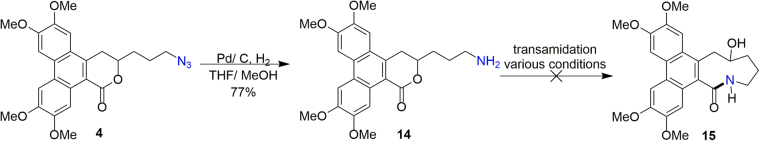
Synthesis of lactone **14** and exploration of transamidation conditions.

Initially, we speculated that when the tethered azido group was reduced to an amino group by a suitable reductant, intramolecular transamidation might occur to produce nine-membered-ring lactam **15**. However, we were concerned that direct transamidation might be unfavourable owing to the transannular strain in the nine-membered ring. In fact, we found that under common azide reduction conditions (10% Pd/C, H_2_), amino lactone **14** was obtained (77% yield) instead of lactam **15** (Fig. 4). Therefore, we explored alternative conditions for the desired intramolecular transamidation of **14**. No reaction occurred under either basic conditions (DBU, TEA, NaOMe, and K_2_CO_3_) or acidic conditions (CSA and HCl). On the basis of these results, we suspected that the lactone centre of **14** was not sufficiently electrophilic to undergo either inter- or intramolecular attack by the amino group. Therefore, we decided to transform the lactone to the corresponding lactol, which would be more susceptible to intramolecular attack. Unlike the transamidation product (lactam **15**), the product of reaction of the lactol, that is, **3**, would not have an acyl group, so further transformation would be unnecessary.

Because DIBAL-H is commonly used to prepare lactols, we used this reagent in our initial attempts to reduce lactone **4**. However, none of the desired product was detected (Table 1, entry 1). We screened several other hydride reductants (NaBH_4_, LiBH_4_, NaBH(OAc)_3_ and NaBH_3_CN) and found that only NaBH_4_^33,34^ in methanol gave any of the desired product, which was obtained in 20% yield along with recovered substrate (entries 2–5). Increasing the reaction temperature to 50 °C increased the yield to 40% (entry 9). Screening of various solvents revealed that methanol was indispensable; ethanol, isopropanol, and THF were not acceptable substitutes (entries 5–8). Finally, we found that reaction in 1:4 (v/v) THF/MeOH at 50 °C afforded the required azonane in 84% yield (entry 10). The reaction was relatively clean, and no intermolecular reaction product was observed. We suggest that the added THF improved the solubility of the substrate, which is only poorly soluble in methanol. We speculated that methanol also enhanced the reductive ability of NaBH_4_ and, moreover, that methanol facilitated the difficult transformation by stabilizing newly formed lactol **8** (see Fig. 2B) and promoting rapid intramolecular attack by the amino to form hemiaminal **9** (which was detected by high-resolution mass spectrometry and ^1^H NMR). Hemiaminal **9** was then reduced to desired product **3**. Detailed information regarding the proposed mechanism is presented in the supplementary materials. To our delight, upstream azide **4** could be transformed to **3** in 74% yield under the same conditions if the amount of reductant was doubled and the reaction time was extended (entry 11). Owing to the tendency of **14** to decompose, the one-pot reduction of **4** was more favourable. In summary, we developed an unprecedented one-pot reductive cyclization cascade to construct the desired strained nine-membered ring from a six-membered-ring lactone under mild conditions with readily available, inexpensive NaBH_4_. The whole procedure was efficient and operationally simple.

**Table 1:** Optimization of one-pot reductive cyclization conditions^a^.


Entry	Reductant	Solvent	Temp	Yield^b^
1^*c*^	DIBAL-H	DCM	−78 °C → rt	ND^d^
2	LiBH_4_	THF	0 °C → rt	NR
3	NaBH(OAc)_3_	DCM	0 °C → rt	NR
4	NaBH_3_CN	DCM/TFA	0 °C → rt	ND
5	NaBH_4_	MeOH	0 °C → rt	20%^e^
6	NaBH_4_	EtOH	0 °C → rt	mix^d,e^
7	NaBH_4_	*i*-PrOH	0 °C → rt	NR
8	NaBH_4_	THF	0 °C → reflux	NR
9	NaBH_4_	MeOH	0 °C → 50 °C	40%^d^
10	NaBH_4_	THF/MeOH^*f*^	0 °C → 50 °C	84%
11 ^*g*^	NaBH_4_	THF/MeOH	0 °C → 50 °C	74%

^a^Reaction conditions: **14** (0.50 mmol, 1 equiv), reductant (5 equiv), solvent (20 mL, 0.025 M), start at 0 °C and then increase to the required temperature, usually < 0.5 h. ^b^Isolated yields are provided. NR, no reaction; ND, not detected. ^c^DIBAL-H (2.5 equiv) was added at −78° C under argon. ^d^A complex mixture was obtained. ^e^Substrate was recovered. ^*f*^The reaction was conducted in 1:4 (v/v) THF/MeOH. ^g^Azide **4** (0.50 mmol, 1 equiv) was used as the substrate, and 10 equiv of reductant was used.

**Figure 5:**
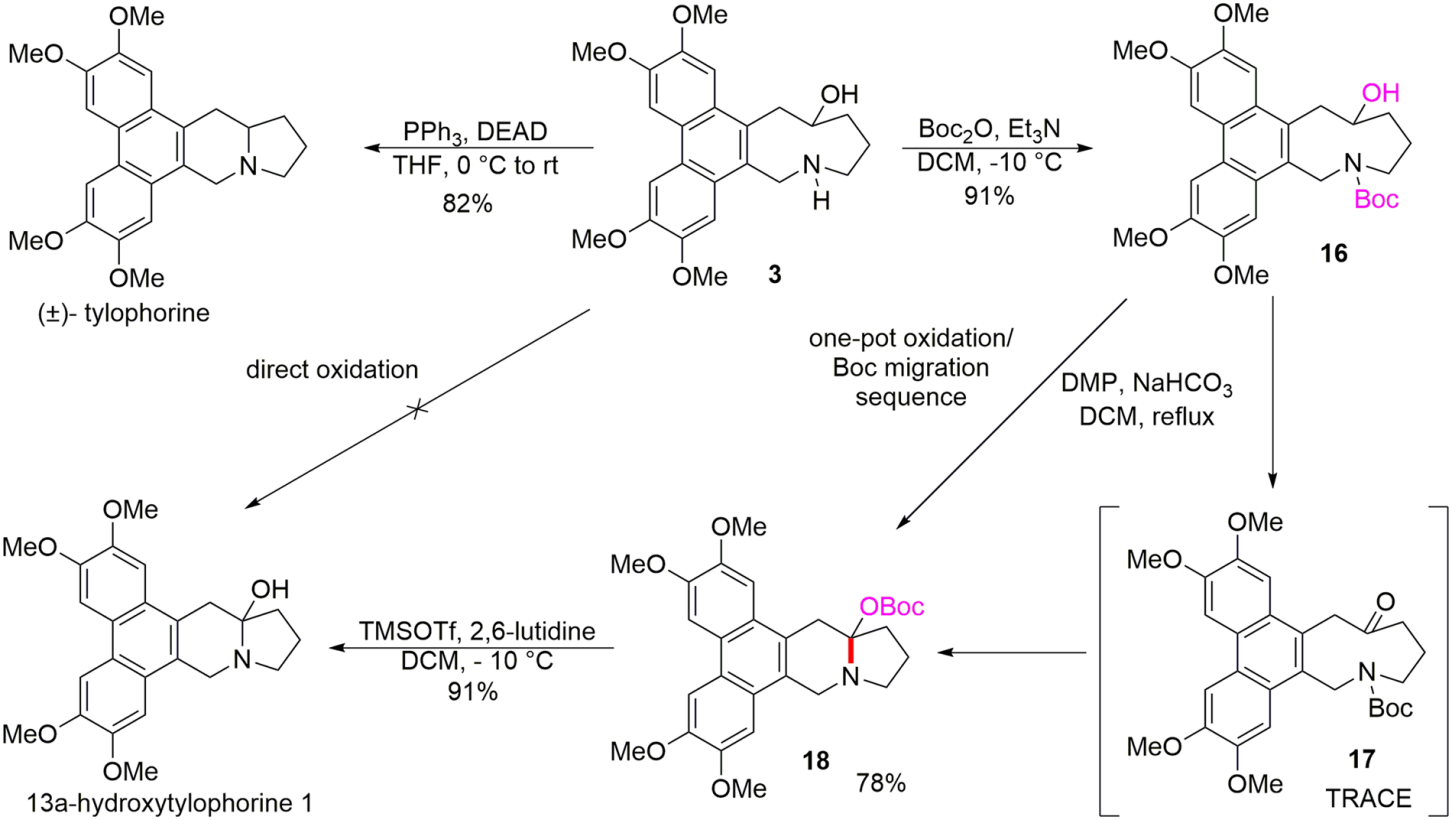
Syntheses of (±)-tylophorine and 13a-hydroxytylophorine (**1**).

With the azonane architecture built, we were able to obtain (±)-tylophorine easily in 82% yield by means of a transannular Mitsunobu reaction in the presence of PPh_3_ and DIAD (Fig. 5). The ^1^H NMR and ^13^C NMR spectra of the synthetic (±)-tylophorine were consistent with those of standard samples prepared previously in our lab^35,36^. Thus, the synthesis described herein confirmed the structure of **3**. To convert **3** to 13a-hydroxytylophorine, we needed to oxidize the hydroxyl group selectively. We envisioned that the formation of a quaternary ammonium salt might prevent undesired oxidation of free amine. We attempted to use Jones reagent to accomplish this transformation, but the reaction yielded a complex mixture. Other oxidative reaction conditions (IBX/HCl and HOAc/PIDA/DCM) also failed to afford the desired alkaloid. Therefore, we protected the free amino group of **3** by treating it with Boc_2_O and trimethylamine, which afforded **16** in 91% yield. When **16** was treated with Dess–Martin periodinane, however, only a trace of desired product **17** was obtained, and the majorproduct was instead 13a-*O*-Boc-tylophorine (**18**) (78% yield; ^1^H NMR, ^13^C NMR, and two-dimensional NMR spectra and HRMS dataare available in the supplementary materials). That is, a one-pot oxidation and unexpected Boc migration took place. We assumed that this transformation proceeded by initial oxidation of the alcohol by DMP to afford expected product **17**. Promoted by the release of transannular strain, **17** underwent rapid migration of the Boc group from the nitrogenatomto the oxygen atom. Steric hindrance between the phenanthrene ring and the bulky *N*-Boc group in**17** may also have contributed to the driving force for this transformation. Compound **17** was actually observed by thin-layer chromatography when the reaction was conducted at −15 °C, but it was soon transformed to compound **18** at room temperature or when in contact with silica gel. Finally, having obtained the indolizidine moiety in this unexpected way, we generated 13a-hydroxytylophorine (**1**) smoothly in 91% yield by removing the protecting group under mild conditions (TMSOTf, 2,6-lutidine).

**Figure 6:**
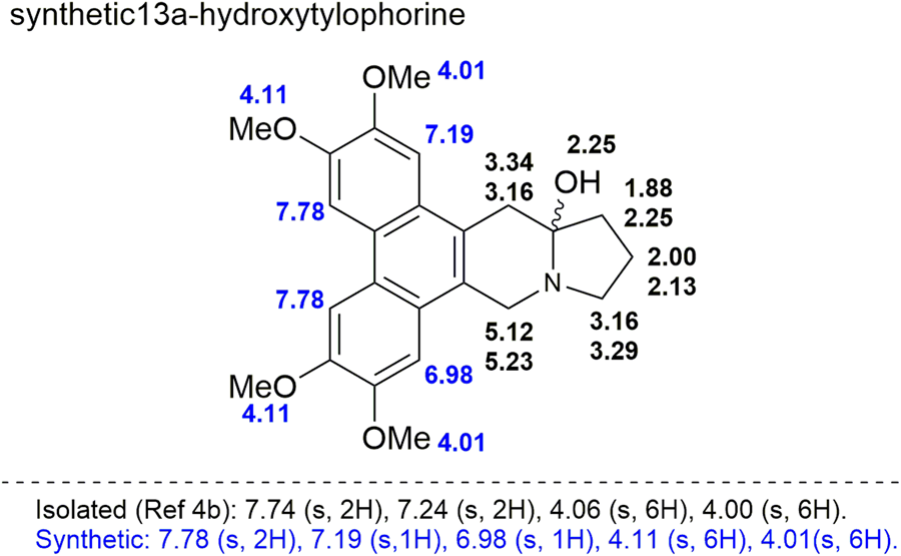
Assignment of chemical shifts of the protons of synthetic 13a-hydroxytylophorine and comparison with the reported chemical shifts for the isolated natural product.

To our disappointment, we found that the ^1^H NMR spectroscopic data for our synthetic 13a-hydroxytylophorine (**1**)differed considerably from the data for the sample isolated by Bhutani *et al*.^10^ Therefore, we conducted a detailed comparison of the chemical shifts of the protons of our synthetic **1** and the chemical shifts reported for the naturally isolated compound (Fig. 6). In particular, we noted that the chemical shifts of the aromatic protons of our synthetic sample differed markedly from those of the isolated compound, and the shifts of the four methoxyl group were slightly different as well.

**Figure 7:**
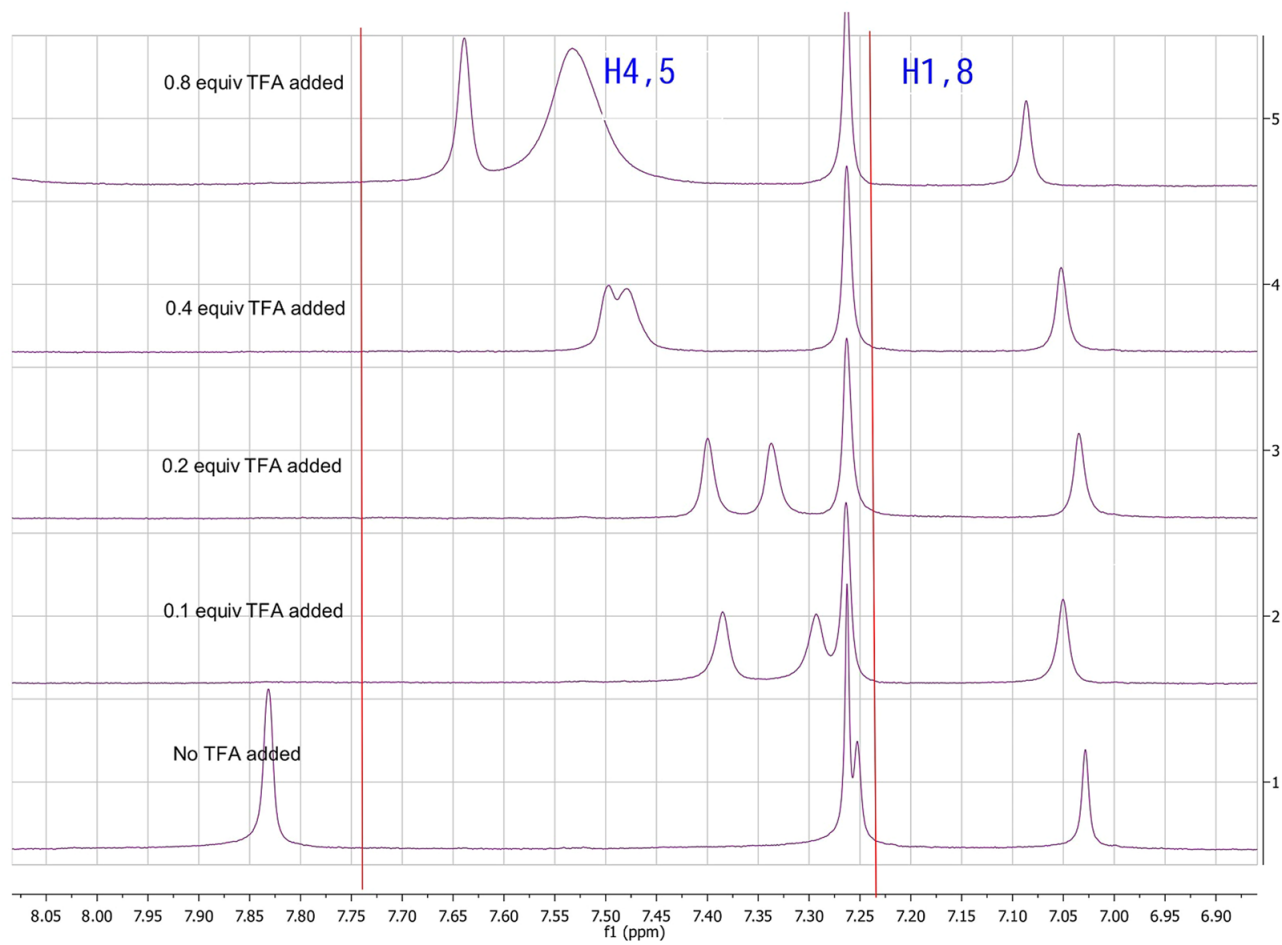
Variance of ^1^H NMR of aromatic protons with incremental amounts of TFA added (red lines indicate literature values for **1**).

We considered many factors that are known to effect chemical shift, such as sample concentration, the identity of the counteranion, the presence of acid in the solvent, the presence of impurities in the sample, and the extent of CDCl_3_ decomposition^37,38^. We also conducted further experiments to find an explanation for the observed differences. Acidity can sometimes affect ^1^H NMR spectra, especially the spectra of alkaloids, and such affects have been reported previously^17,18^ for phenanthroindolizidine alkaloids. Therefore, we conducted an acid titration as described previously^17^. The addition of incremental amounts of acid (TFA) to the sample resulted in dramatic changes in the chemical shifts, especially those of the aromatic protons (Fig. 7). However, our spectroscopic data still did not match the reported data, so we concluded that the presence of acidic impurities in the isolated sample or in the NMR solvent were not responsible for the discrepancies. Moreover, the reported data were incomplete (for example, information on test conditions, chemical shifts of aliphatic protons, ^13^C NMR data, and a copy of the ^1^H NMR spectrum were unavailable). Therefore, we suspect that the reported structure of 13a-hydroxytylophorine is incorrect and should be revised.

## Conclusions

In summary, we accomplished the first total synthesis of the reported structure of 13a-hydroxytylophorine in 17.2% overall yield. The route features an unprecedented NaBH_4_-promoted one-pot reductive cyclization cascade sequence for the construction of a nine-membered-ring azonane with a hydroxyl group at the desired position. The required oxygenated indolizidine framework was constructed via a sequence involving DMP-promoted oxidation and a subsequent unexpected Boc migration. In addition, (±)-tylophorine was synthesized via a transannular Mitsunobu reaction from a common intermediate. This total synthesis revealed that the reported structure of the naturally isolated product is incorrect. Structure modification and biological evaluation of synthetic 13a-hydroxytylophorine are in progress and will be reported in due course.

## Electronic supplementary material

supplementary information
