# Publisher Correction: Total synthesis of the reported structure of 13a-hydroxytylophorine

**DOI:** 10.1038/s41598-018-21594-5

**Published:** 2018-03-06

**Authors:** Hui Zhang, Gang Li, Bo Su, Meng Deng, Yu-Xiu Liu, Yu-Cheng Gu, Qing-Min Wang

**Affiliations:** 1 State Key Laboratory of Elemento-Organic Chemistry, Research Institute of Elemento-Organic Chemistry, College of Chemistry, Nankai University, Tianjin, 300071 People’s Republic of China; 2 Syngenta, Jealott’s Hill International Research Centre, Bracknell, Berks RG42 6EY UK; 3 Collaborative Innovation Center of Chemical Science and Engineering (Tianjin), Tianjin, 300071 People’s Republic of China

Correction to: *Scientific Reports* 10.1038/s41598-017-17015-8, published online 05 December 2017

The original version of this Article contained an error in the order of the Figures. Figures 1, 2, 3, 4, 5, 6, and 7 were published as Figures 2, 3, 4, 6, 7, 1, and 5 respectively. The Figure Legends were correct from the time of publication.

Furthermore, the orientation of the arrow linking compound 3 and (±)-tylophorine in Fig. 5, originally published as Fig. 7, was incorrect.

**Figure 5:**
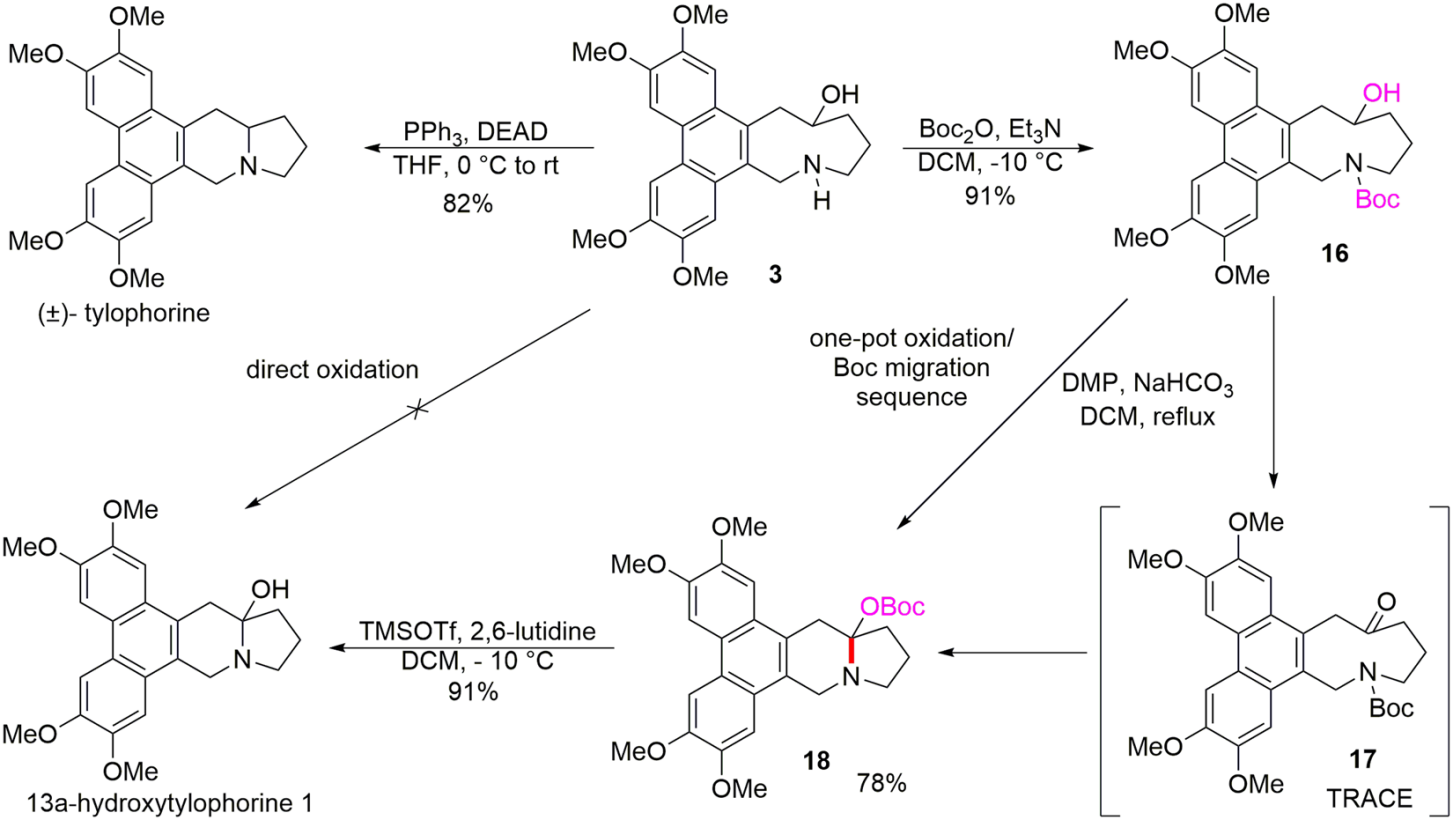
Syntheses of (±)-tylophorine and 13a-hydroxytylophorine (**1**).

These errors have now been corrected in HTML and PDF versions of this Article.

