# Supplementary material for: Total synthesis of the reported structure of 13a-hydroxytylophorine
Source: Sci Rep. 2017 Dec 5;7:16916. doi: 10.1038/s41598-017-17015-8 (PMC5716997; doi:10.1038/s41598-017-17015-8)
Supplement: Supplementary file 1 — supplementary information [file 41598_2017_17015_MOESM1_ESM.doc]

**Total synthesis of the reported structure of 13a-hydroxytylophorine**

Hui Zhang^1^, Gang Li^1^, Bo Su^1^, Meng Deng^1^, Yuxiu Liu^1^, Yucheng Gu^2^ and Qingmin Wang^1,3^*

^1^State Key Laboratory of Elemento-Organic Chemistry, Research Institute of Elemento-Organic Chemistry, Nankai University, Tianjin 300071, People’s Republic of China

^2^Syngenta, Jealott’s Hill International Research Centre, Bracknell, Berks, RG42 6EY, UK

^3^Collaborative Innovation Center of Chemical Science and Engineering (Tianjin), Tianjin 300071, People’s Republic of China

*To whom correspondence should be addressed. Tel: +86-(0)22-23503952; Fax: +86-(0)22-23503952; E-mail: wang98h@263.net or wangqm@nankai.edu.cn

**Table of content**

General Information……………………………………………………………S3

Synthesis and Characterization…………………………………………………… S3

Copies of NMR spectrum…………………………………………………………S12

Mechanism of One-pot Reductive Cyclization……………………………………S31

Variation of ^1^H NMR Resonances of Synthetic **1**………………………………S34

References………………………………………………………….……………….S38

**General Information**

All anhydrous solvents were dried and purified by standard techniques before use. All reagents were purchased from commercial suppliers without further purification. Reactions were monitored by TLC on plates (GF254) supplied by Yantai Chemicals (China) with ultraviolet (UV) detection. If not specially mentioned, flash column chromatography was performed with silica gel (200–300 mesh) supplied by Tsingtao Haiyang Chemicals (China). Melting points were determined with an X-4 binocular microscope melting-point apparatus (Beijing Tech Instruments Co, Beijing, China). NMR spectra were recorded utilizing a Bruker AV400 spectrometer with CDCl_3_ solvent and tetramethylsilane as internal standard. Chemical shifts (*δ*) are given in parts per million (ppm) and were measured downfield from internal tetramethylsilane. High-resolution mass spectra were obtained with an FT-ICR MS spectrometer (Ionspec, 7.0 T).

**Synthesis and Characterization**

**Synthesis of 4-azidobutanal**

**Scheme S1.** Synthesis of 4-azidobutanal.

4-Azidobutanal was synthesized following a multi-step procedure that was reported in the literature^1^. The synthesis route was carried out successfully in good overall yield (Scheme S1). ^1^H NMR data matched well with literature^1^ values. Spectroscopic data of 4-azidobutanal (colorless oil): ^1^H NMR (400 MHz, CDCl_3_): *δ* 9.84 (s, 1H), 3.39 (t, *J* = 6.5 Hz, 2H), 2.61 (t, *J* = 7.0 Hz, 2H), 2.04- 1.85 (m, 2H).

**Synthesis of compound 10 (5-azido-2-((tert-butyldimethylsilyl)oxy)pentanenitrile)**

To a solution of 4-azidobutanal **7** 3.39 g (30.0 mmol) in 40 mL acetonitrile was added zinc iodide 191.4 mg (0.6 mmol), and sodium cyanide 5.88 g (120.0 mmol), then 9.03 g (60.0 mmol) tert-butyldimethylsilyl chloride was added in portions under an ice-bath carefully. Then the reaction mixture was left stirring at room temperature for 4 hours, the solvent was removed *in vacuo* to give a mixture, which was diluted by water and extracted by DCM. The combined organic layers were washed with sat. aq. NaHCO_3_, water and brine, dried over Na_2_SO_4_, filtered, and concentrated *in vacuo*. A dark brown oil was obtained, which was further purified by flash chromatography on a column of silica gel (eluting with PE: EtOAc= 20: 1) to give compound **10** as a colorless oil (6.47 g, 85%). ^1^H NMR (400 MHz, CDCl_3_): *δ* 4.49 (t, *J* = 5.6 Hz, 1H), 3.37 (t, *J* = 6.6 Hz, 2H), 1.91-1.82- 1.75 (m, 2H), 1.79 (m, 2H), 0.91 (s, 9H), 0.19 (s, 3H), 0.14 (s, 3H). ^13^C NMR (100 MHz, CDCl_3_): *δ* 119.6, 61.4, 50.8, 33.4, 25.7, 24.1, 18.0, -5.2, -5.4. HRMS (ESI) Calculated for [M+H^+^] C_11_H_23_N_4_OSi^+^:255.1636. Found 255.1621.

**Synthesis of compound 11 (5-azido-1-(10-bromo-2,3,6,7-tetramethoxyphenanthren-9-yl)pentan-2-one)**

To a solution of compound **10** 0.75 g (2.95 mmol) in 20 mL anhydrous THF was added 3.40 mL LiHMDS (1M in THF, 3.4 mmol) dropwise at -78 ℃ under argon. The resulting mixture was allowed to warm up to -40 ℃ after 10 mins. Phenanthryl bromide **6** 1.00 g (2.13 mmol) was added into the above mixture through a syringe as a suspension in cold 10 mL anhydrous THF after 1 hour. Then the mixture was allowed to warm up to room temperature naturally and kept stirring. Sat. aq. NH_4_Cl was added to quench the reaction after 6 hours. The reaction solvent was removed *in vacuo* to give a residue, which was added 50 mL water and 1N HCl till pH = 7, and extracted by DCM. The combined organic layers were washed with water and brine, dried over Na_2_SO_4_, filtered, and concentrated *in vacuo*. A dark brown oil was obtained, which was directly used for the next transformation without further purification.

To a solution of the above oil in anhydrous 30 mL THF was added 4.3 mL TBAF (1M in THF, 4.30 mmol) at 0 ℃. The reaction mixture was kept stirring for 2 hours, the solvent was removed *in vacuo* to give a residue, which was diluted by aq. NH_4_Cl and extracted by DCM. The combined organic layers were washed with water and brine, dried over Na_2_SO_4_, filtered, and concentrated *in vacuo*. A dark brown oil was obtained, which was further purified by flash chromatography on a column of silica gel (eluting with PE: EtOAc= 4: 1) to give compound **11** (0.82 g, 77%) as a white solid. MP = 152-154 ℃.^1^H NMR (400 MHz, CDCl_3_): *δ* 7.84 (s, 2H), 7.80 (s, 1H), 7.79 (s, 1H), 4.53 (s, 2H), 4.14 (s, 3H), 4.12 (s, 3H), 4.10 (s, 3H), 4.02 (s, 3H), 3.20 (t, *J* = 8.0 Hz, 2H), 2.53 (t, *J* = 7.0 Hz, 2H), 1.83- 1.76 (m, 2H). ^13^C NMR (100 MHz, CDCl_3_): *δ* 207.6, 149.8, 149.5, 149.4, 149.3, 127.2, 125.8, 125.0, 124.1, 123.5, 109.4, 105.4, 103.2, 102.7, 56.2, 56.0, 56.0, 55.9, 50.5, 50.0, 37.8, 22.8. HRMS (ESI) Calculated for [M+NH_4_^+^] C_23_H_25_BrN_3_O_5_^+^: 519.1238. Found 519.1229.

**Synthesis of compound 5 (5-azido-1-(10-bromo-2,3,6,7-tetramethoxyphenanthren-9-yl)pentan-2-ol)**

To a solution of the above ketone **11** 2.26 g (4.50 mmol) in 20 mL DCM and 40 mL MeOH was added NaBH_4_ 0.34 g (9.00 mmol) in portions under an ice-bath. After the reaction mixture was kept stirring for 30 mins, it was diluted by 1 N HCl till pH = 6, and then extracted by DCM. The combined organic layer was washed with water and brine, dried over Na_2_SO_4_, filtered, and concentrated *in vacuo*. A yellow solid was obtained, which was further purified by flash chromatography on a column of silica gel (eluting with PE: EtOAc= 1: 1) to give the corresponding alcohol **5** (2.20g, 97%) as a white solid. MP: 130- 132 ℃.^1^H NMR (400 MHz, CDCl_3_): *δ* 7.75 (s, 1H), 7.67 (s, 1H), 7.64 (s, 1H), 7.48 (s, 1H), 4.23- 4.14 (m, 1H), 4.08 (s, 3H), 4.05 (s, 6H), 4.03 (s, 3H), 3.58- 3.40 (m, 2H), 3.37 (t, *J* = 6.4 Hz, 2H), 1.93- 1.8 (m, 1H), 1.82- 1.72 (m, 4H).^13^C NMR (100MHz, CDCl_3_): *δ* 149.5, 149.4, 149.2, 148.9, 130.7, 126.2, 125.2, 124.7, 124.3, 122.9, 109.2, 106.2, 103.1, 102.7, 71.9, 56.1, 56.0, 56.0, 55.9, 51.5, 41.7, 34.5, 32.2, 25.5.HRMS (ESI) Calculated for [M+NH_4_^+^] C_23_H_30_BrN_4_O_5_^+^: 521.1394. Found 521.1383.

**Synthesis of compound 12 (5-azido-1-(10-bromo-2,3,6,7-tetramethoxyphenanthren-9-yl)pentan-2-yl dimethylcarbamate)**

To a solution of the above alcohol **5** 1.01 g (2.00 mmol) in 20 mL THF was added 2.4 mL KHMDS (1M in THF, 2.40 mmol) dropwise at -15 ℃ under argon. The resulting mixture was kept stirring for 30 mins at the same temperature before 0.18 mL dimethylcarbamoyl chloride (4.00 mmol) was added. Then the reaction mixture was kept stirring for 1 hour, before quenched by sat. aq. NH_4_Cl. The solvent was removed *in vacuo* to give a residue, which was treated with 1N HCl till pH = 7, and extracted by DCM. The combined organic layers were washed with water and brine, dried over Na_2_SO_4_, filtered, and concentrated *in vacuo*. A yellow solid was obtained, which was further purified by flash chromatography on a column of silica gel (eluting with PE: EtOAc= 2: 1) to give the corresponding carbonate **12** (1.04 g, 90%) as a white solid. MP: 133- 135 ℃. ^1^H NMR (400 MHz, CDCl_3_): *δ* 7.94 (s, 1H), 7.83 (s, 1H), 7.76 (s, 2H), 5.24- 5.18 (m, 1H), 4.16 (s, 3H), 4.12 (s, 3H), 4.12 (s, 3H), 4.08 (s, 3H), 3.83- 3.78 (m, 1H), 3.75- 3.69 (m, 1H), 3.24- 3.12 (m, 2H), 2.91 (s, 6H), 2.80 (s, 1H）, 1.96- 1.87 (m, 1H), 1.81- 1.70 (m, 1H), 1.65- 1.57 (m, 1H), 1.54- 1.45 (m, 1H).^13^C NMR (100MHz, CDCl_3_): *δ* 156.3, 149.4, 149.4, 149.2, 149.2, 129.8, 126.2, 125.2, 124.8, 124.0, 123.2, 109.6, 106.6, 102.9, 102.7, 74.8, 56.6, 56.1, 56.0, 55.9, 51.2, 39.1, 36.4, 36.0, 30.4, 25.6.HRMS (ESI) Calculated for [M+H^+^] C_26_H_32_BrN_4_O_6_^+^: 575.1500. Found 575.1488.

**Synthesis of compound 4 (3-(3-azidopropyl)-6,7,10,11-tetramethoxy-3,4-dihydro-1H-dibenzo[f,h]isochromen-1-one)**

To a solution compound **12** 1.35 g (2.35 mmol) in 40 mL THF was added 1.90 mL *n*-BuLi (1.6 M in hexane, 3.01 mmol) dropwise at -78 ℃ under argon. The resulting mixture was kept stirring for 2 hours at the same temperature, before quenched by sat. aq. NH_4_Cl. Then the reaction solvent was removed *in vacuo* to give a residue, which was diluted by sat.aq NH_4_Cl, and extracted by DCM. The combined organic layers were washed with water and brine, dried over Na_2_SO_4_, filtered, and concentrated *in vacuo*. A yellow solid, namely crude **13**, was obtained, which was directed used for next step without further purification.

To a solution of the crude **13** in 30 mL toluene was added (*D*)-camphorsulfonic acid 10.90 g (4.70 mmol). Then the resulting mixture was heated to 60℃ and kept stirring for 4 hours. The reaction solvent was removed *in vacuo* to give a dark brown oil, which was diluted by water and extracted by DCM. The combined organic layers were washed with sat. aq. NaHCO_3_, water and brine, dried over Na_2_SO_4_, filtered, and concentrated *in vacuo*. A dark brown solid was obtained, which was further purified by flash chromatography on a column of silica gel (eluting with PE: EtOAc= 1: 2) to give corresponding azido lactone **4** (0.66 g, 2 steps, 63%) as a white solid. MP: 211- 213 ℃. ^1^H NMR (400 MHz, CDCl_3_): *δ* 8.68 (s, 1H), 7.77 (s, 1H), 7.74 (s, 1H), 7.25 (s, 1H), 4.61- 4.51 (m, 1H), 4.15 (s, 3H), 4.12 (s, 3H), 4.07 (s, 3H), 4.06 (s, 3H), 3.48- 3.42 (m, 2H), 3.38- 3.34 (m, 1H), 3.18- 3.11 (m, 1H), 2.09- 1.98 (m, 3H), 1.91-1.83 (m, 1H). ^13^C NMR (100 MHz, CDCl_3_): *δ* 165.7, 151.5, 149.6, 149.2, 149.1, 136.6, 127.7, 124.5, 124.1, 122.4, 117.1, 107.5, 104.8, 103.1, 102.6, 75.9, 56.1, 56.0, 56.0, 56.0, 51.2, 32.1, 31.4, 24.8.HRMS (ESI) Calculated for [M+H^+^] C_24_H_26_N_3_O_6_^+^: 452.1816. Found 452.1809.

**Characterization of intermediate 13 (10-(5-azido-2-hydroxypentyl)-2,3,6,7-tetramethoxy-N,N-dimethylphenanthrene-9-carboxamide)**

Pure sample of **13** was obtained via further purification by flash chromatography on a column of silica gel (eluting with DCM: MeOH= 20: 1)as a white solid. MP: 126- 128 ℃. ^1^H NMR (400 MHz, CDCl_3_): *δ* 7.84 (s, 1H), 7.81 (s, 1H), 7.31 (s, 1H), 6.91 (s, 1H),4.55 (d, *J =* 4.8Hz, 1H), 4.14 (s, 3H), 4.12 (s, 3H), 4.04 (s, 3H), 3.97 (s, 3H), 3.45 (dd, *J =*  2.0, 14.4 Hz, 1H), 3.41- 3.37 (m, 2H), 3.34 (s, 3H), 2.79 (s, 3H), 2.72 (dd, *J =* 9.9, 14.4 Hz, 1H), 1.95- 1.70 (m, 4H). ^13^C NMR (100 MHz, CDCl_3_): *δ* 172.7, 149.6, 149.6, 149.4, 149.1, 130.5, 127.8, 125.3, 124.5, 124.1, 121.9, 105.1, 105.0, 103.5, 103.3, 69.7, 56.1, 56.1, 55.9, 55.9, 51.5, 39.0, 38.5, 36.1, 35.0, 25.3. HRMS (ESI) Calculated for [M+H^+^] C_26_H_33_N_4_O_6_^+^ 497.2395: Found 497.2394.

**Synthesis of compound 14 (3-(3-aminopropyl)-6,7,10,11-tetramethoxy-3,4-dihydro-1H-dibenzo[f,h]isochromen-1-one)**

To a solution of azido lactone **4** 0.90 g (2.00 mmol) in 10 mL THF and 40 mL MeOH was added 10% Palladium on activated charcoal 0.09g. Then the mixture was kept stirring under a balloon of hydrogen at room temperature for 5 hours. The reaction solution was filtered through a pad of silica gel. The resulting filtrate was concentrated *in vacuo* to give a dark brown oil, which was further purified by flash chromatography on a column of silica gel (eluting with DCM: MeOH= 10: 1) to give the desired amino lactone **14** (0.65 g, 77%) as a yellow solid. MP: 226- 228 ℃. ^1^H NMR (400 MHz, CDCl_3_): *δ* 8.66 (s, 1H), 7.74 (s, 1H), 7.71 (s, 1H), 7.21 (s, 1H), 4.61- 4.51 (m, 1H), 4.14 (s, 3H), 4.11 (s, 3H), 4.05 (s, 3H), 4.03 (s, 3H), 3.34 (dd, *J* = 16.6, 2.8 Hz, 1H), 3.11 (dd, *J* = 16.6, 12.0 Hz, 1H), 2.91 (t, *J* = 6.6 Hz, 1H), 2.41 (br, 2H), 2.11- 2.01 (m, 1H), 2.01- 1.87 (m, 2H), 1.86- 1.75 (m, 1H).^13^C NMR (100 MHz, CDCl_3_): *δ* 165.8, 151.2, 149.6, 148.9, 148.9, 136.8, 127.4, 124.2, 123.9, 122.2, 116.9, 107.4, 104.7, 102.9, 102.4, 76.3, 56.0, 55.9, 55.8,55.8, 41.7, 32.2, 31.1, 28.7.HRMS (ESI) Calculated for [M+H^+^] C_24_H_28_NO_6_^+^: 426.1911. Found 426.1915.

**Synthesis of compound 3 (9,10,13,14-tetramethoxy-2,3,4,5,6,7-hexahydro-1H-phenanthro[9,10-c]azonin-6-ol)**

To a solution of amino lactone **14** 0.22 g (0.52 mmol) in 4 mL THF and 16 mL MeOH was added NaBH_4_ (98.4 mg, 2.60 mmol, 5 equiv) at 0 ℃ before the mixture was heated to 50 ℃. After the resulting mixture was kept stirring for one hour, the reaction was quenched with 1N HCl. Then sat. aq. Na_2_CO_3_ was added till pH above 7. The mixture was extracted by DCM. The combined organic layer was washed with water and brine, dried over Na_2_SO_4_, filtered, and concentrated *in vacuo*. A solid was obtained, which was further purified by flash chromatography on a column of silica gel (eluting with DCM: MeOH= 5: 1) to give the desired amino alcohol**3** (0.17 g, 83%) as a white solid. MP: 192-195 ℃.^1^H NMR (400 MHz, CDCl_3_): *δ* 7.86 (s, 1H), 7.82 (s, 1H), 7.71 (s, 1H), 7.36 (s, 1H), 5.14 (q, *J* = 12.6 Hz, 2H), 4.12 (s, 3H), 4.11 (s, 4H), 4.08 (s, 3H), 4.03 (s, 3H), 3.86- 3.80 (m, 1H), 3.41 (d, *J* = 5.5 Hz, 2H), 3.08- 2.97 (m, 1H), 2.69- 2.58 (m, 1H), 2.18- 2.08 (m, 1H), 2.02- 1.91 (m, 1H), 1.84- 1.72 (m, 1H), 1.53- 1.41 (m, 1H). ^13^C NMR (100 MHz, CDCl_3_): *δ* 149.0, 148.8, 148.7, 148.4, 132.2, 130.6, 125.8, 125.3, 125.3, 124.1, 105.6, 105.6, 103.5, 103.1, 70.6, 58.3, 56.1, 56.0, 56.0, 55.8, 41.5, 39.5, 37.2, 30.2.HRMS (ESI) Calculated for [M+H^+^] C_24_H_30_NO_5_^+^: 412.2118. Found 412.2116.

**One-pot synthesis of compound 3 from compound 4**

To a solution of azido lactone **4** 0.23 g (0.51 mmol) in 4 mL THF and 16 mL MeOH was added NaBH_4_ (193.8 mg, 5.10 mmol, 10 equiv) at 0 ℃ before the mixture was heated to 50 ℃. On complete consumption of starting material by TLC (4 hours), according to the same procedures of workup, the desired amino alcohol**3** was obtained (0.16 g, 74%).

**Synthesis of tylophorine**

To a solution of amino alcohol **3** 0.10 g (0.24 mmol) in 20 mL DCM were added 95.6 μL diisopropyl azodicarboxylate (DIAD, 0.48 mmol) and PPh_3_ 0.13 g (0.48 mmol) at 0 °C. Then the reaction mixture was kept stirring at room temperature overnight. The reaction solvent was removed *in vacuo* to give a dark residue, which was directly purified by flash chromatography on a column of silica gel (eluting with DCM: MeOH= 20: 1) to give tylophorine (78.4 mg, 82%) as a yellow solid. MP: 282- 284 °C. ^1^H NMR (400 MHz, CDCl_3_): *δ* 7.80 (s, 1H), 7.80 (s, 1H), 7.13 (s, 2H), 4.60 (d, *J* = 14.6 Hz, 2H), 4.11 (s, 6H), 4.05 (s, 3H), 4.04 (s, 3H), 3.64 (d, *J* = 14.6 Hz, 1H), 3.47 (t, *J* = 7.7 Hz, 1H), 3.33 (dd, *J* = 15.7, 2.5 Hz, 1H), 2.94- 2.81 (m, 1H), 2.51- 2.40 (m, 2H), 2.29- 2.16 (m, 1H), 2.12- 1.98 (m, 1H), 1.98- 1.85 (m, 2H), 1.84- 1.69 (m, 1H). ^13^C NMR (100 MHz, CDCl_3_): *δ* 148.7, 126.3, 126.0, 125.8, 124.3, 124.1, 123.6, 123.4, 103.9, 103.4, 103.3, 103.1, 60.2, 56.0, 56.0, 55.9, 55.9, 55.2, 54.0, 33.8, 31.3, 21.6.

**Synthesis of compound 16 (tert-butyl 6-hydroxy-9,10,13,14-tetramethoxy-1,3,4,5,6,7-hexahydro-2H-phenanthro[9,10-c]azonine-2-carboxylate)**

To a solution of amino-alcohol **3** 0.23 g (0.5 mmol) in 30 mL DCM and 0.12 mL triethyl amine (0.84 mmol) was added a solution of di-tert-butyl dicarbonate 0.18 g (0.84 mmol) in 20 mL DCM at -10 ℃. The resulting mixture was kept stirring at the same temperature for 2 hours. The reaction solvent was then removed *in vacuo* to give a solid, which was directly purified by flash chromatography on a column of silica gel (eluting with DCM: MeOH= 20: 1) to give the desired compound **16** (0.26 g, 91%) as a white solid. MP: 181-183 ℃. ^1^H NMR (400 MHz, CDCl_3_): *δ* 7.83 (s, 1H), 7.80 (s, 1H), 7.67 (s, 1H), 7.31 (s, 1H), 5.15 (dd, *J* = 29.3, 12.6 Hz, 2H), 4.68 (brs, 1H), 4.12 (s, 3H), 4.11 (s, 3H), 4.07 (s, 3H), 4.02 (s, 3H), 3.49- 3.30 (m, 2H), 3.27- 3.13 (m, 2H), 1.85- 1.70 (m, 4H), 1.43 (s, 9H). ^13^C NMR (100 MHz, CDCl_3_): *δ* 156.3, 149.1, 149.0, 148.8, 148.6, 131.8, 129.7, 125.6, 125.2, 125.0, 124.2, 105.4, 105.4, 103.4, 103.1, 79.4, 70.7, 58.6, 56.0, 56.0, 55.9, 55.9, 40.2, 36.5, 35.1, 28.4, 26.6. HRMS (ESI) Calculated for [M+H^+^] C_29_H_38_NO_7_^+^: 512.2643. Found 512.2640.

**Synthesis of compound 18 (13a-*O*-Boc-tylophorine)**

To a solution of compound **16** 100 mg (0.20 mmol) in 20 mL DCM and NaHCO_3_ 82.2 mg (0.92 mmol, 5.0 equiv) was added Dess-Martin Periodinane 130 mg (0.31mmol, 1.5 equiv). The resulting mixture was heated to reflux and kept stirring for 2 hours. The reaction was quenched by water. The water phase was exacted with DCM. The combined organic phase was washed with sat.aq sodium thiosulfate and brine, dried over Na_2_SO_4_, filtered, and concentrated *in vacuo*. A yellow solid was obtained, which was further purified by flash chromatography on a column of silica gel (eluting with DCM: MeOH= 50: 1) to give the 13a-*O*-Boc-tylophorine **18** (78.0 mg, 78%) as a white solid. MP: 214- 216 ℃. ^1^H NMR (400 MHz, CDCl_3_): *δ* 7.84 (d, *J* = 2.8 Hz, 2H), 7.24 (s, 1H), 6.99 (s, 1H), 5.22 (t, *J* = 16.0 Hz, 2H), 4.13 (s, 3H), 4.12 (s, 3H), 4.07 (s, 3H), 4.02 (s, 3H), 3.76- 3.70 (m, 1H), 3.65- 3.58 (m, 1H), 3.13 (m, 1H), 2.36- 2.30 (m, 1H), 2.07- 1.98 (m, 1H), 1.90- 1.79 (m, 2H), 1.45- 1.44 (m, 1H), 1.40- 1.14 (m, 9H). ^13^C NMR (100 MHz, CDCl_3_): *δ* 154.0, 148.5, 148.4, 148.3, 125.1, 124.8, 123.6, 123.1, 122.5, 103.6, 103.3, 103.1, 102.1, 91.6, 79.8, 63.5, 60.4, 56.1, 56.0, 55.8, 55.8, 48.5, 32.5, 28.4, 20.9.HRMS (ESI) Calculated for [M+Na^+^] C_29_H_35_NNaO_7_^+^: 532.2306. Found 532.2297.

**Synthesis of 13a-hydroxytylophorine 1**

To a solution of 13a-*O*-Boc-tylophorine **18** 31 mg (0.06 mmol) in 10 mL anhydrous DCM and 68.5 μL 2, 6- lutidine (0.60 mmol, 10 equiv) was added 53.3 μL trimethylsilyl trifluoromethanesulfonate (TMSOTf, 0.30 mmol, 5 equiv) at -10 ℃. The resulting mixture was kept stirring at the same temperature for 20 mins. The reaction was quenched by sat.aq NaHCO_3_. The water phase was exacted with DCM. The combined organic phase was washed with water and brine, dried over Na_2_SO_4_, filtered, and concentrated *in vacuo*. A yellow solid was obtained, which was further purified by flash chromatography on a column of basic silica gel (eluting with DCM: MeOH= 50: 1) to give the 13a-hydroxytylophorine (22.0 mg, 91%) as a white solid. MP > 226 ℃ decompose. (Ref^2^: 270- 272℃). ^1^H NMR (400 MHz, CDCl_3_): *δ* 7.78 (s, 2H), 7.19 (s, 1H), 6.98 (s, 1H), 5.18 (dd, *J* = 42.1, 15.4 Hz, 1H), 4.11 (s, 6H), 4.01 (s, 6H), 3.34- 3.33 (m, 1H), 3.29- 3.23 (m, 1H),3.16- 3.10 (m, 2H), 2.25- 2.19 (m, 2H: D_2_O exchangeable, 1 H), 2.13- 2.04 (m, 1H), 2.00- 1.91 (m, 1H), 1.88- 1.81 (m, 1H).^13^C NMR (100 MHz, CDCl_3_): *δ* 148.77, 148.75, 148.59, 148.56, 125.34, 124.41, 124.39, 123.64, 123.24, 122.64, 103.43, 103.39, 103.33, 102.42, 92.41, 62.07, 56.05, 56.03, 55.84, 55.84, 44.98, 35.71, 35.44, 23.41.HRMS (ESI) Calculated for [M+H^+^] C_24_H_28_NO_5_^+^: 410.1962. Found 410.1960.

According to ^1^H NMR, ^13^C NMR and 2-D NMR spectrum, we got a detailed assignment of chemical shifts of synthetic 13a-hydroxytylophorine is depicted in the Figure S1.

Figure S1. Assignment of chemical shifts of synthetic 13a-hydroxytylophorine.

**Copies of NMR spectrum**


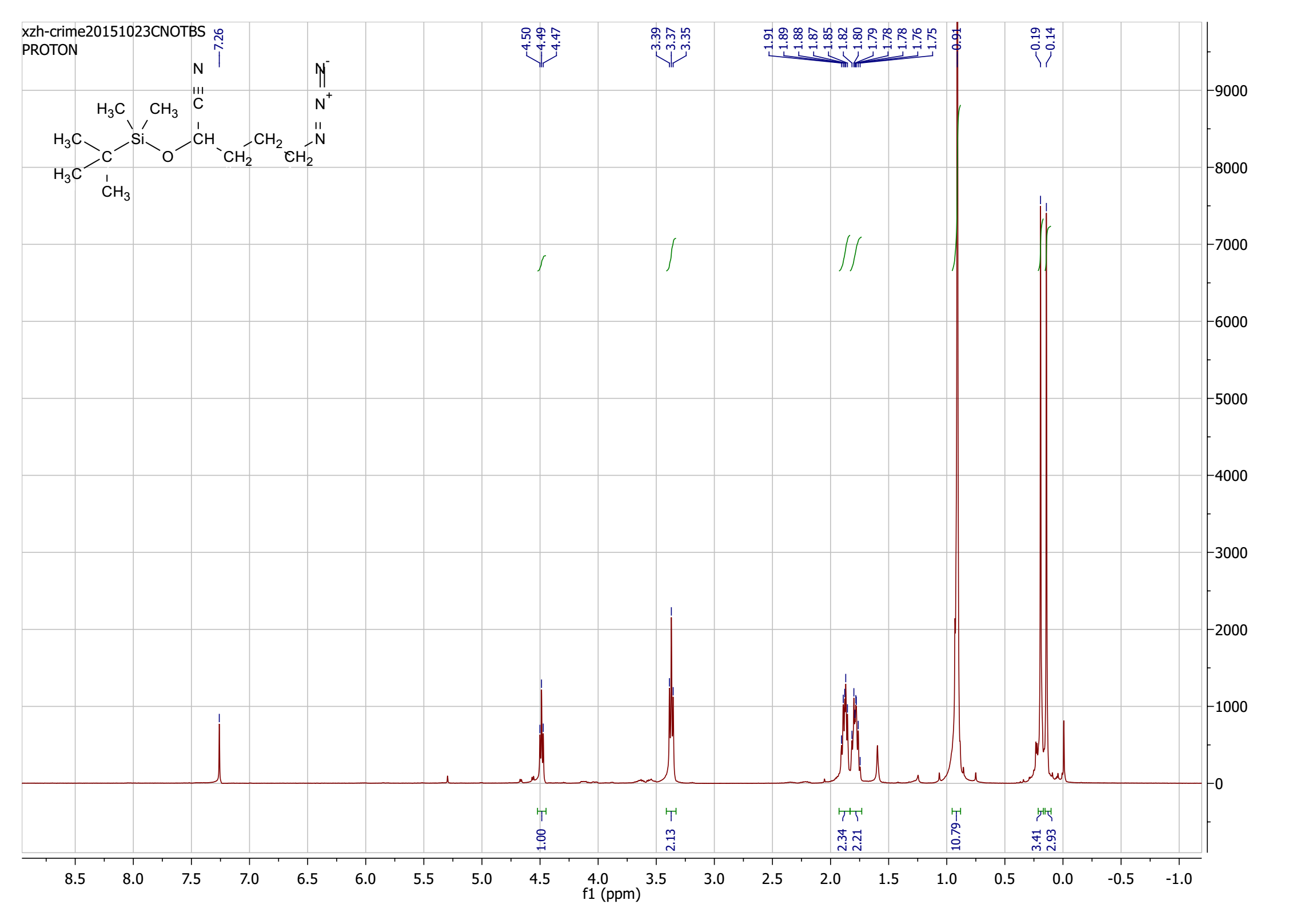


^1^H NMR spectrum of compound **10**


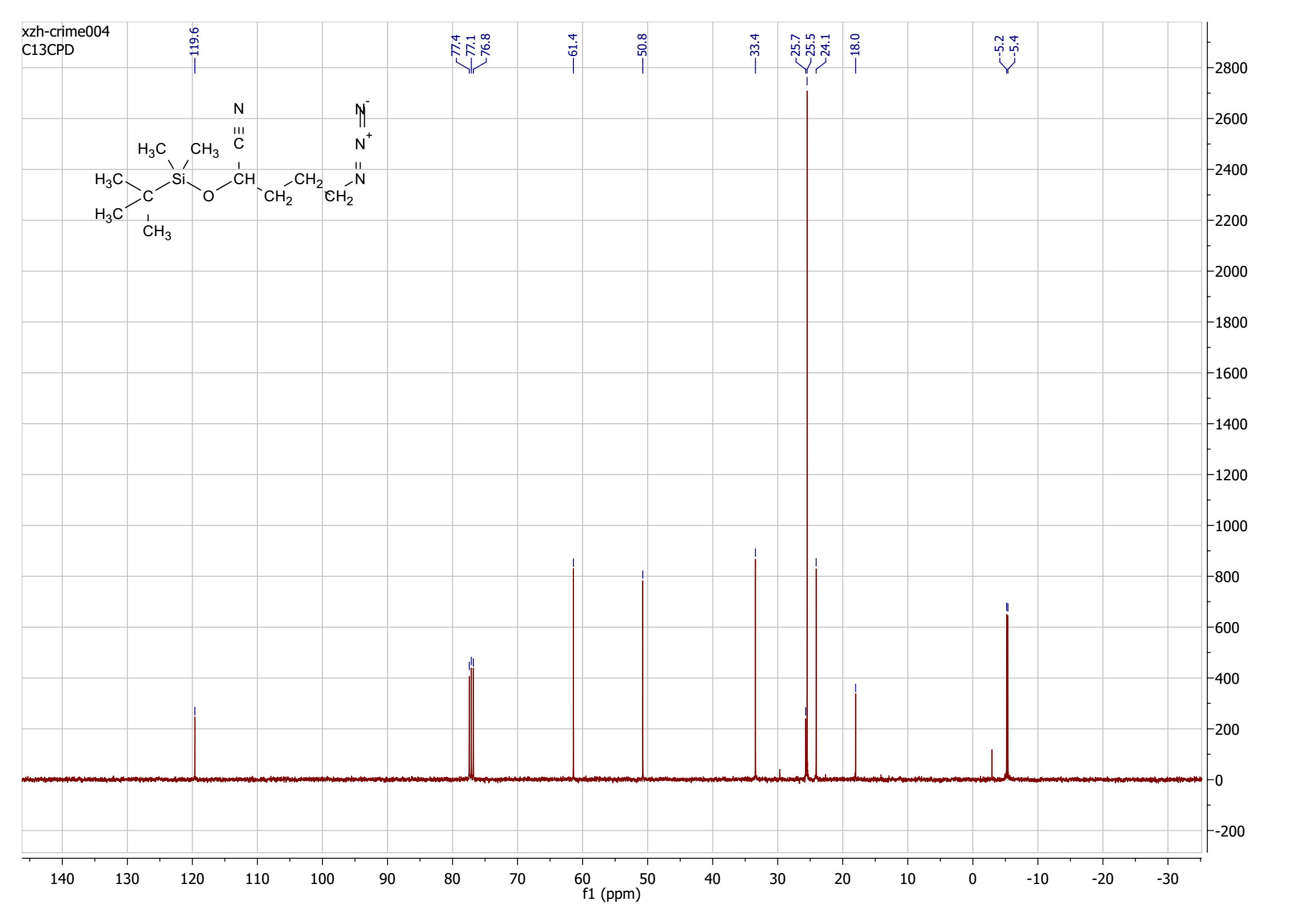


^13^C NMR spectrum of compound **10**


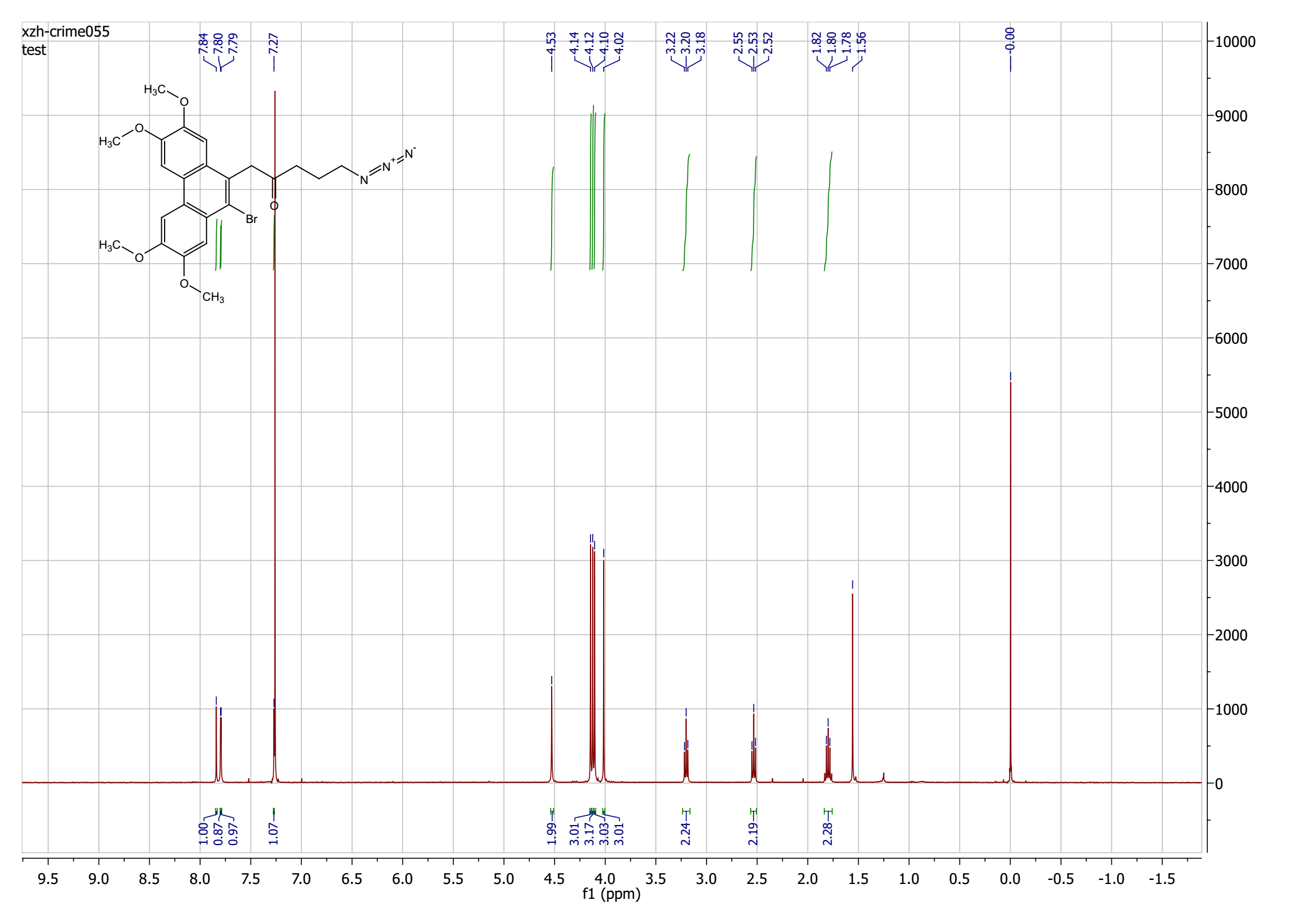


^1^H NMR spectrum of compound **11**


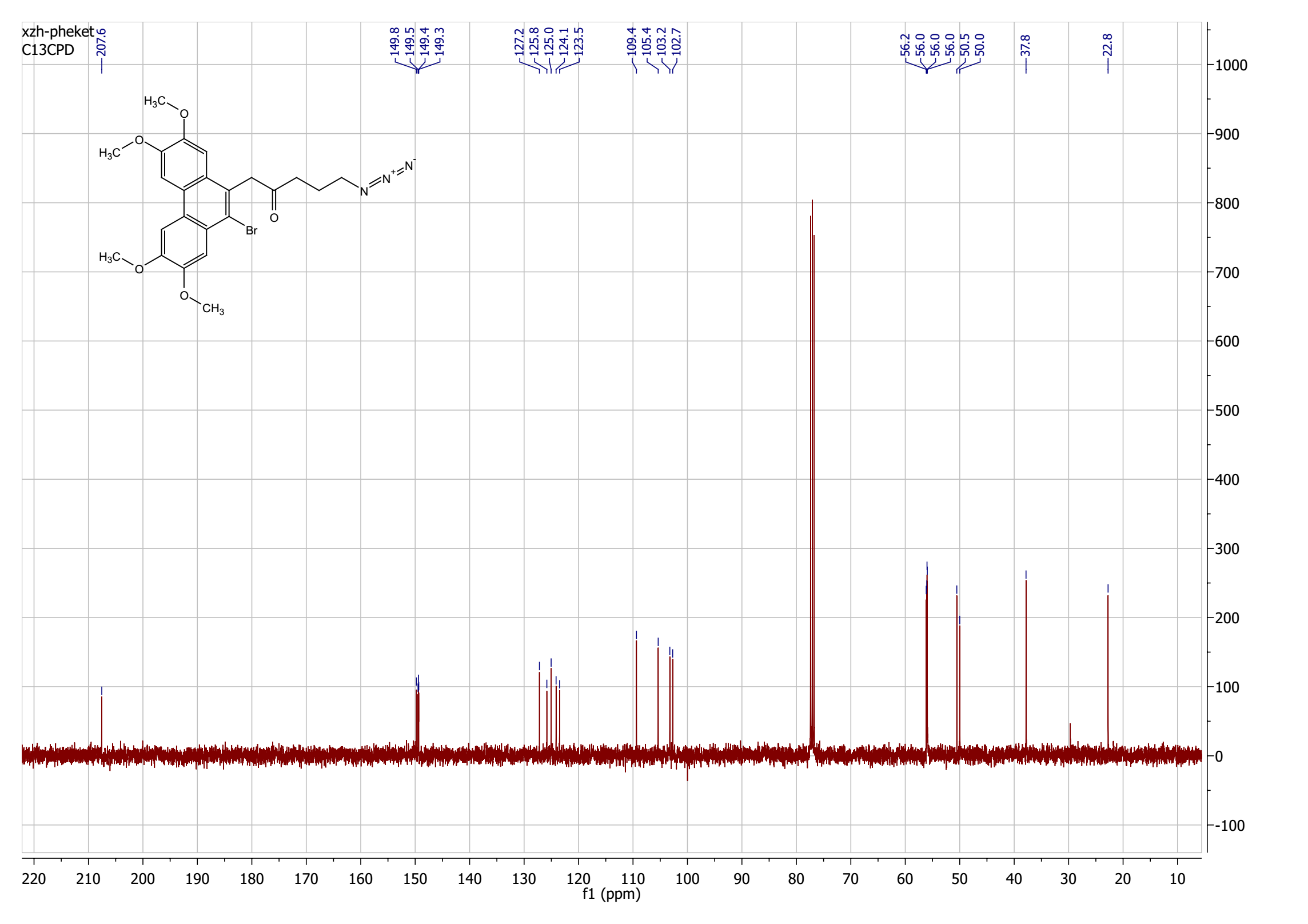


^13^C NMR spectrum of compound **11**


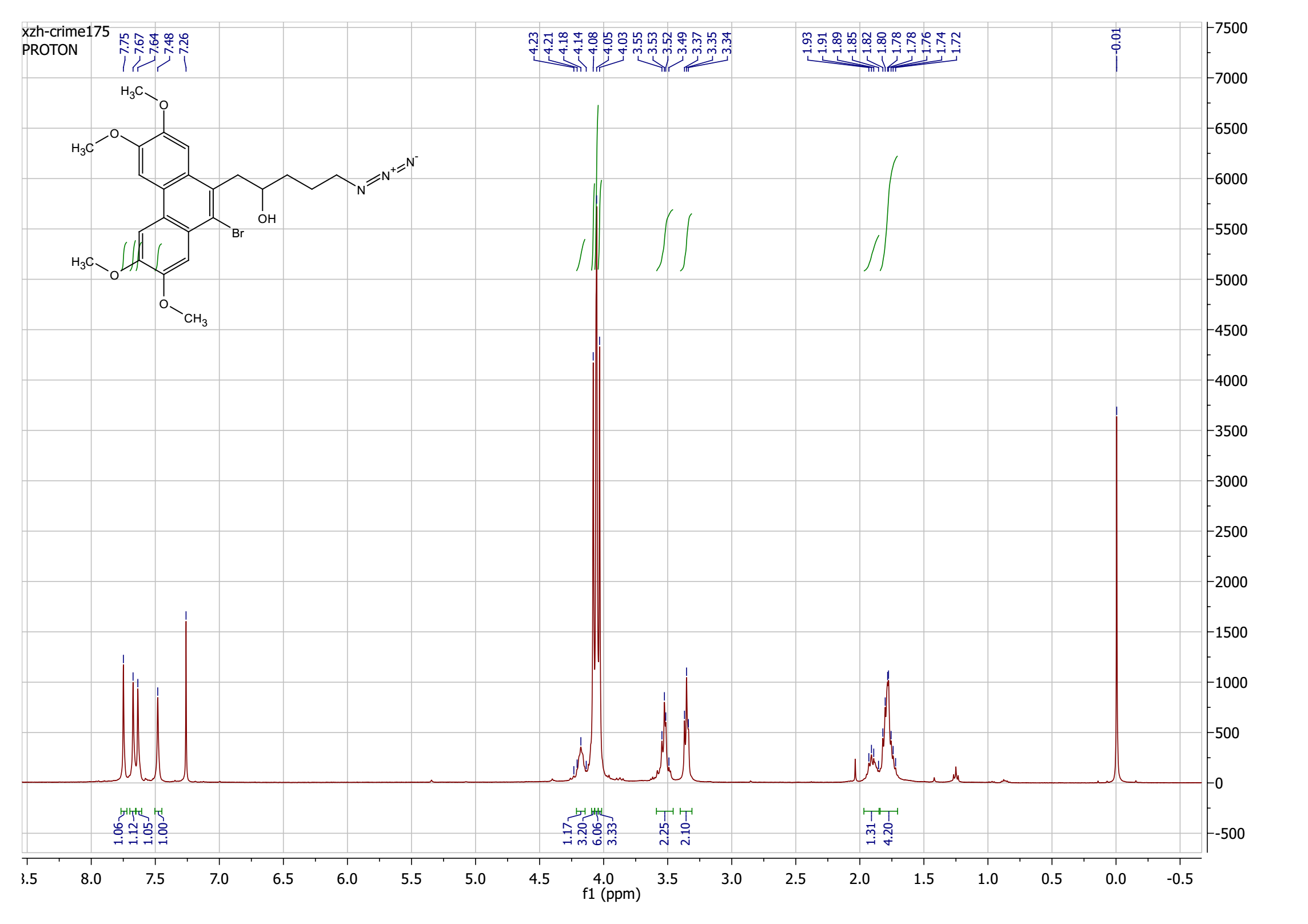


^1^H NMR spectrum of compound **5**


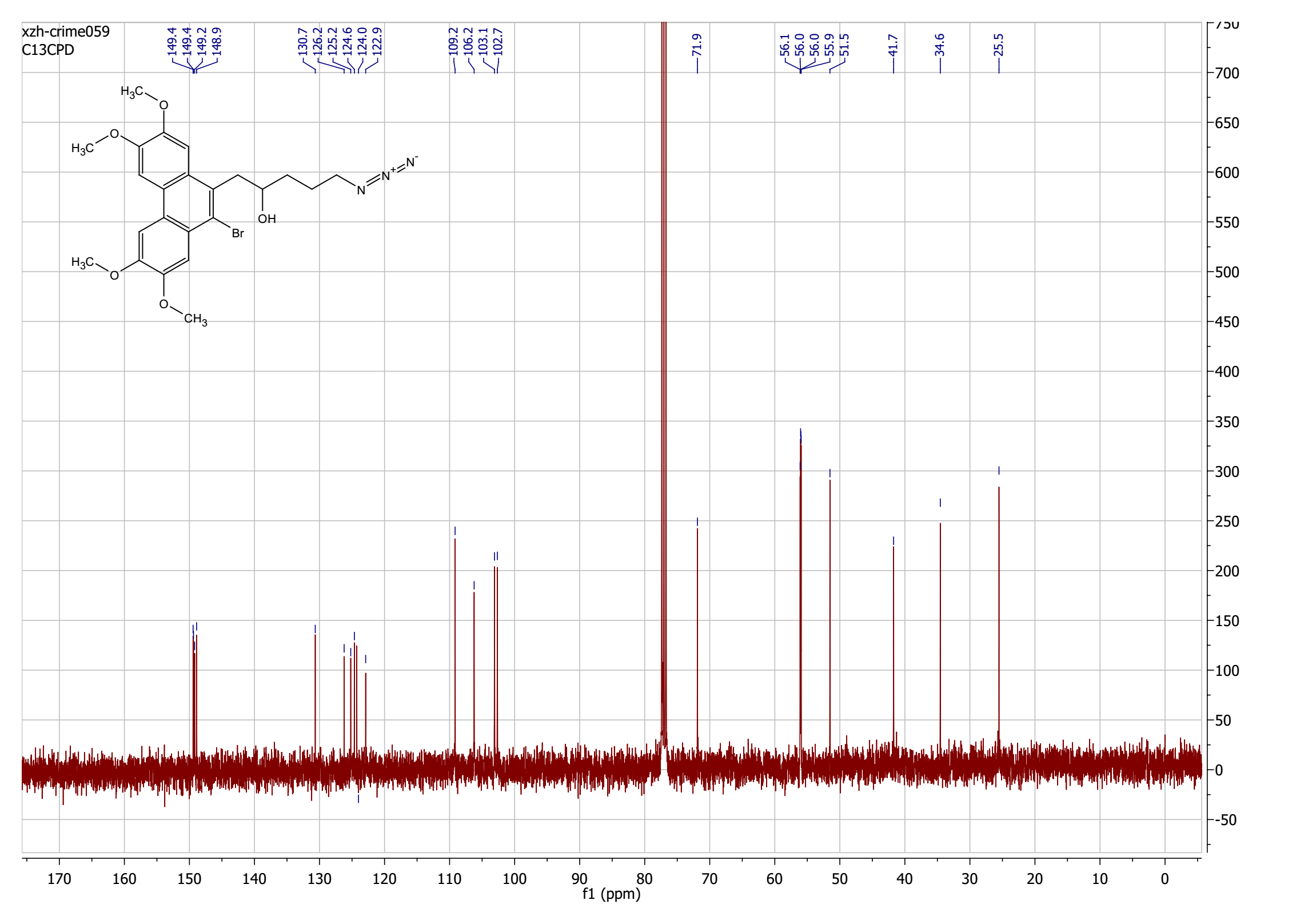


^13^C NMR spectrum of compound **5**


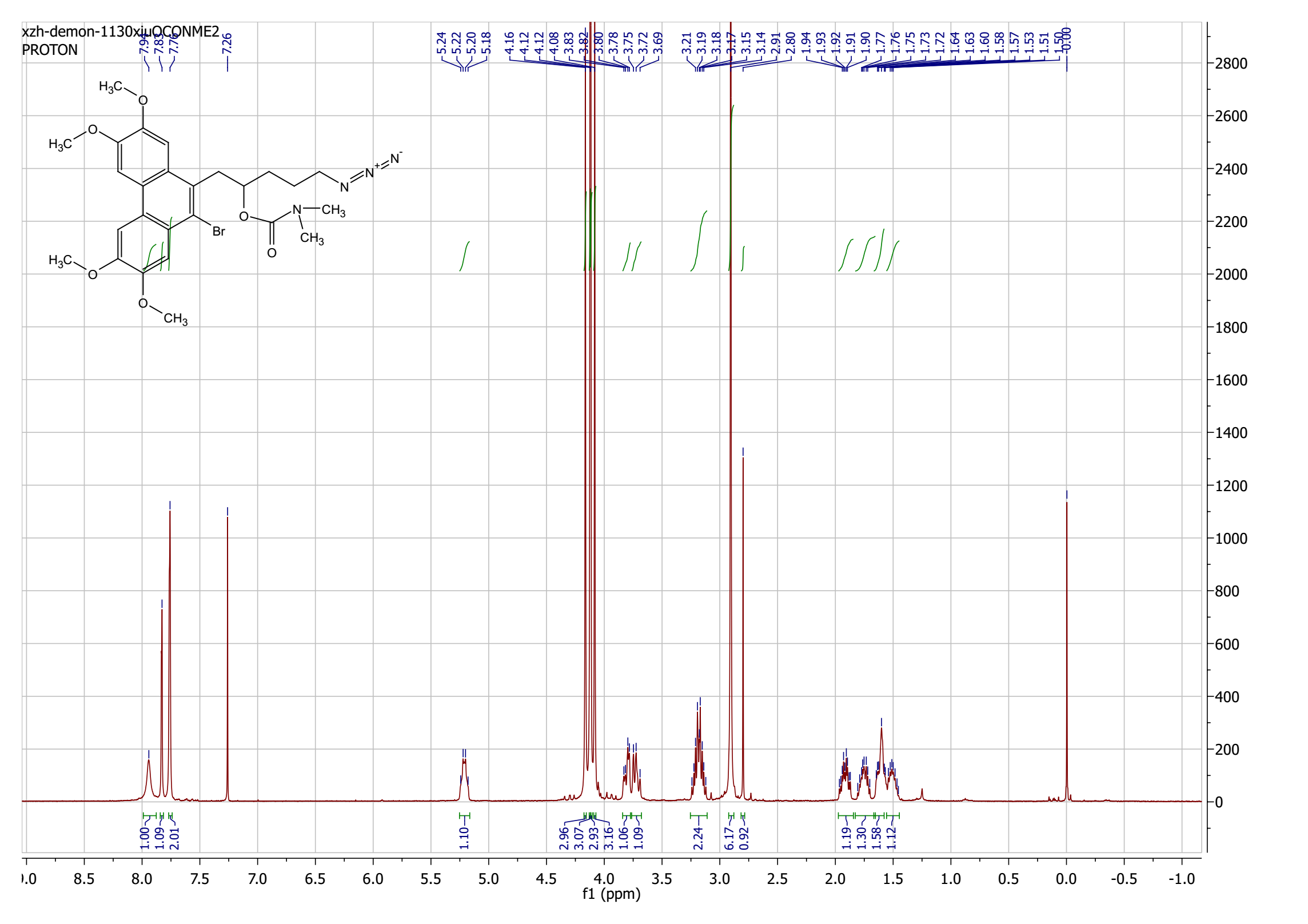


^1^H NMR spectrum of compound **12**


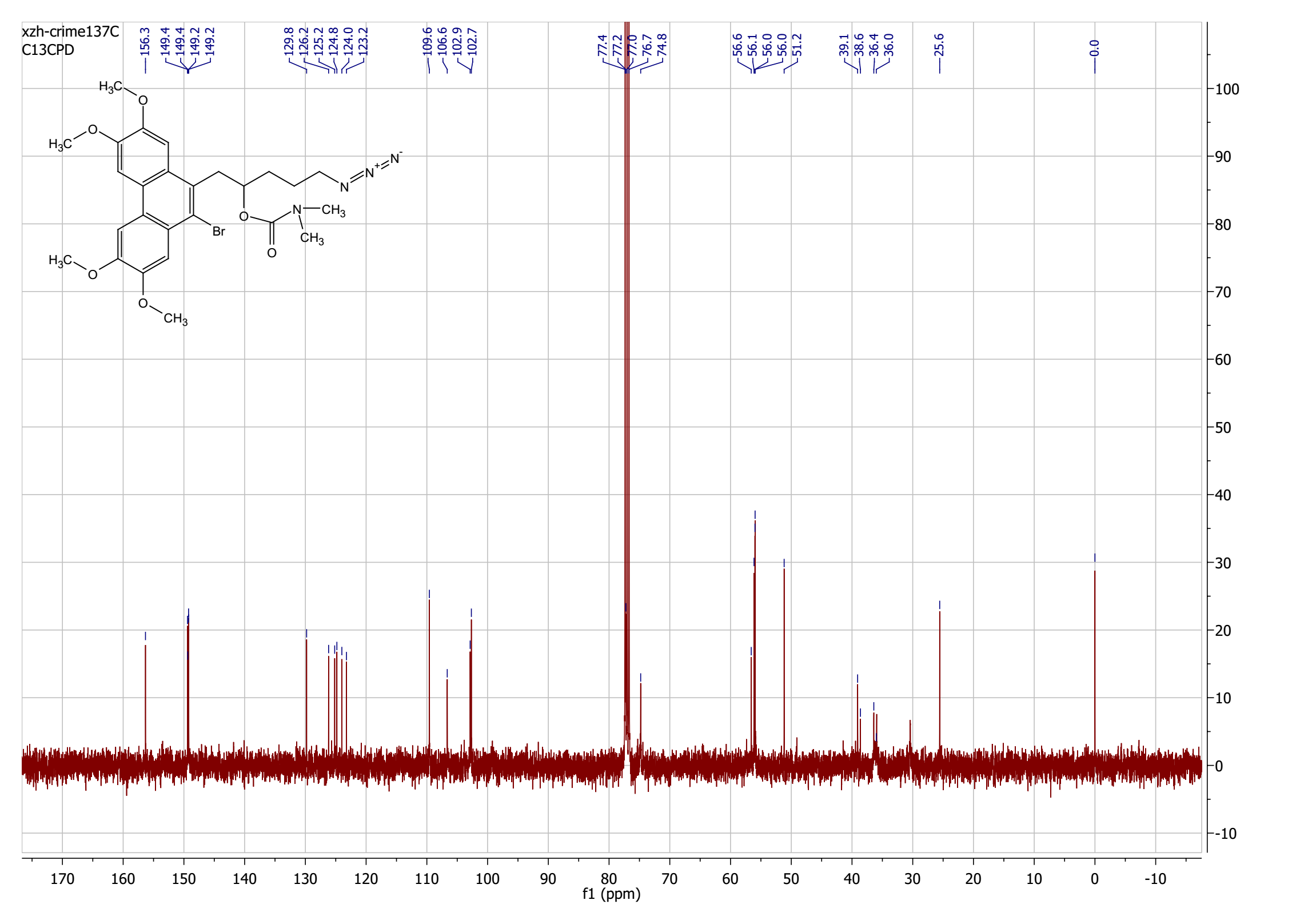


^13^C NMR spectrum of compound **12**


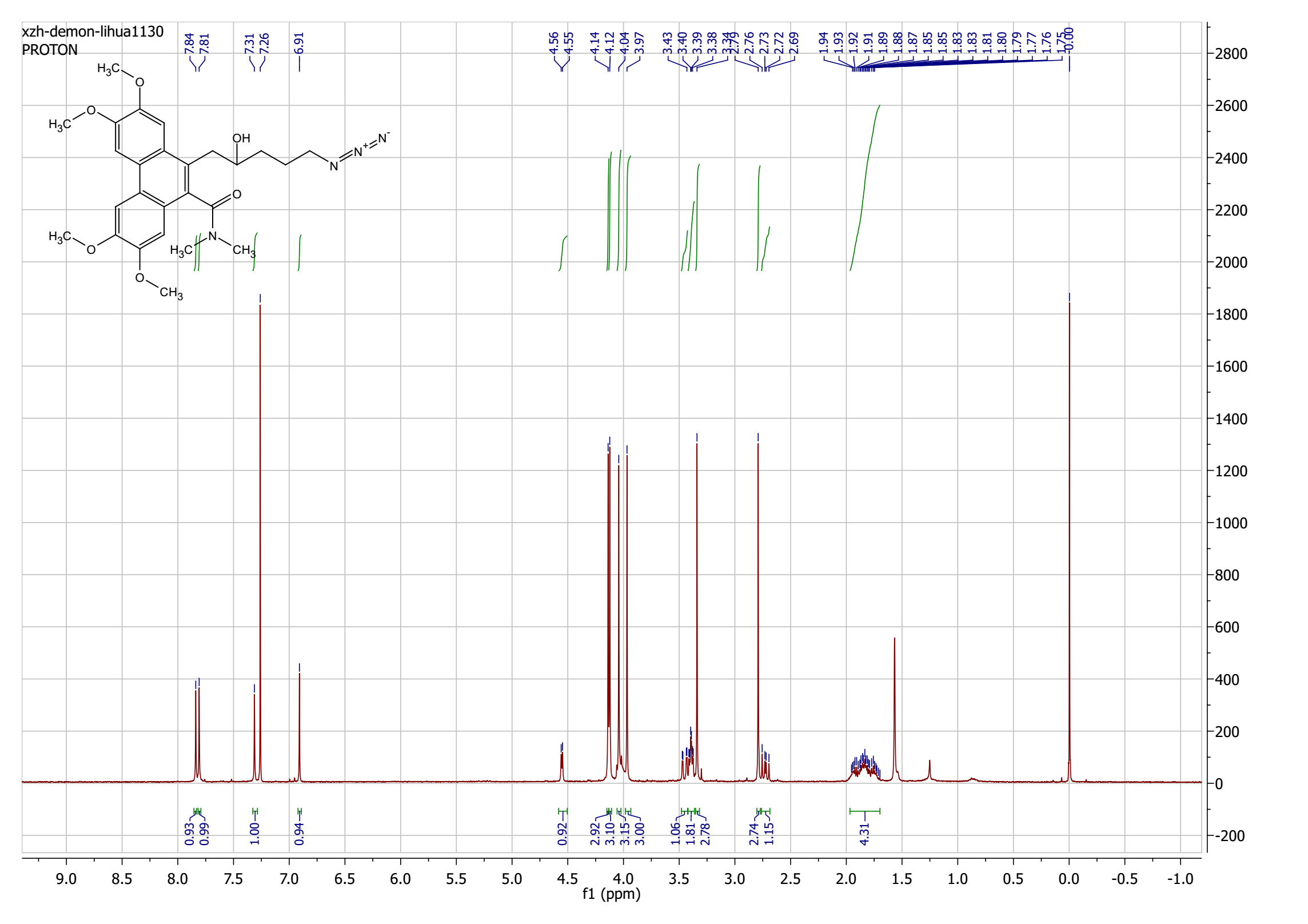


^1^H NMR spectrum of compound **13**


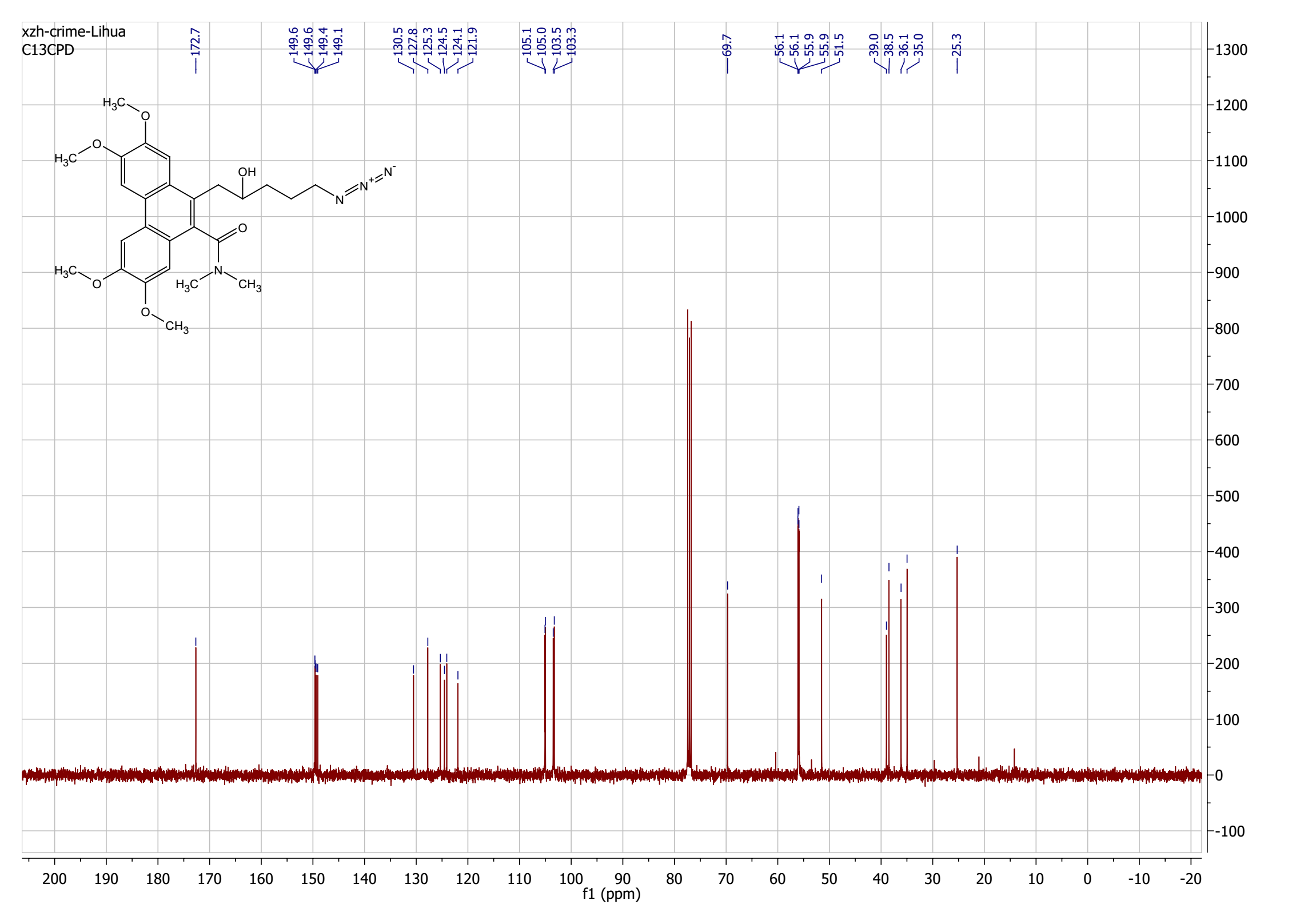


^13^C NMR spectrum of compound **13**


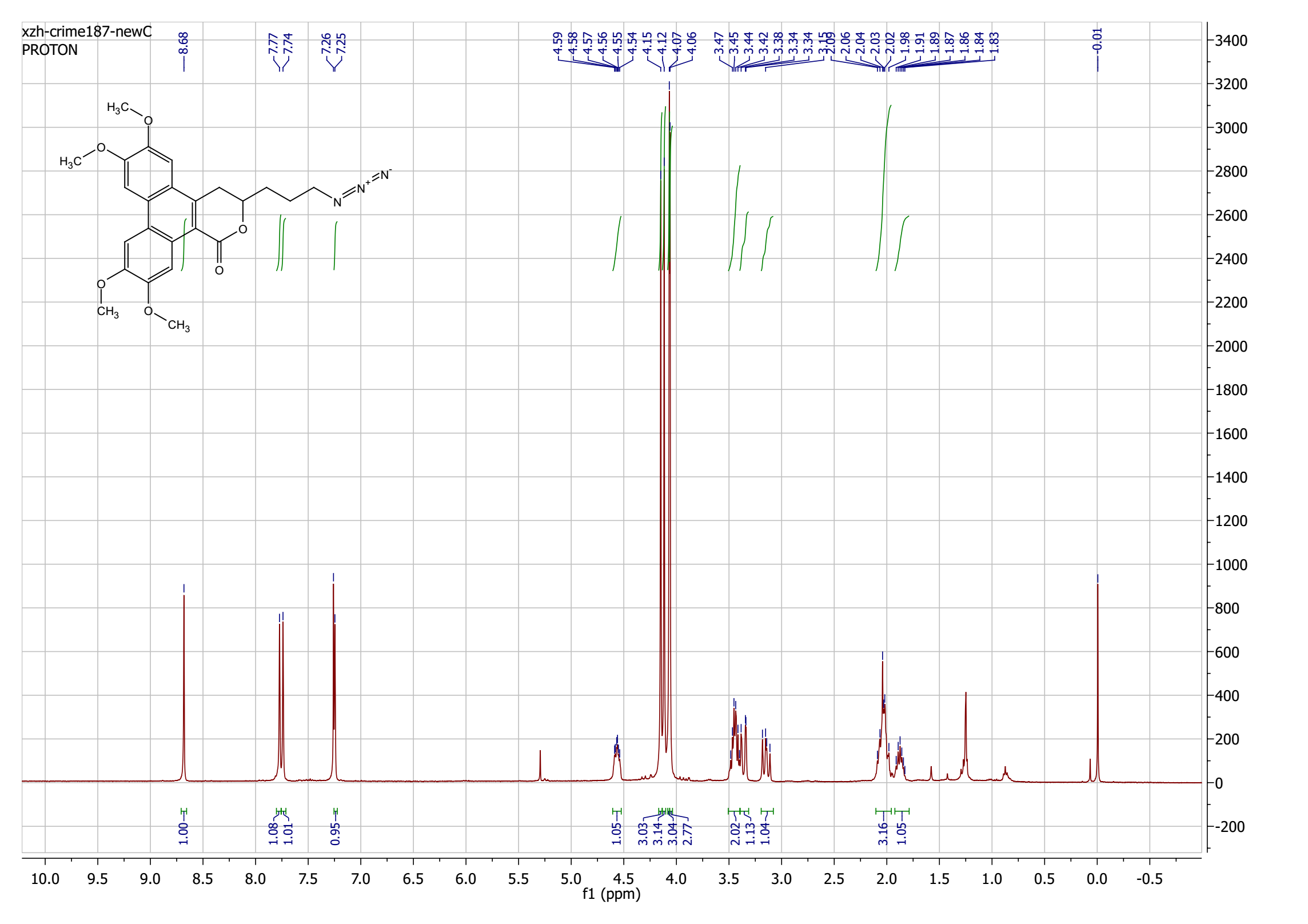


^1^H NMR spectrum of compound **4**


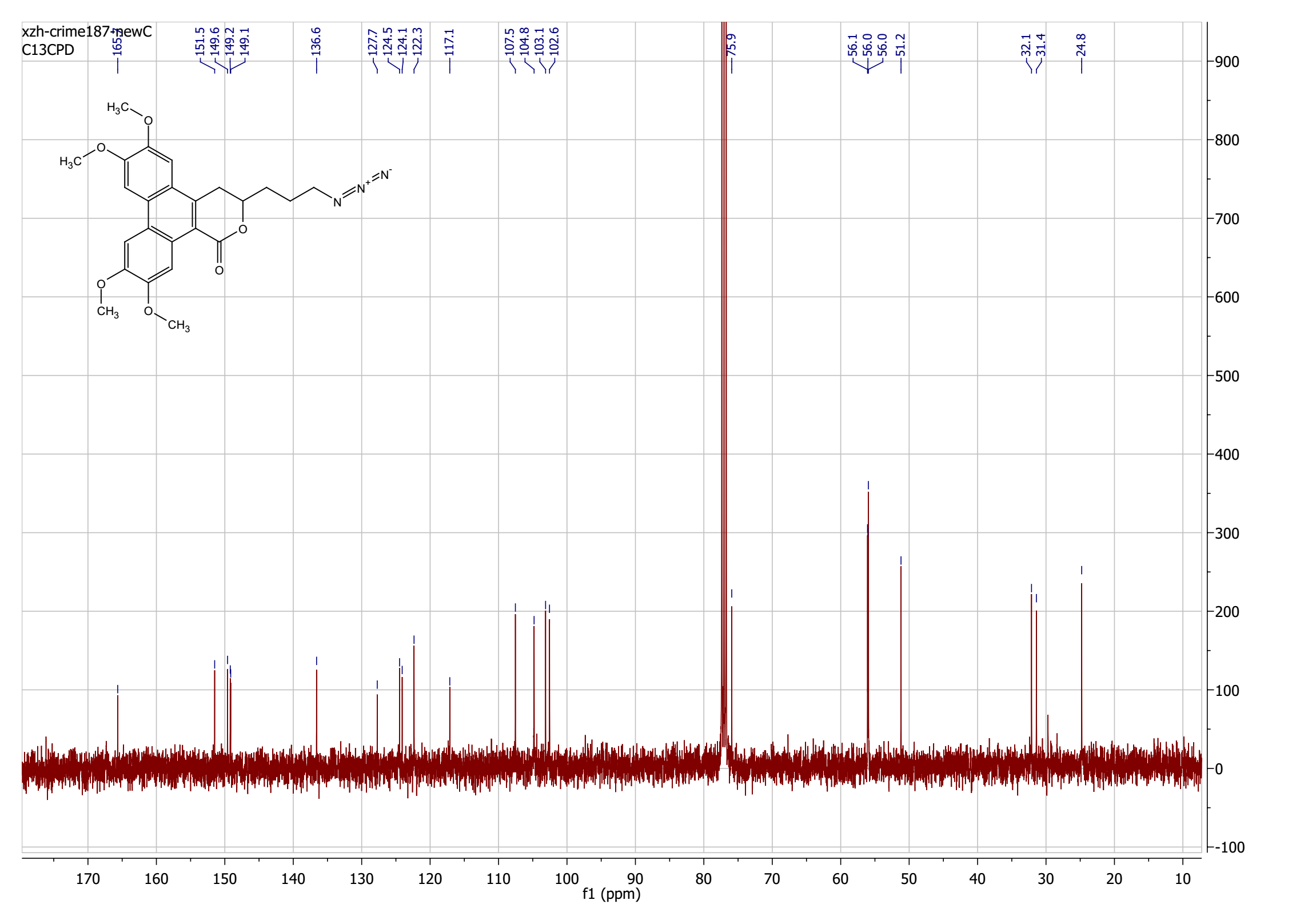


^13^C NMR spectrum of compound **4**


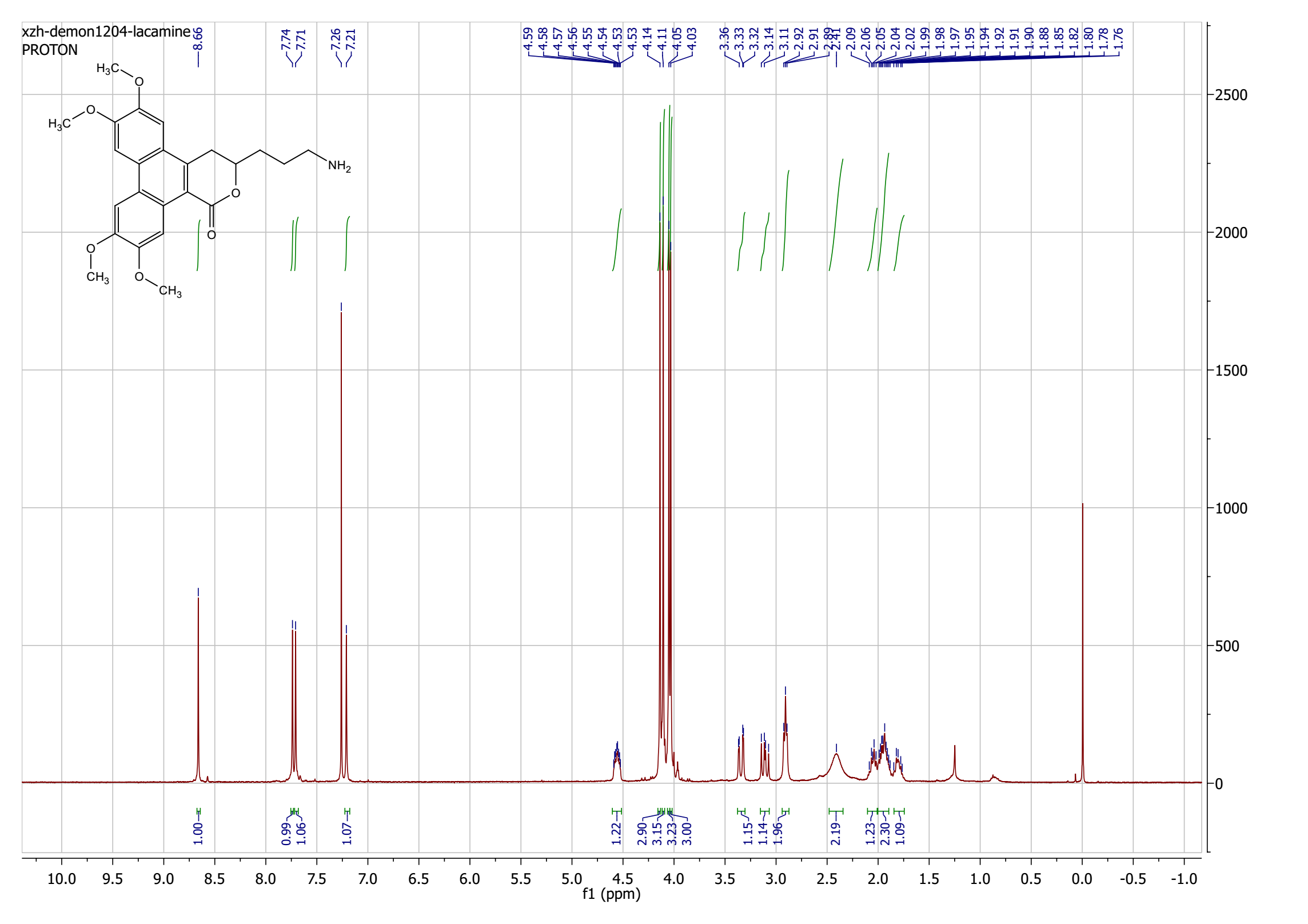


^1^H NMR spectrum of compound **14**


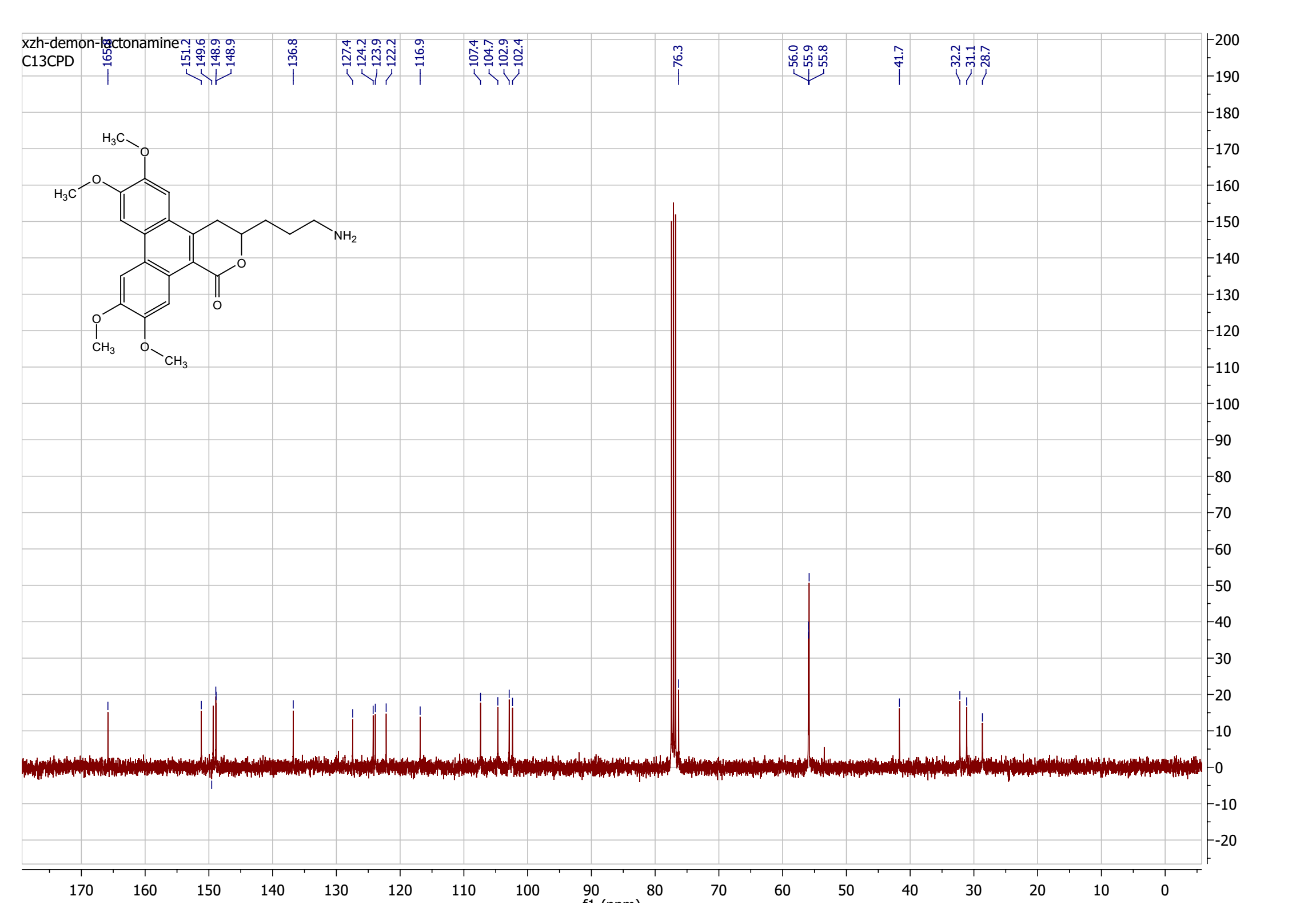


^13^C NMR spectrum of compound **14**


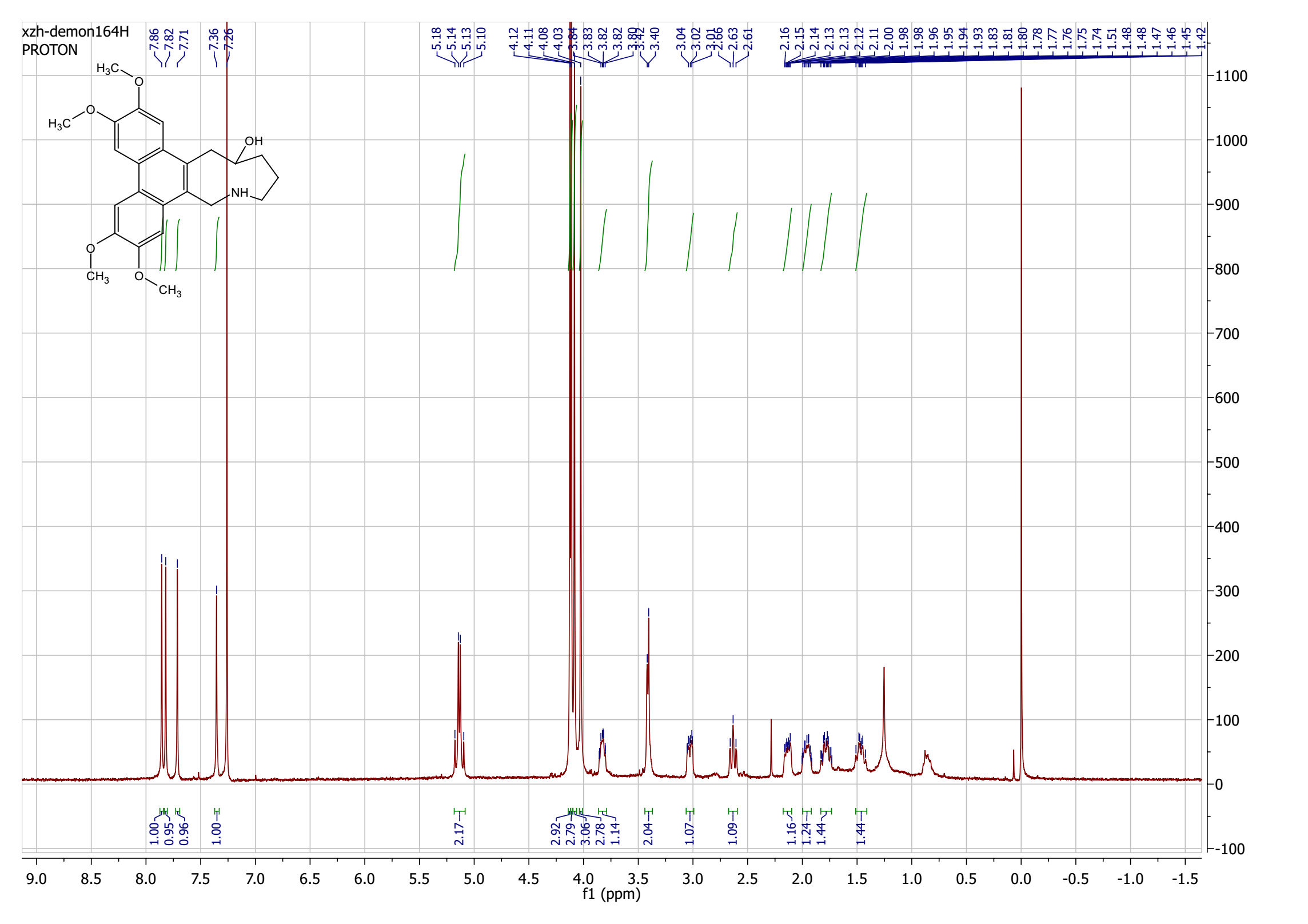
^1^H NMR spectrum of compound **3**


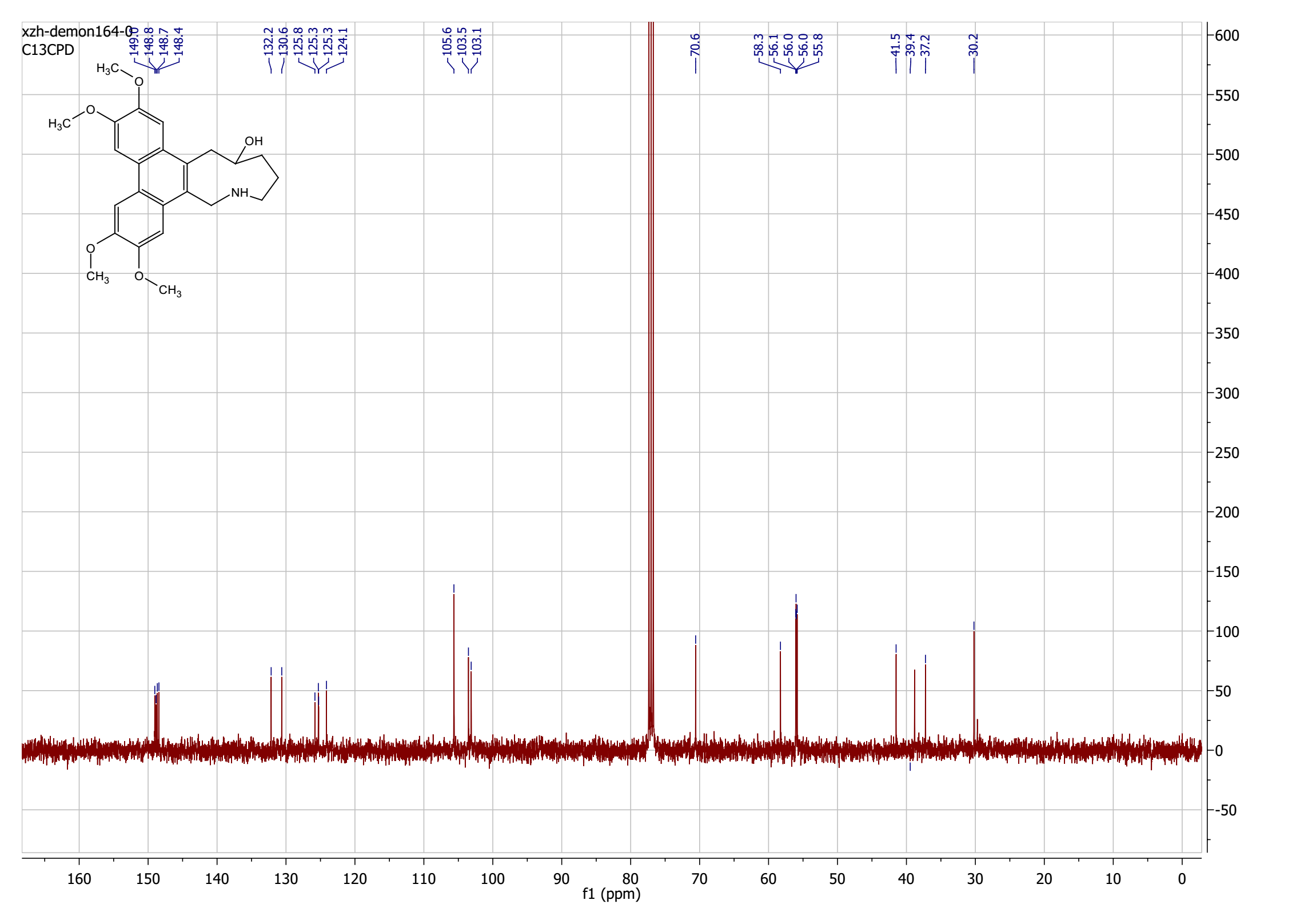


^13^C NMR spectrum of compound **3**


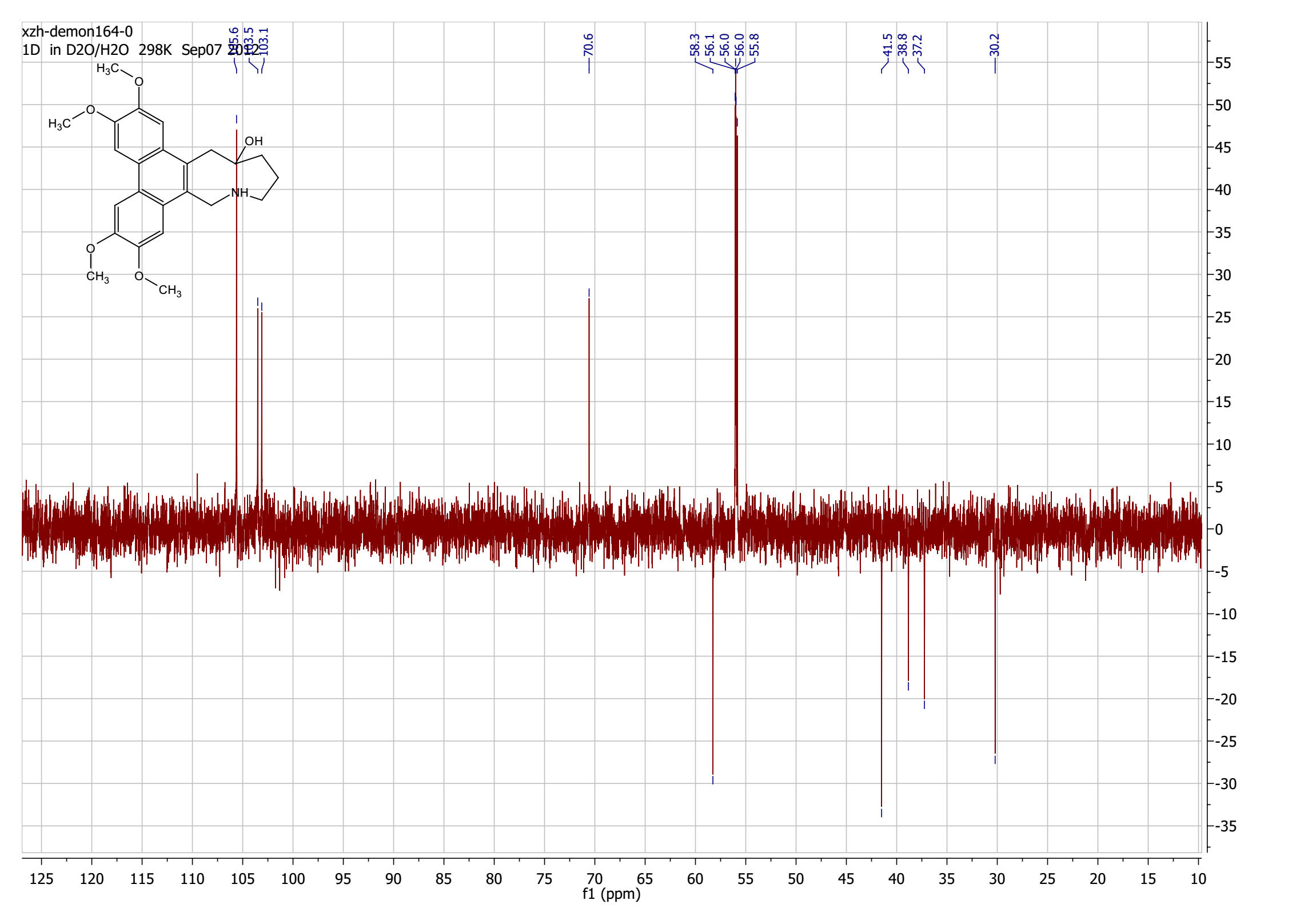


DEPT-135 ^13^C NMR spectrum of compound **3**


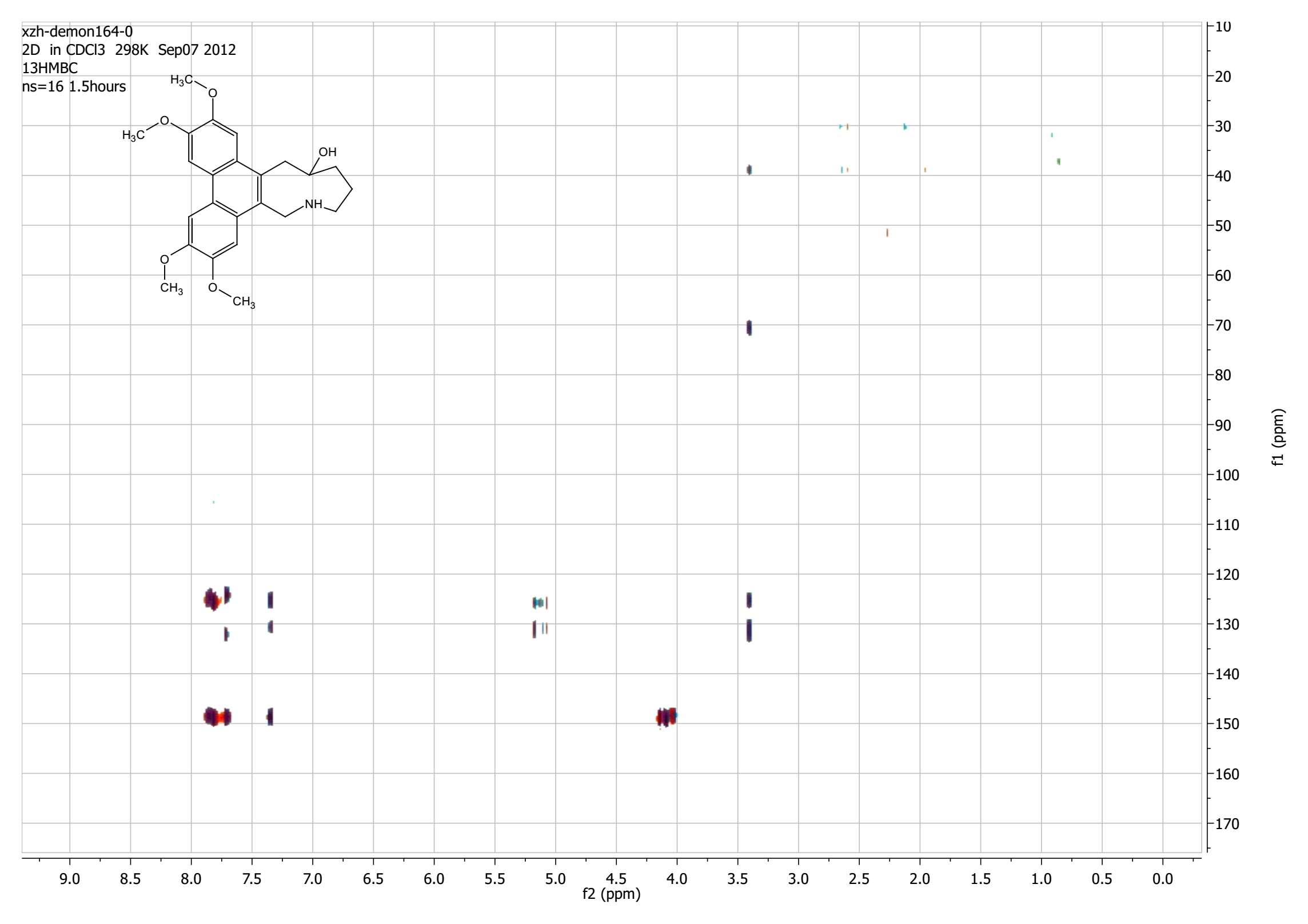


HMBC spectrum of compound **3**


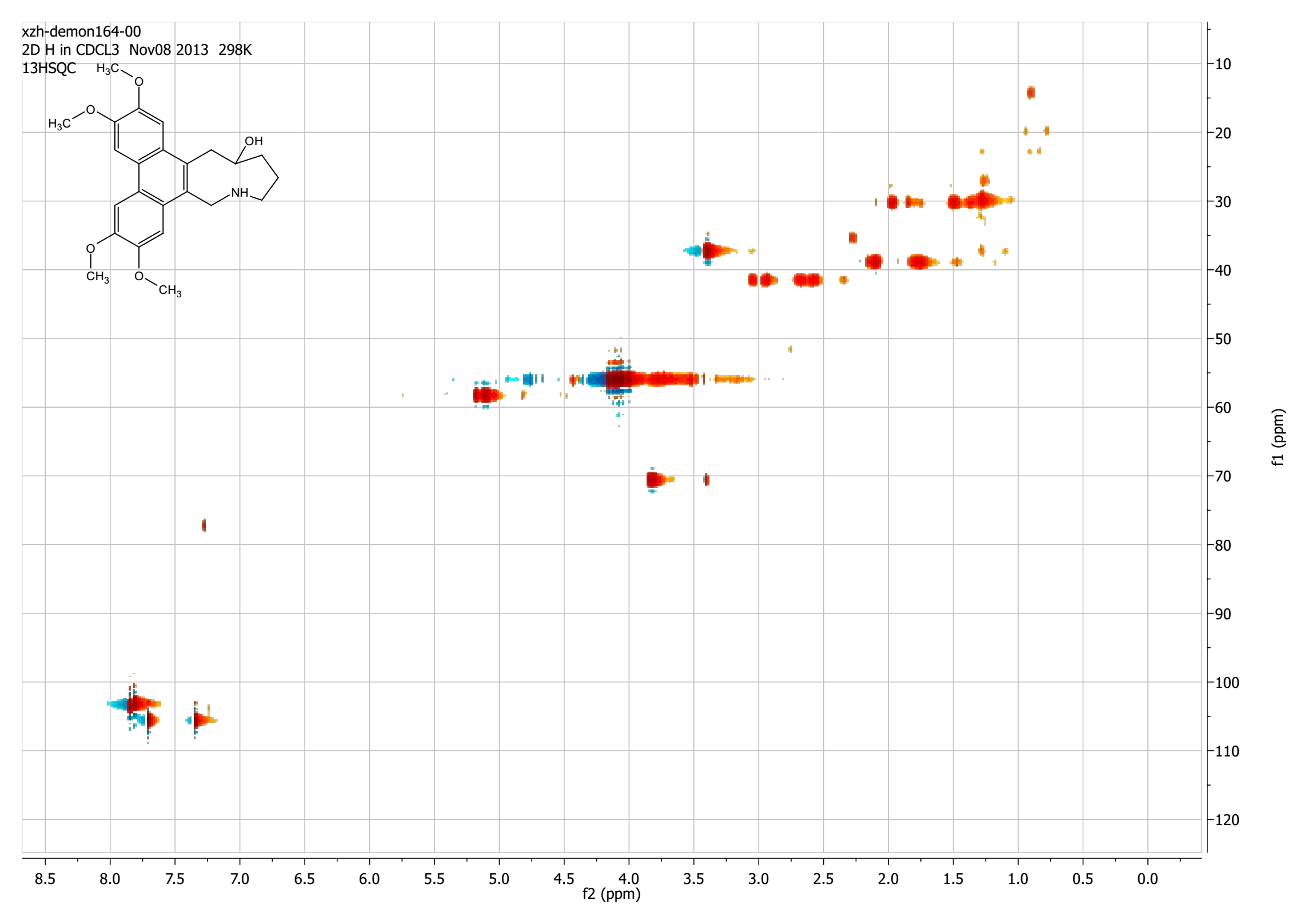


HSQC spectrum of compound **3**


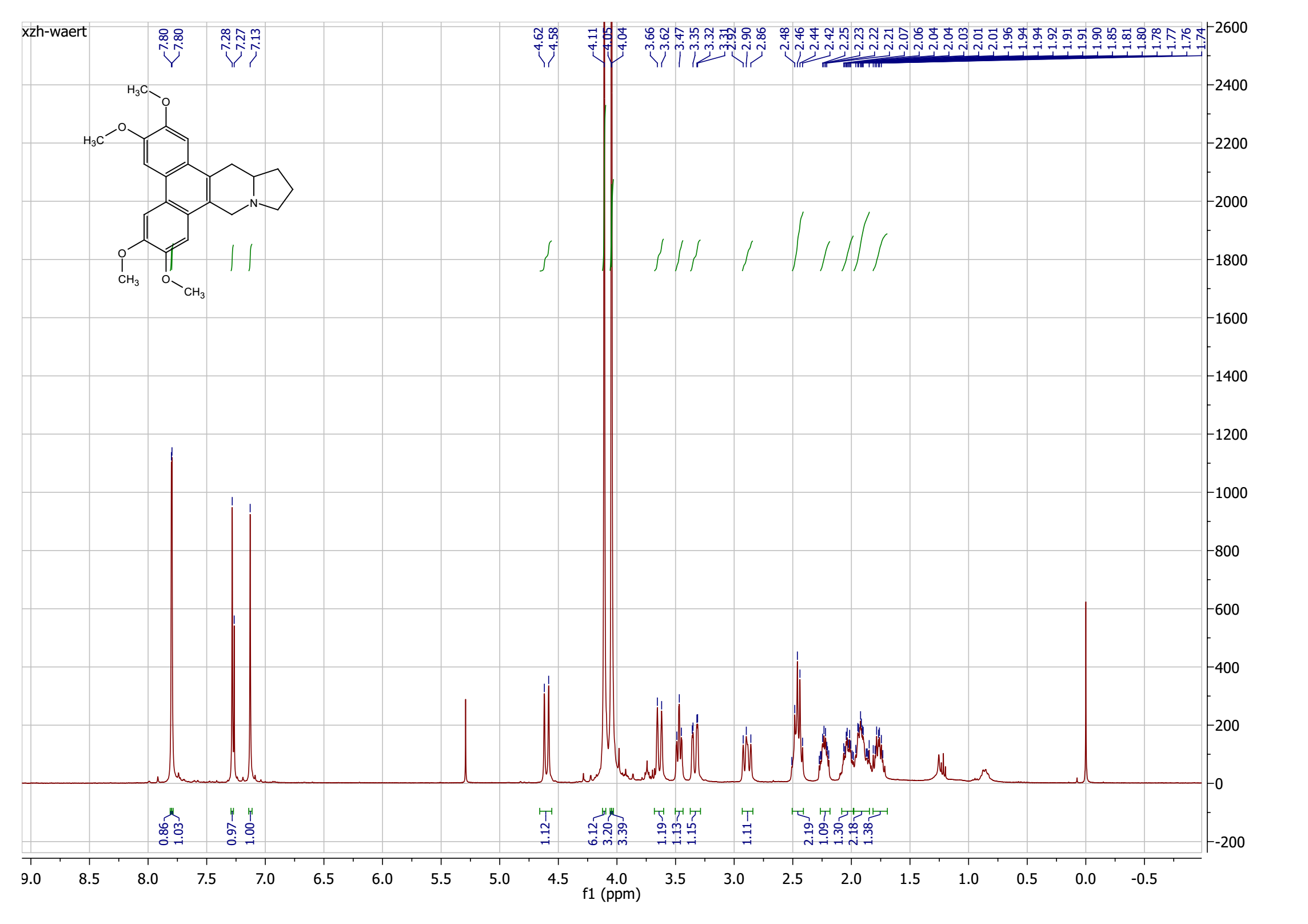


^1^H NMR spectrum of **tylophorine**


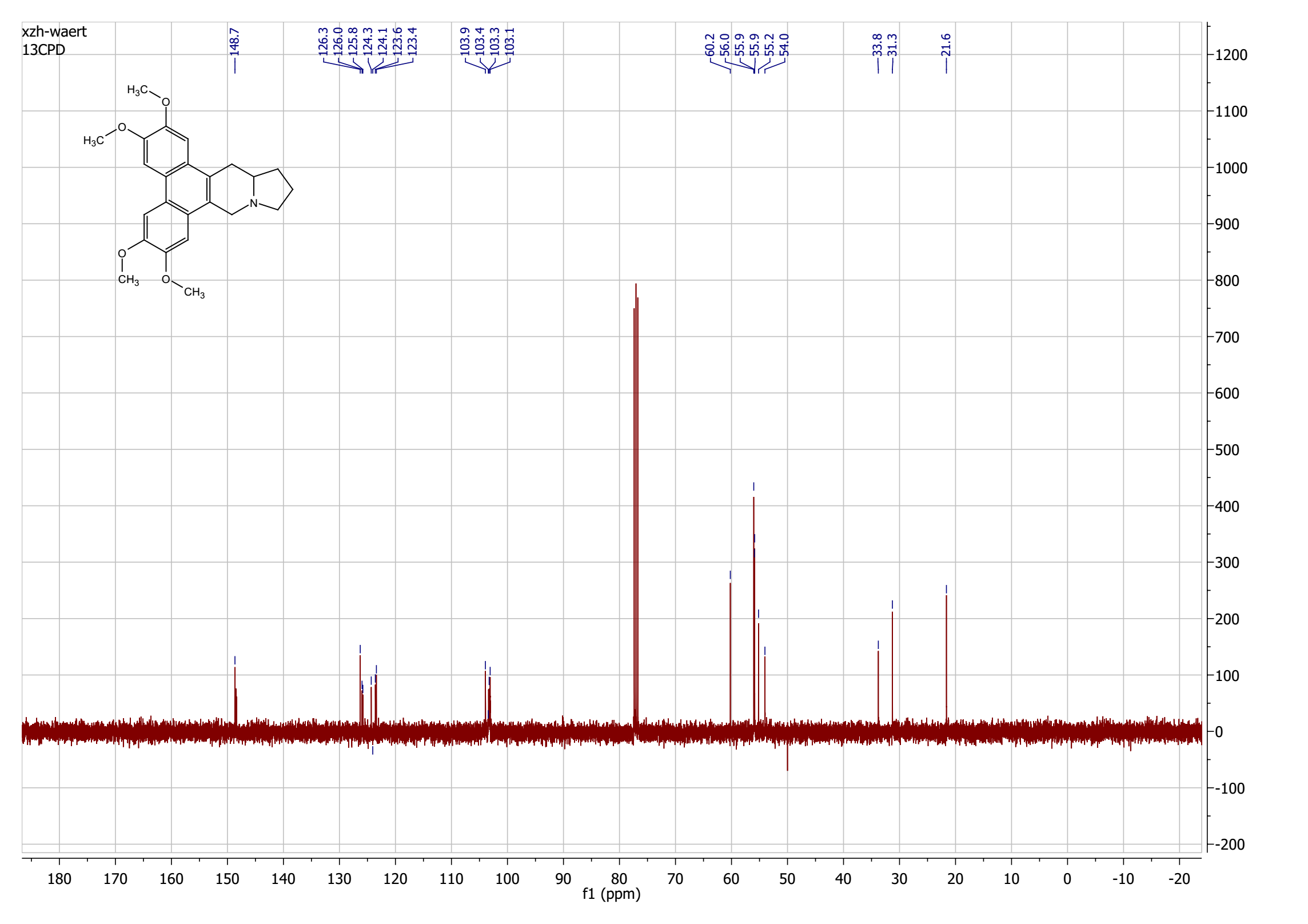


^13^C NMR spectrum of **tylophorine**


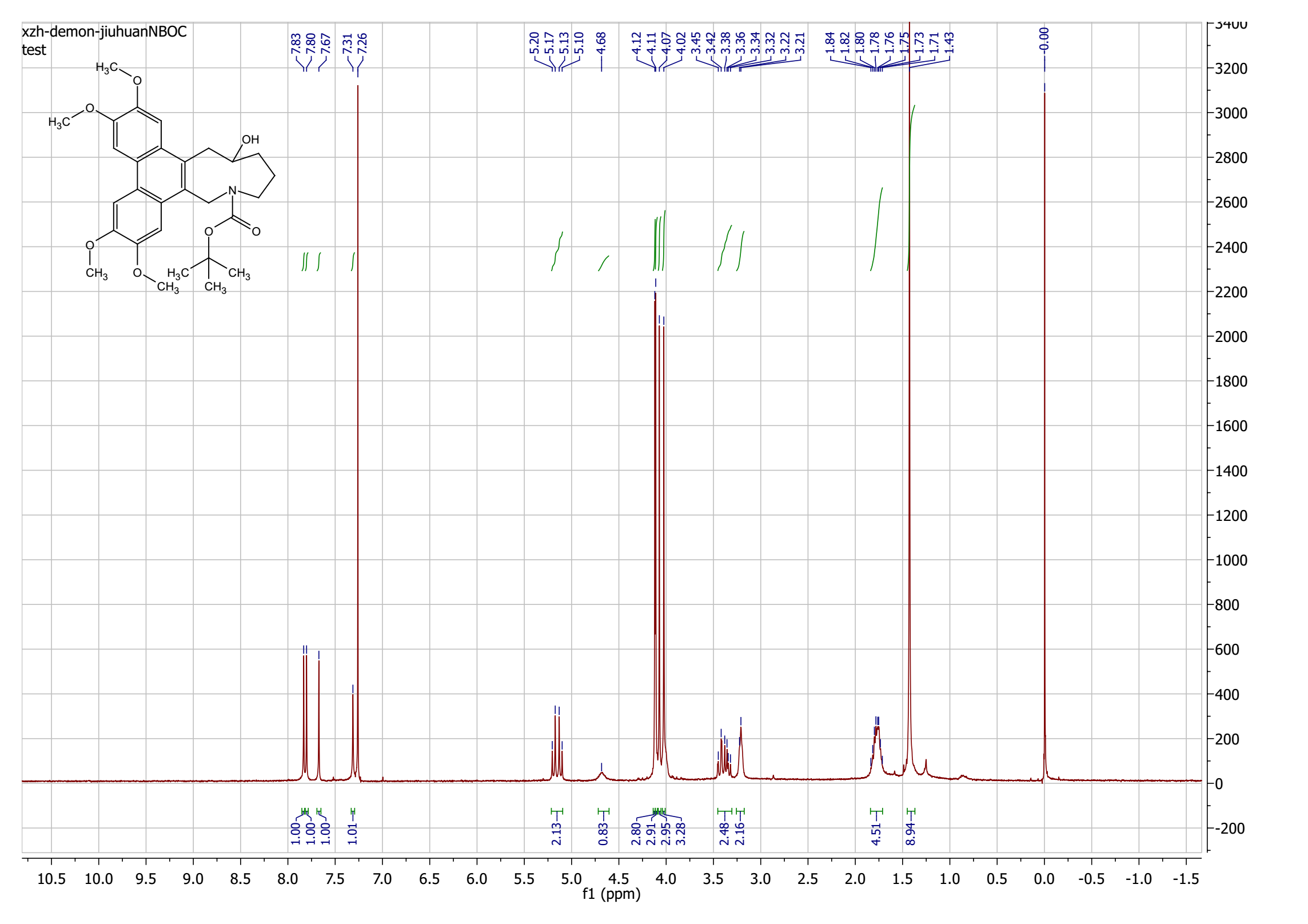


^1^H NMR spectrum of compound **16**


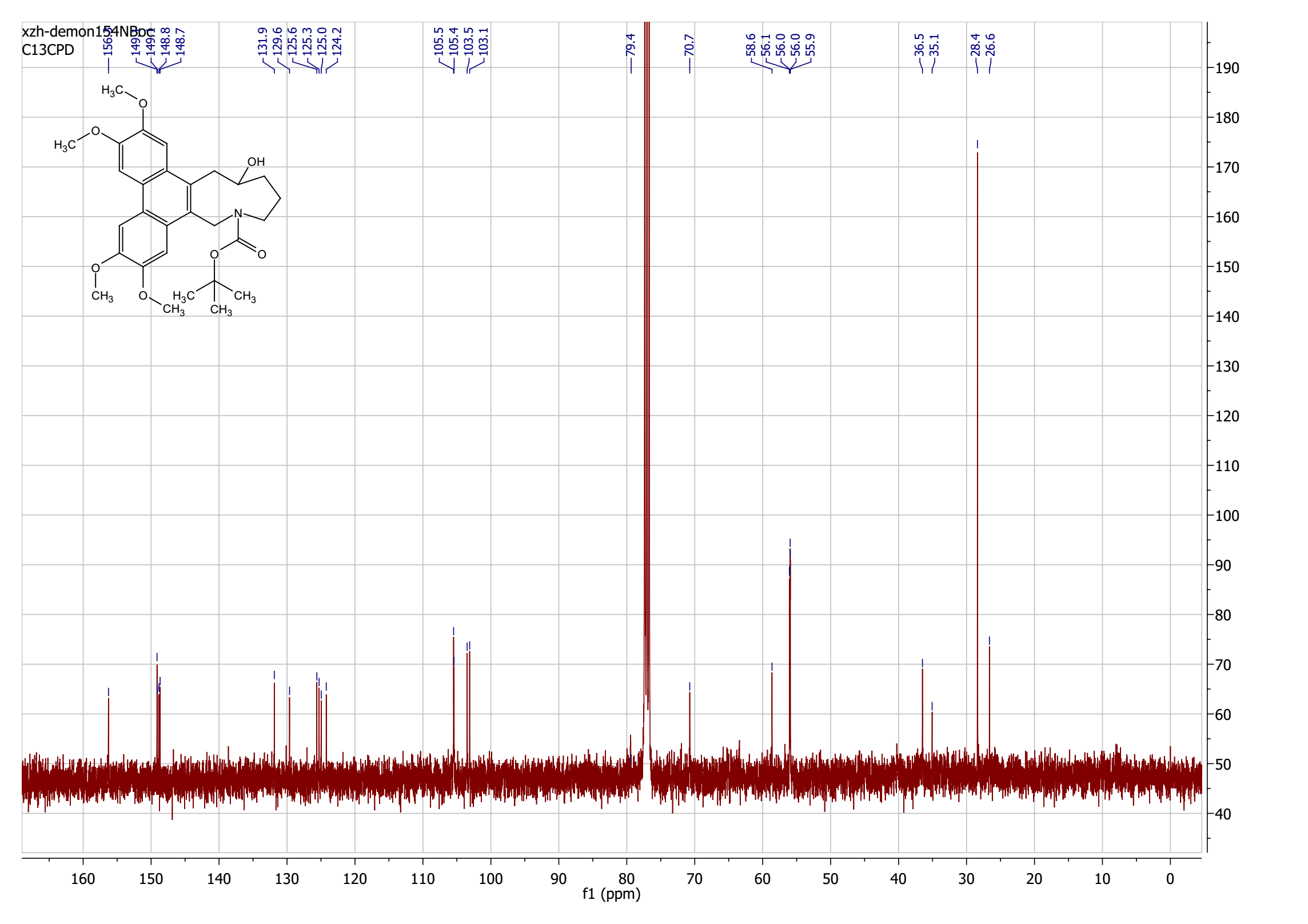


^13^C NMR spectrum of compound **16**


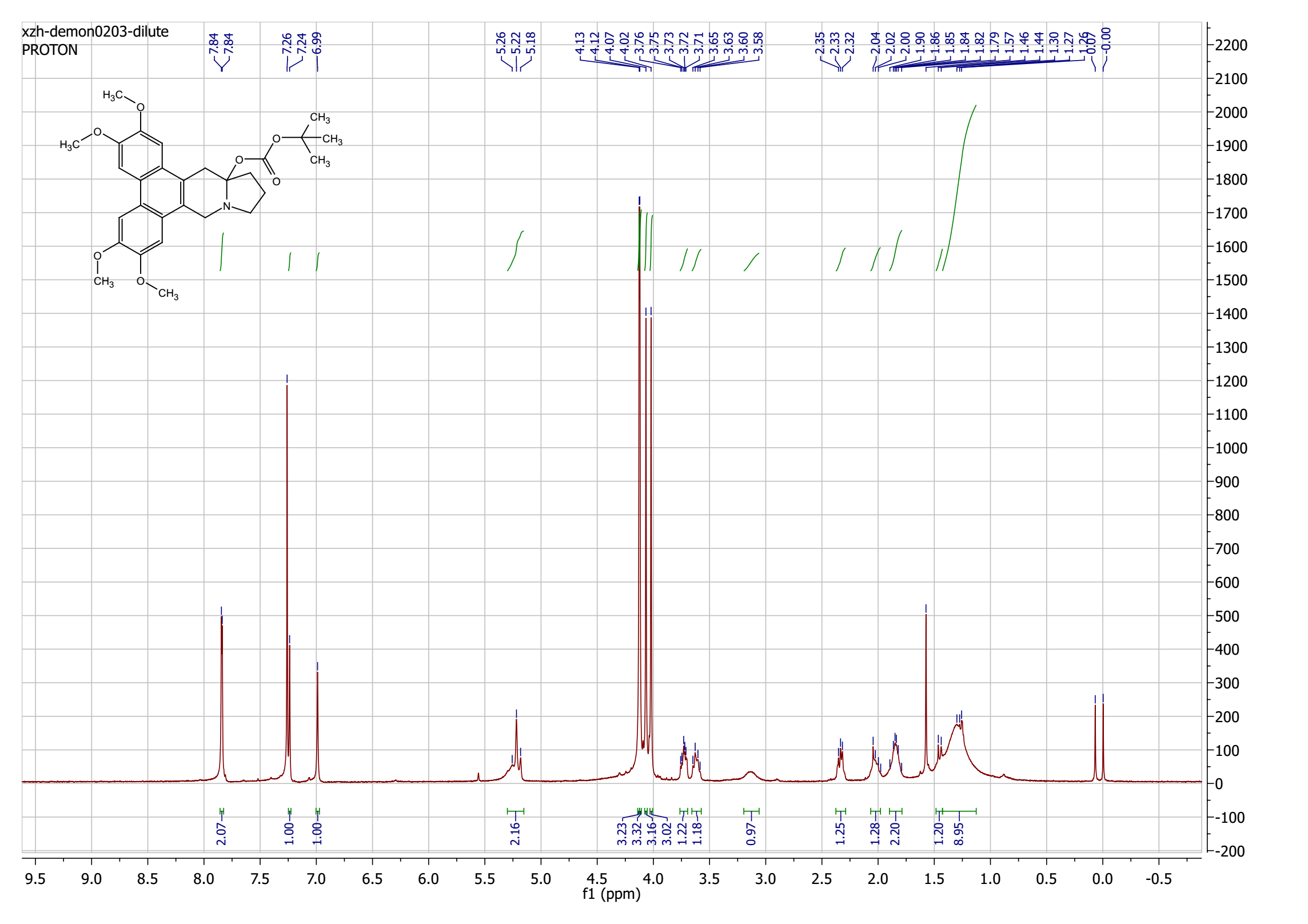


^1^H NMR spectrum of compound **18**


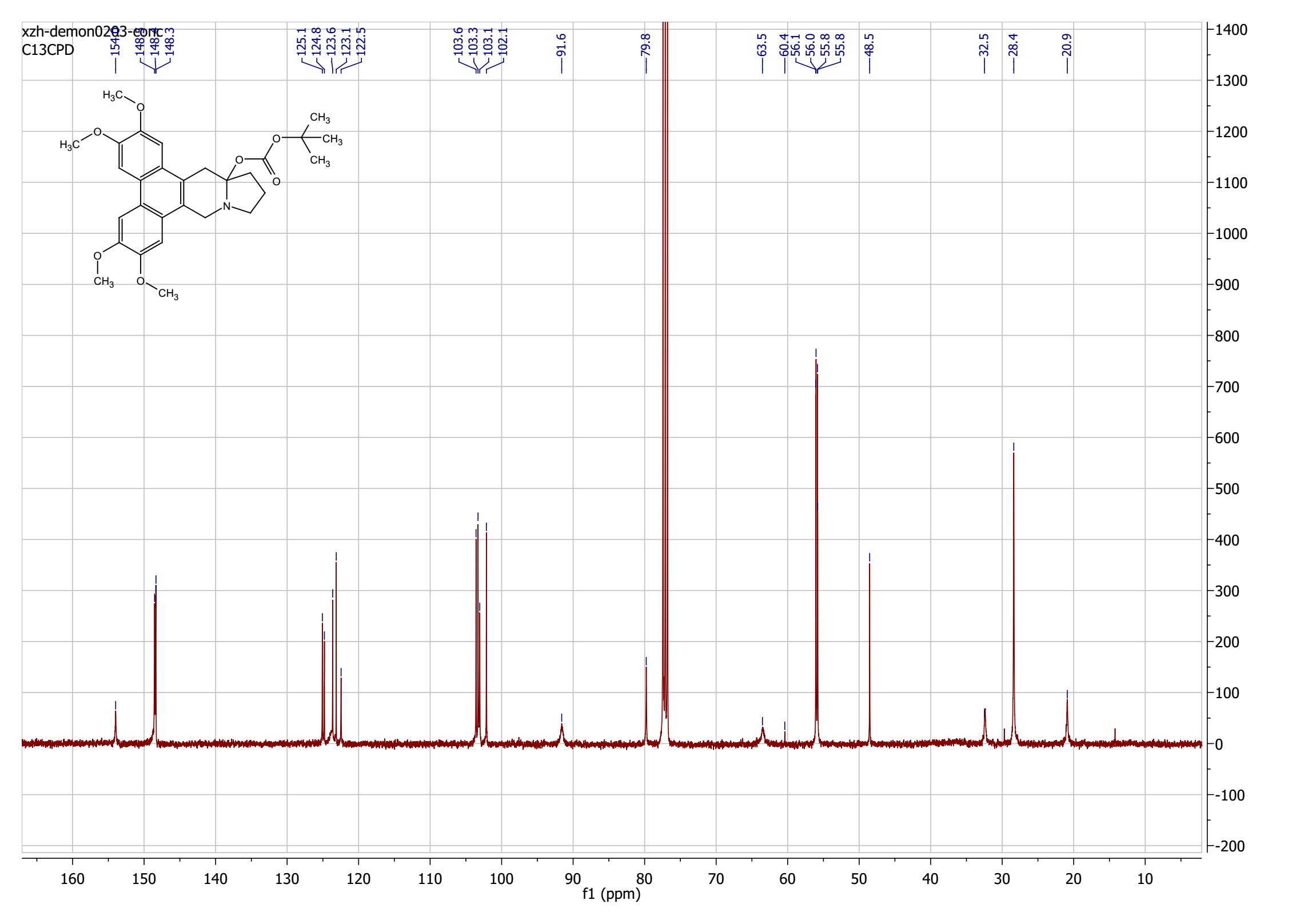


^13^C NMR spectrum of compound **18**


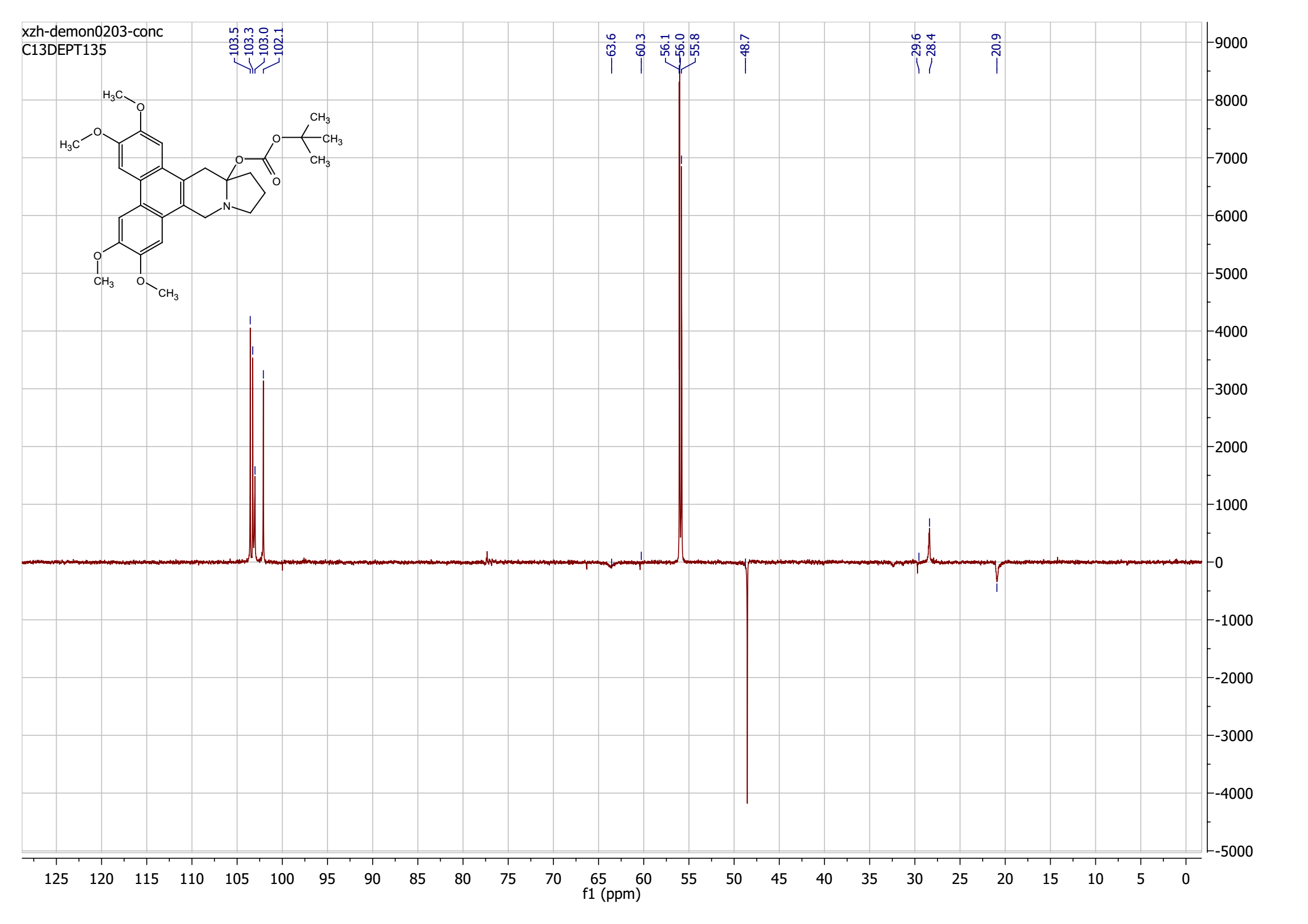


DEPT-135 ^13^C NMR spectrum of compound **18**


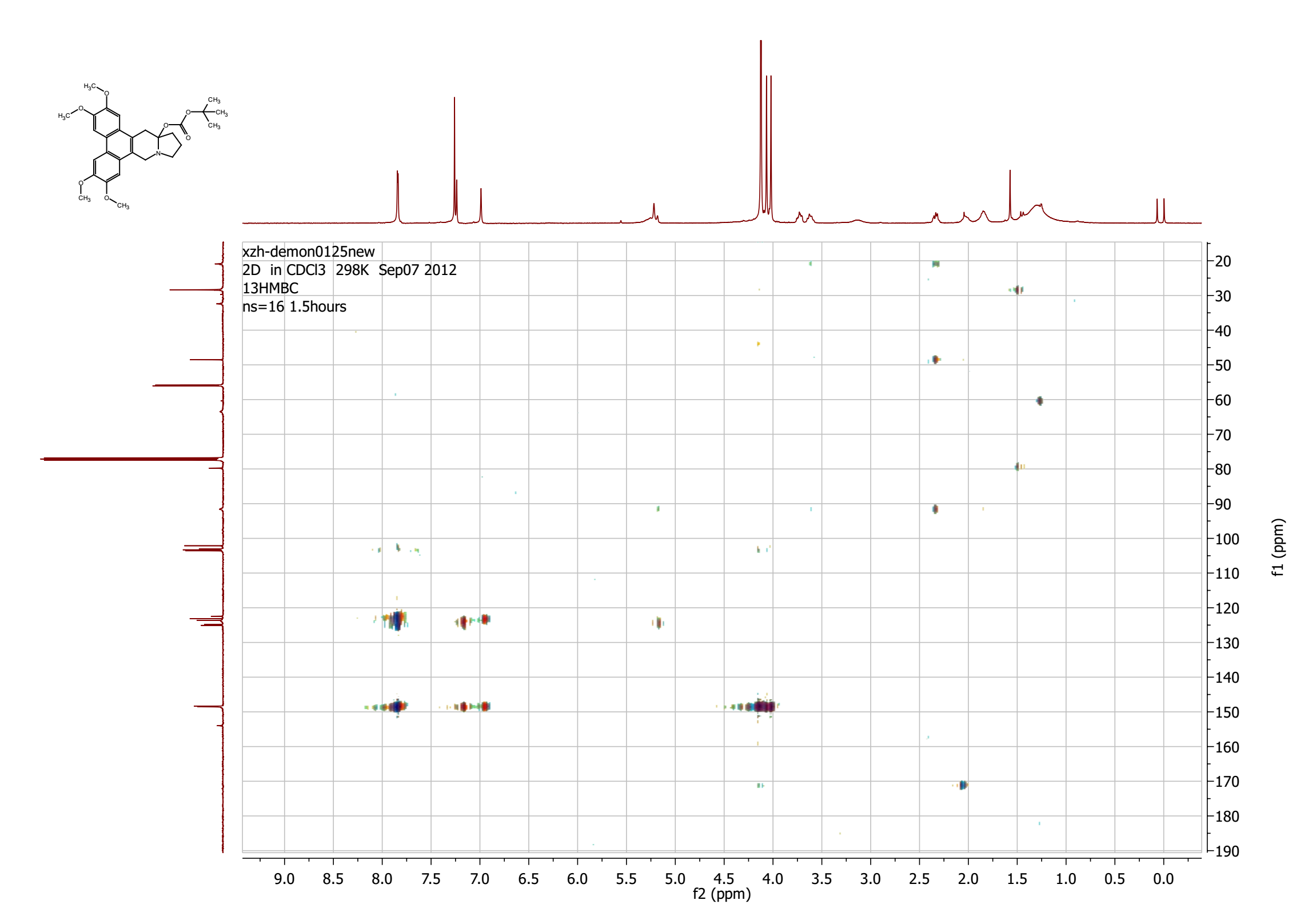


HMBCspectrum of compound **18**


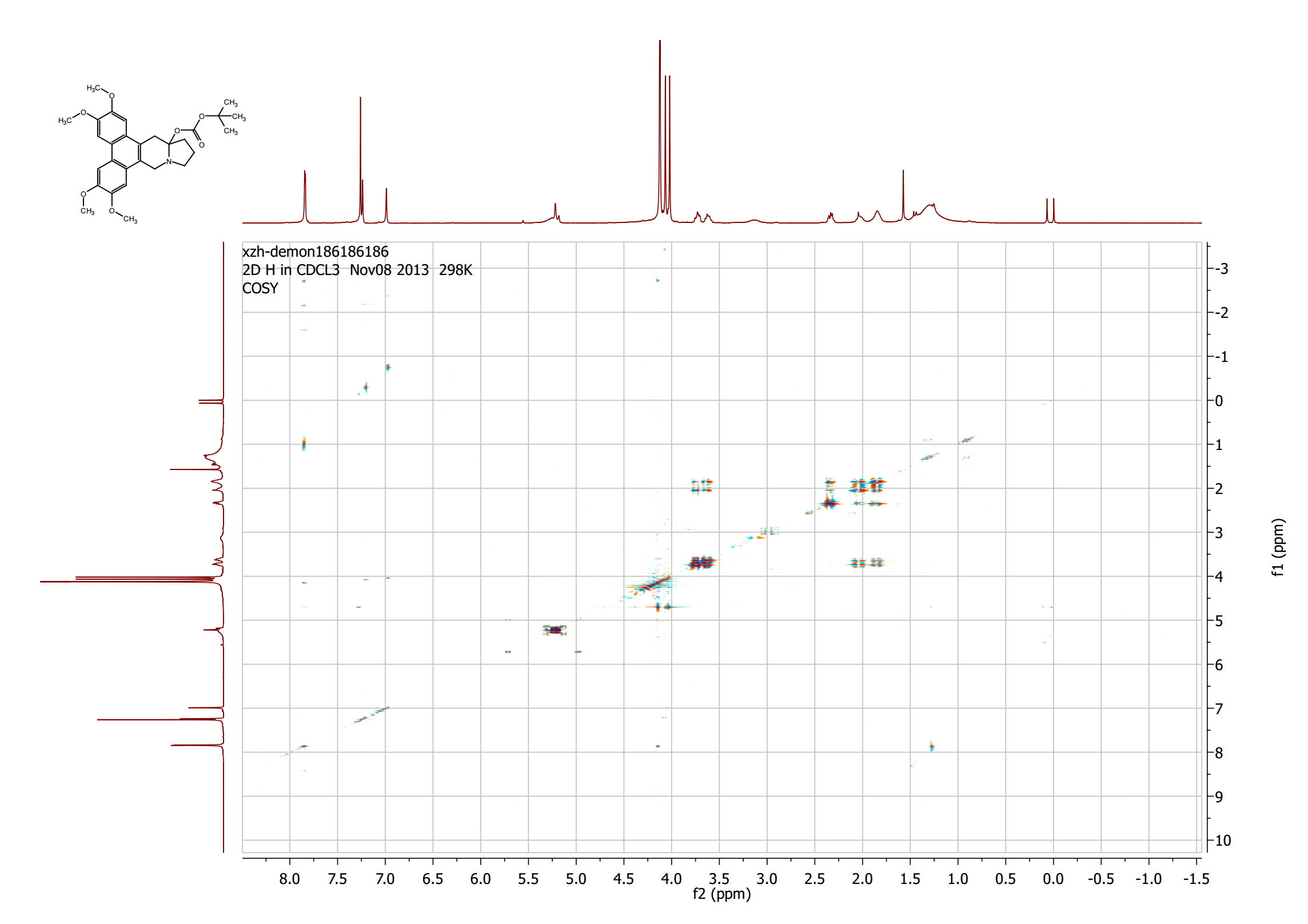


H-H COSYspectrum of compound **18**


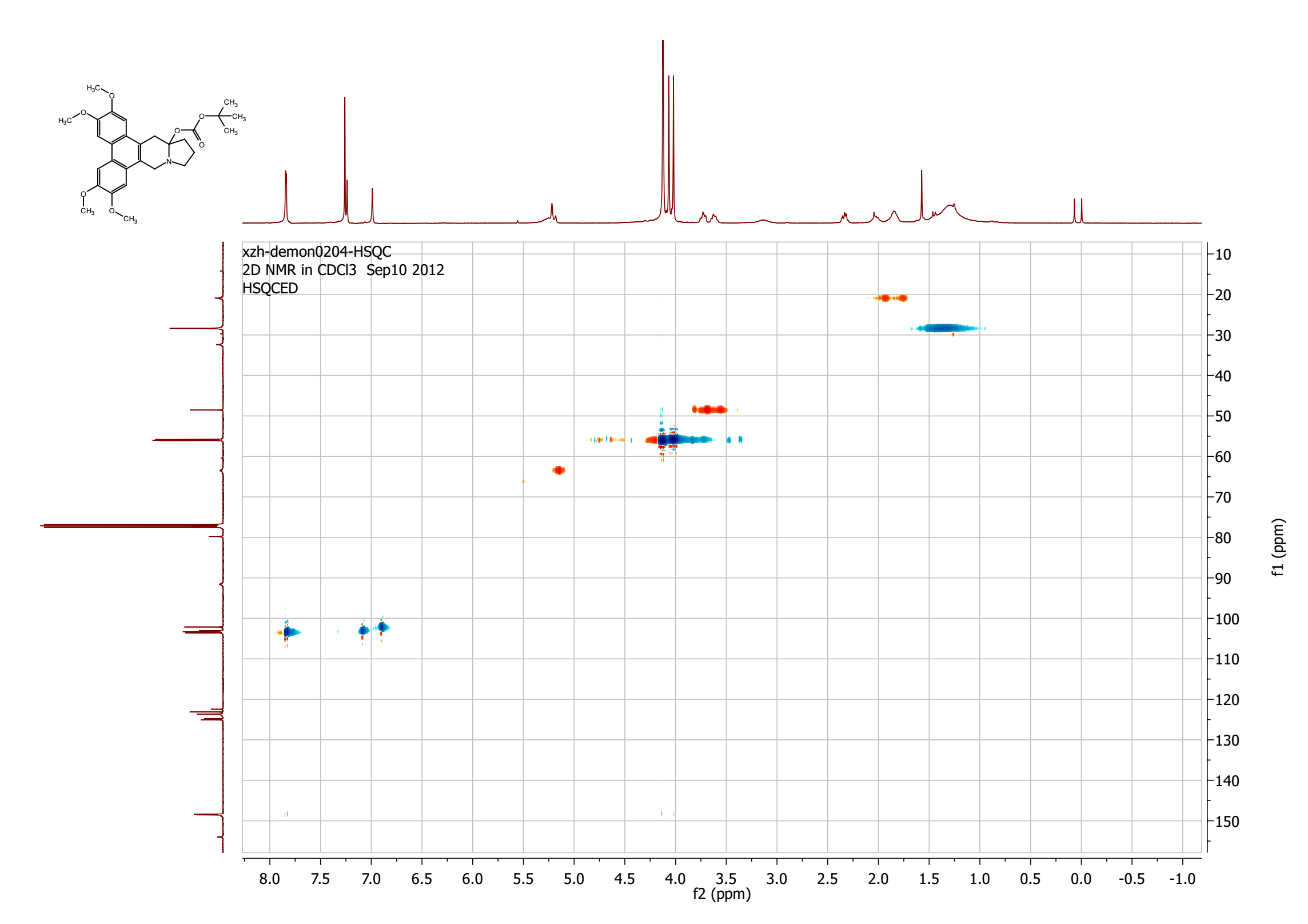


HSQC spectrum of compound **18**


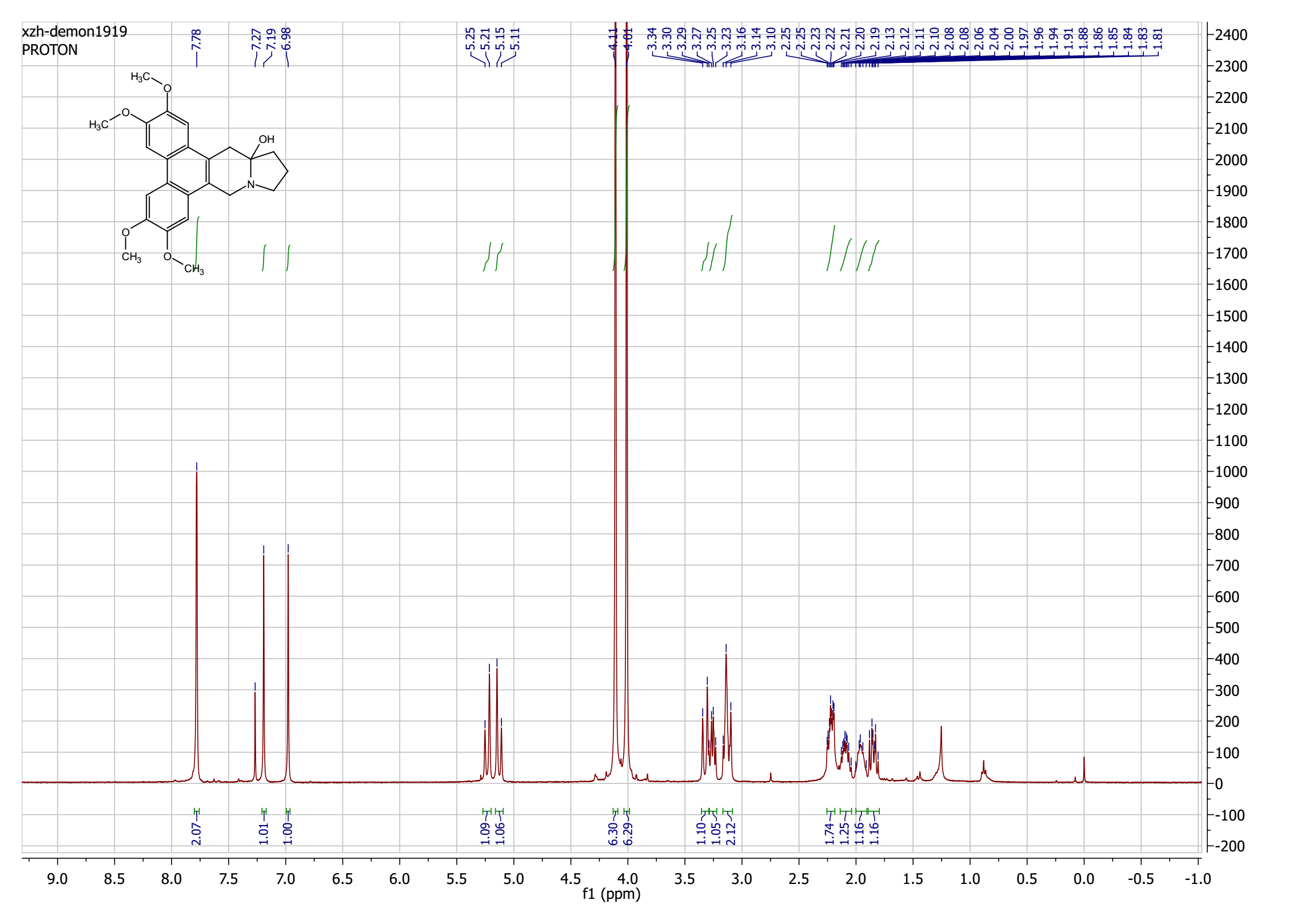


^1^H NMR spectrum of **13a-hydroxytylophorine 1**


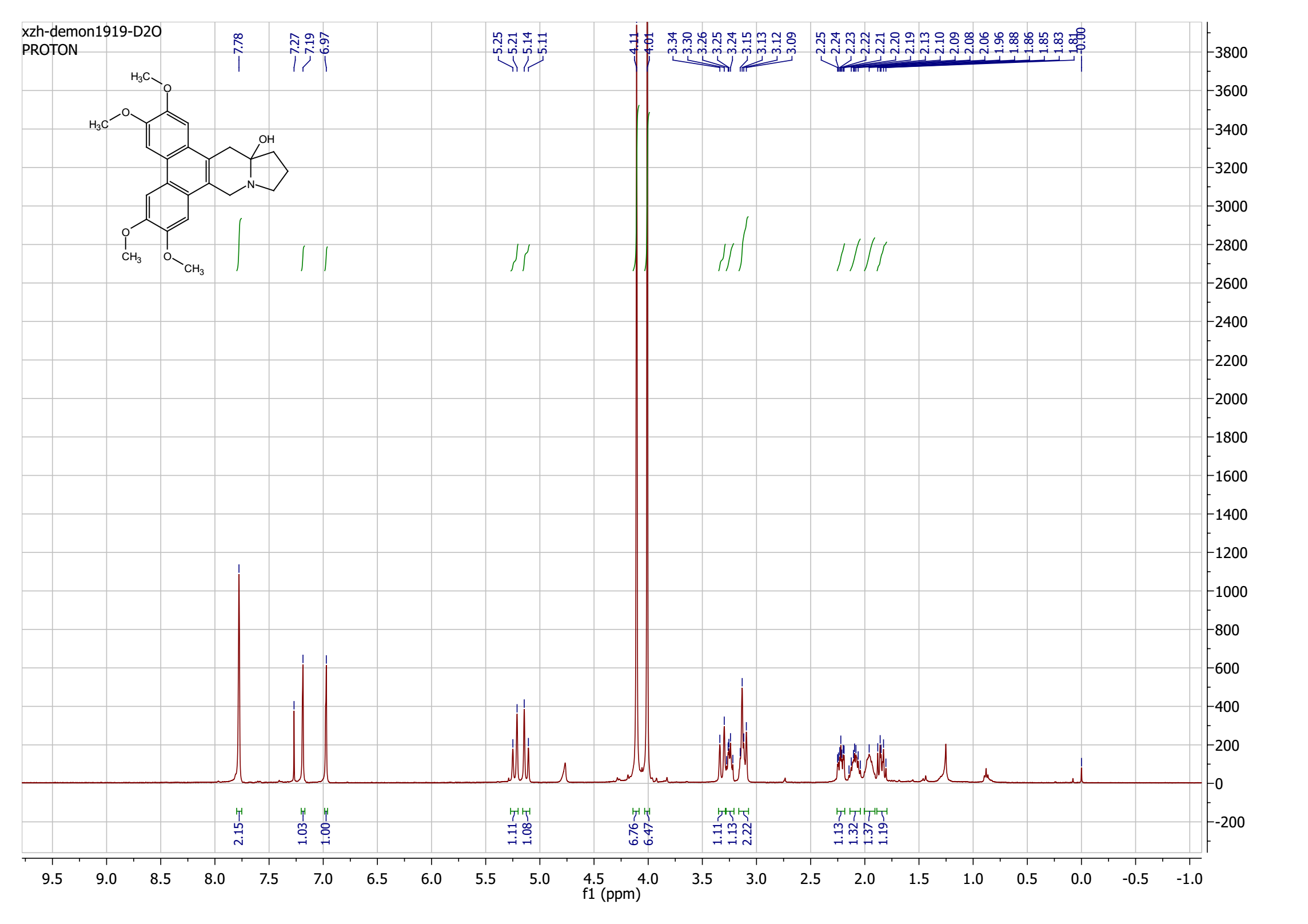


^1^H NMR spectrum of **13a-hydroxytylophorine 1** with D_2_O exchange


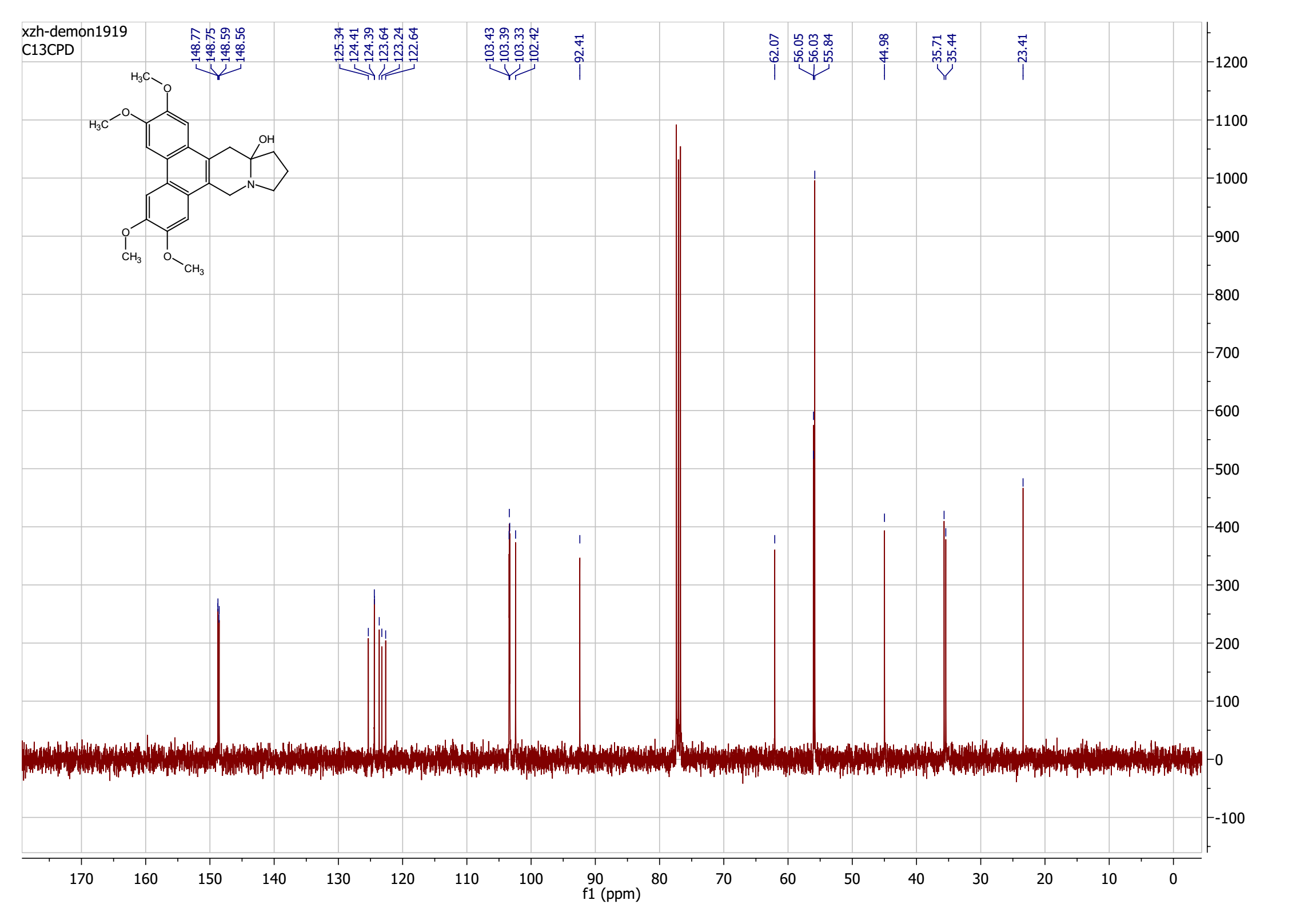


^13^C NMR spectrum of **13a-hydroxytylophorine 1**


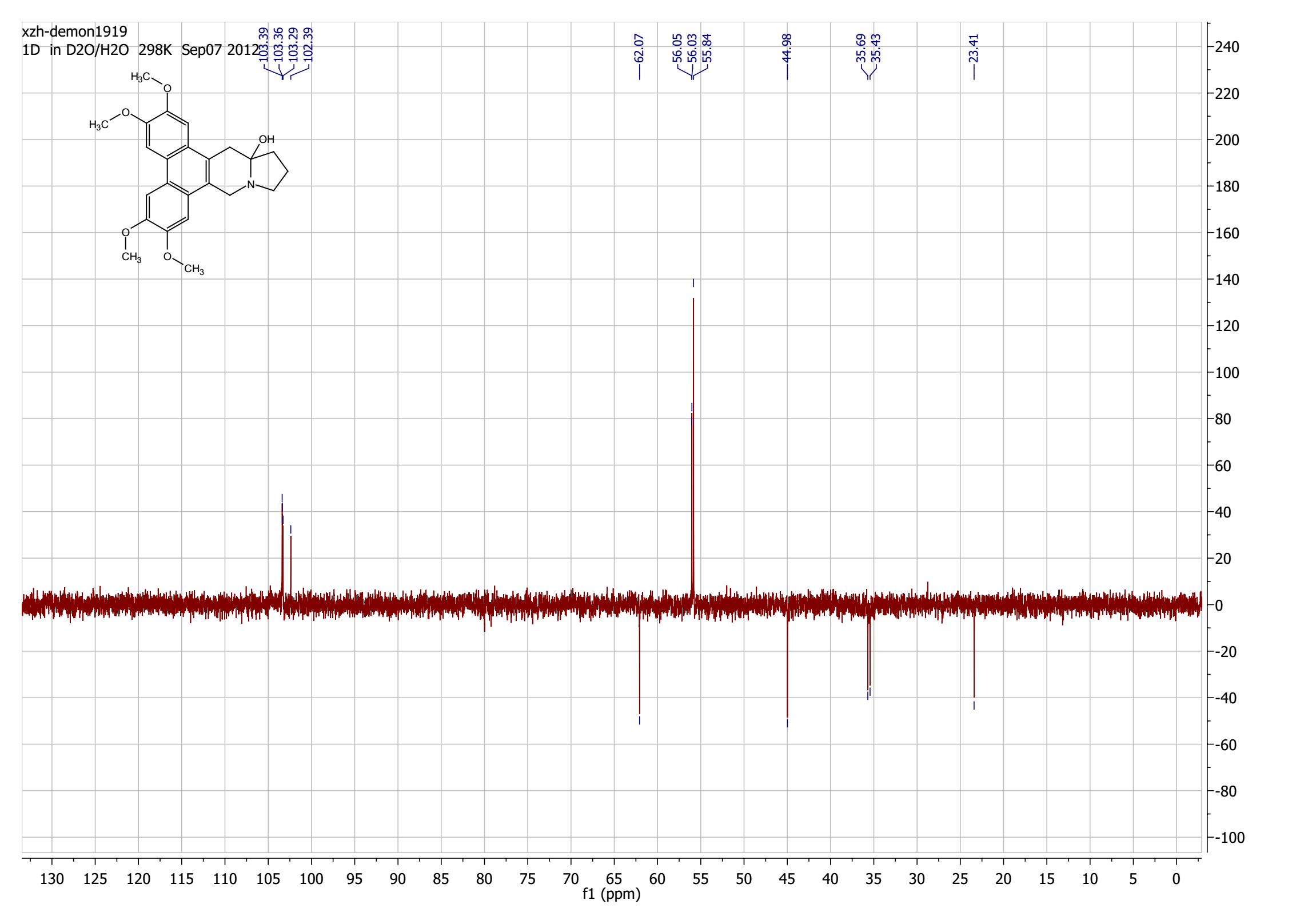


DEPT-135 ^13^C NMR spectrum of **13a-hydroxytylophorine 1**


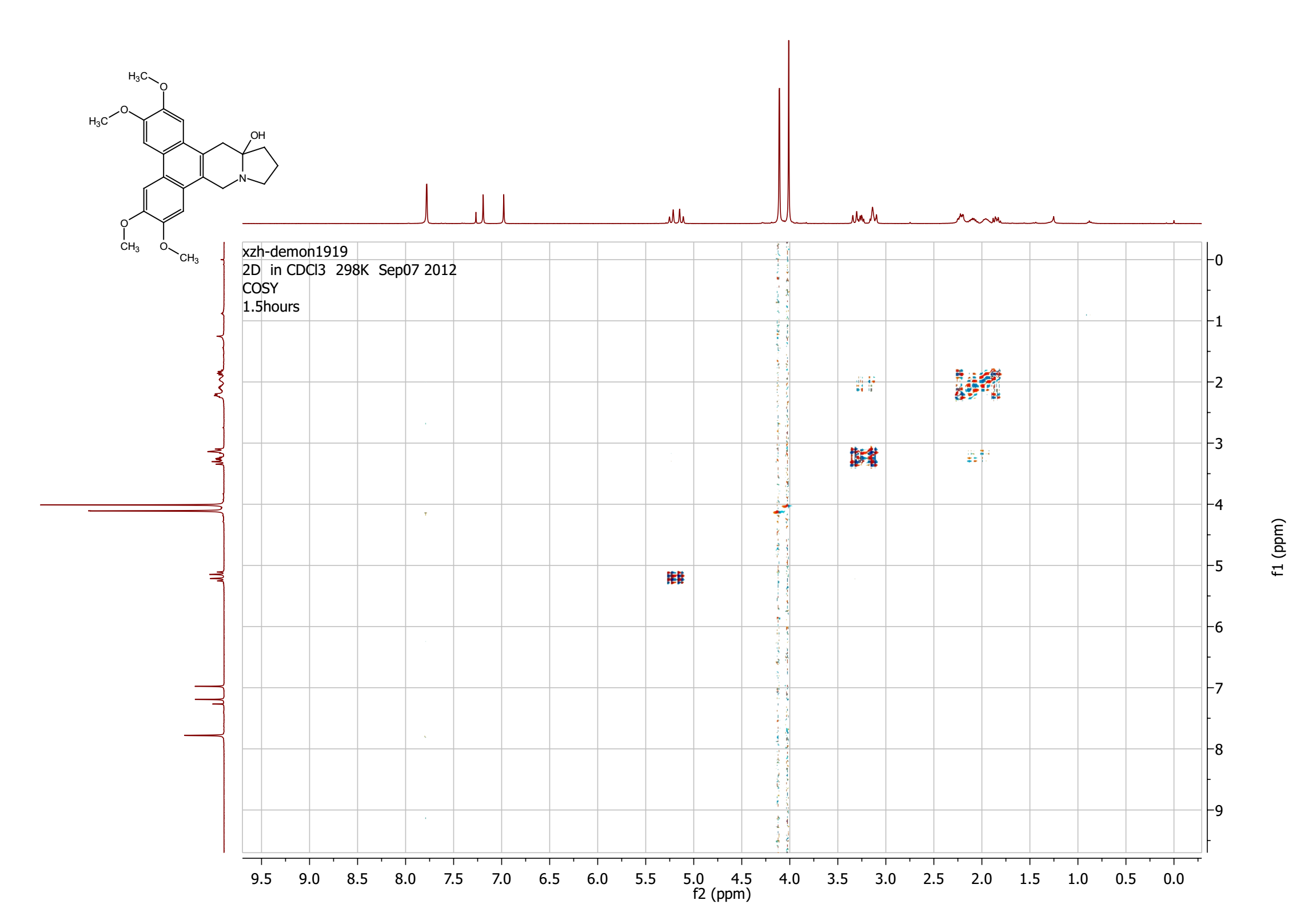


H-H COSY spectrum of **13a-hydroxytylophorine 1**


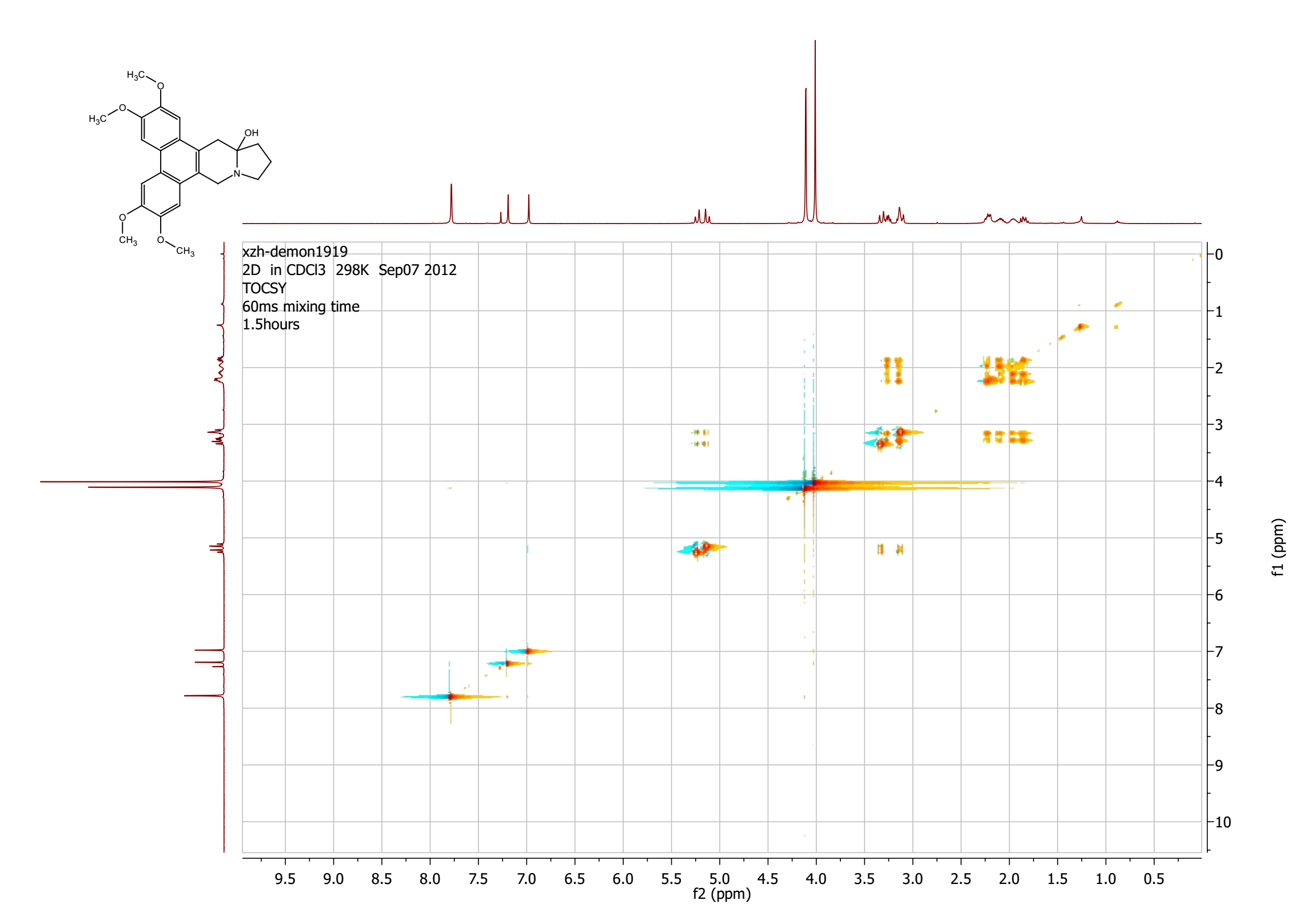


TOCSYspectrum of **13a-hydroxytylophorine 1**


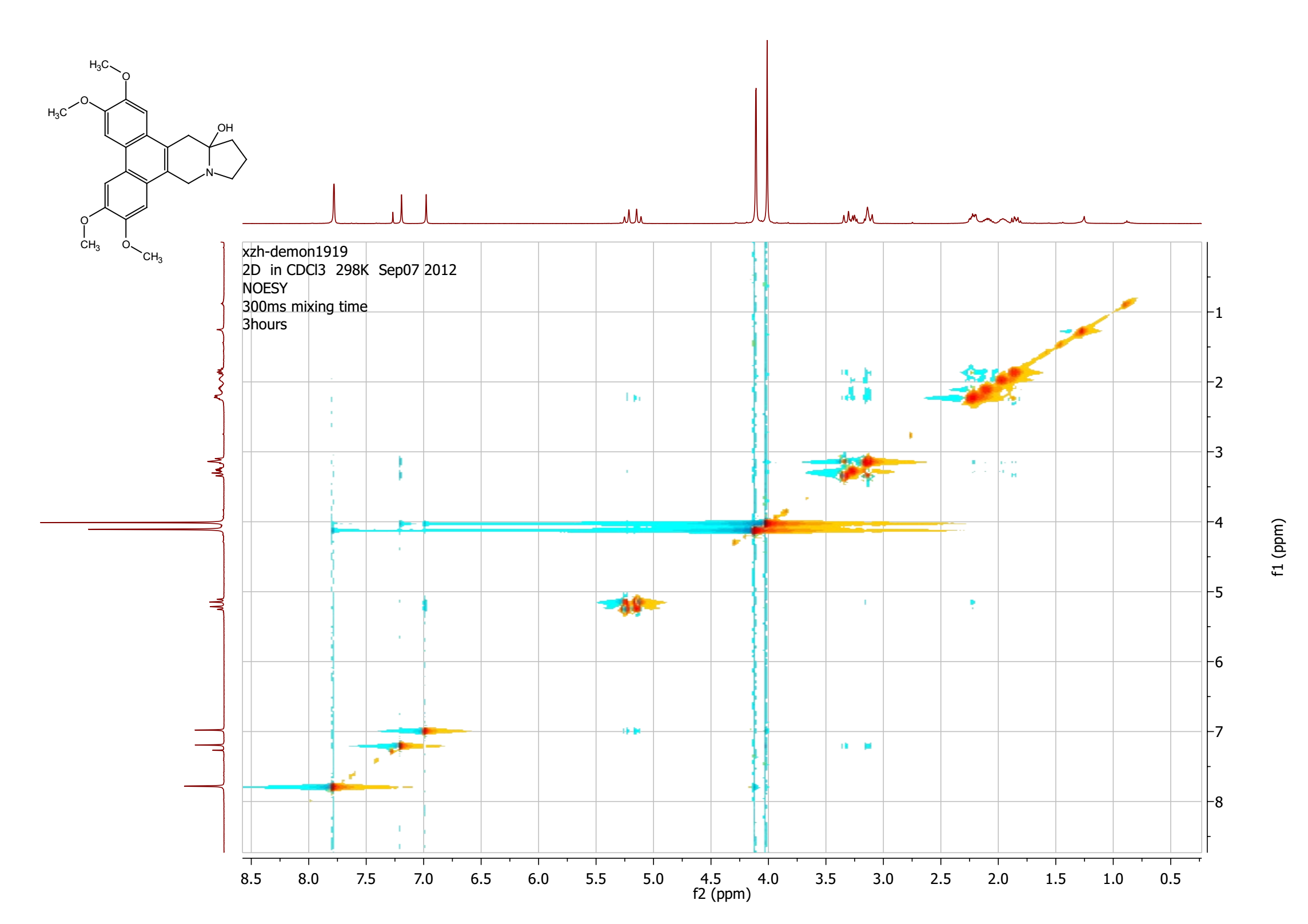


NOESY spectrum of **13a-hydroxytylophorine 1**


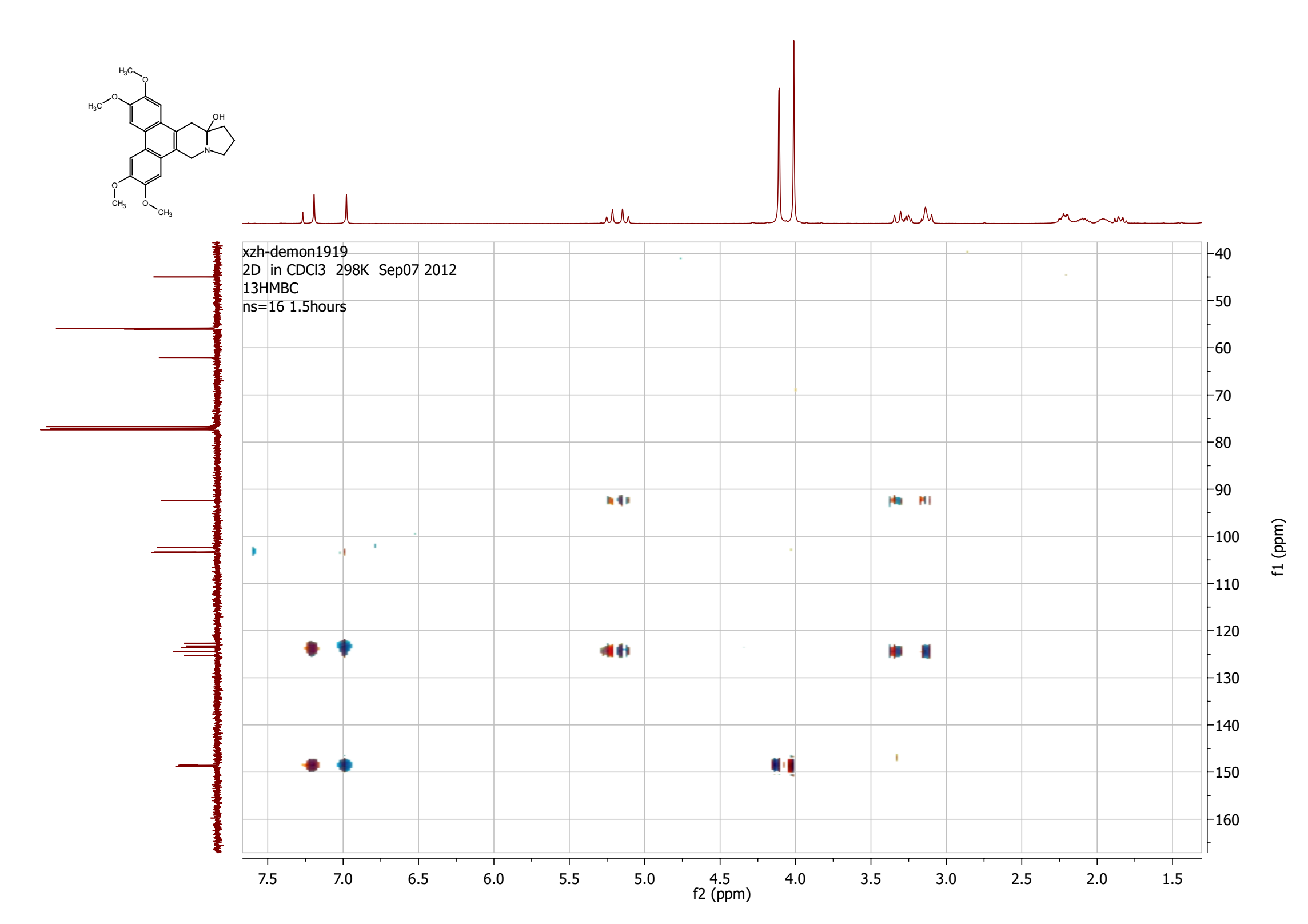


HMBC spectrum of **13a-hydroxytylophorine 1**


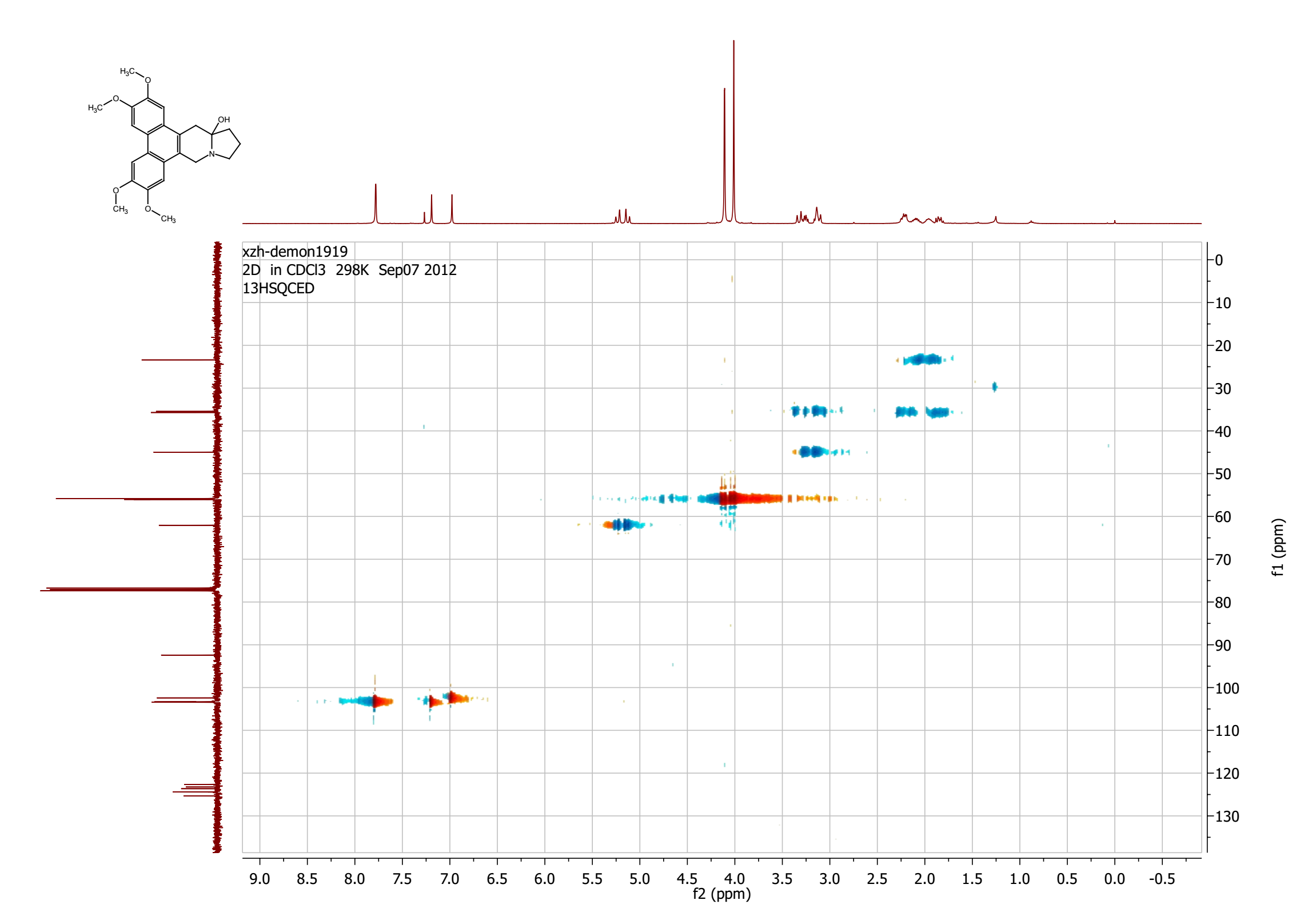


HSQC spectrum of **13a-hydroxytylophorine 1**

**Mechanism of one-pot reductive cyclization**

Figure S2. Proposed reaction pathways.

Base on the experiment results, we could rule out the pathway through nine-membered lactam. As shown in Figure S2, we proposed three possible reaction pathways. Amino lactone **14** could be reduced to active lactol **8** by NaBH_4_ in MeOH. In path a, lactol center of **8** was attacked by intramolecular amino group rapidly, then the hydroxyl group left to form hemiaminal **9**, which was further reduced to desired product **3**. There was another possibility: when the bridged oxygen left, **X1** was formed and rapidly dehydrated to Shiff base **X2**, which was reduced to **3**(path b). As the lactol had a weak tautomerization with aldehyde **X3**, intramoleculaly condensation would occur to form Shiff base **X2**, which was reduced to product **3**(path c).

We preferred path a was favorable, based on the following observations. First, we found the reaction was rather clean. In the progress of this reaction, there was only one major intermediate observed besides substrate **14** and product **3**. Then, we tried to separate this intermediate. Though pure sample was not obtained, we supposed its structure was **9** according to data of crude sample (Figure S3: signals at 6.27 ppm in the ^1^H NMR for the hemiaminal and 90.3, 66.0 ppm in DEPT-135 for two aliphatic CH fragments).


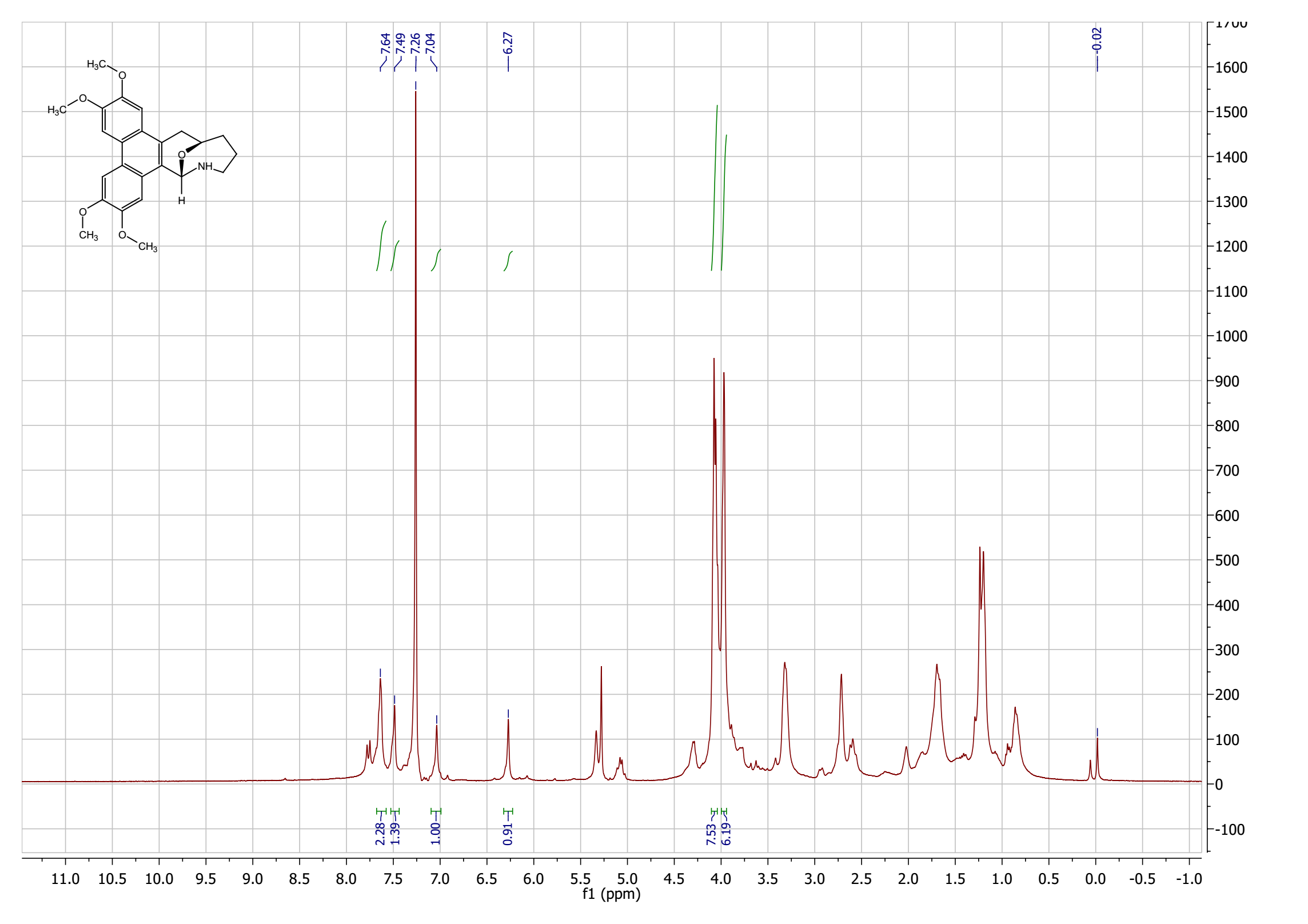

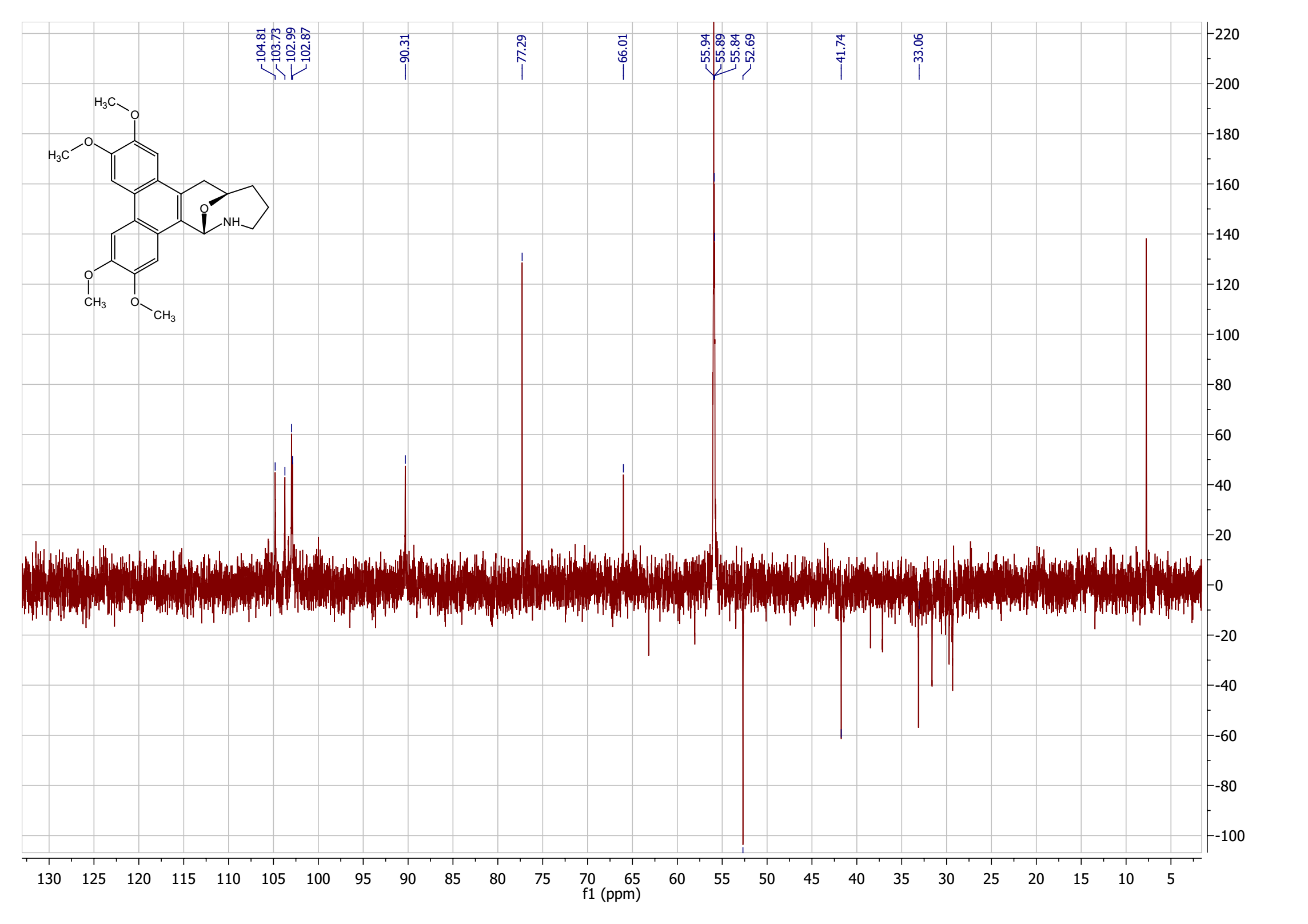


Figure S3. ^1^H NMR and DEPT-135 spectrum of possible **9**.

Second, we designed to detect the intermediate by HRMS to further understand this reaction.

Experiment 1: To a solution of 1 eq (1 mg) **14** in 0.1 mL MeOH was added 0.1 eq NaBH_4_ (<0.01 mg) at rt. After 1 min, a drop of reaction mixture was absorbed and sent to HRMS.

Major signal at 426.1913 was assigned to substrate **14** (calculated for [M+H^+^] 426.1911，found 426.1913.). Minor signal at 410.1955 might be assigned to **9** (calculated for [M+ H^+^] 410.1962, found 410.1955). Notably, **9** and **X2** were isomers (Figure S4).


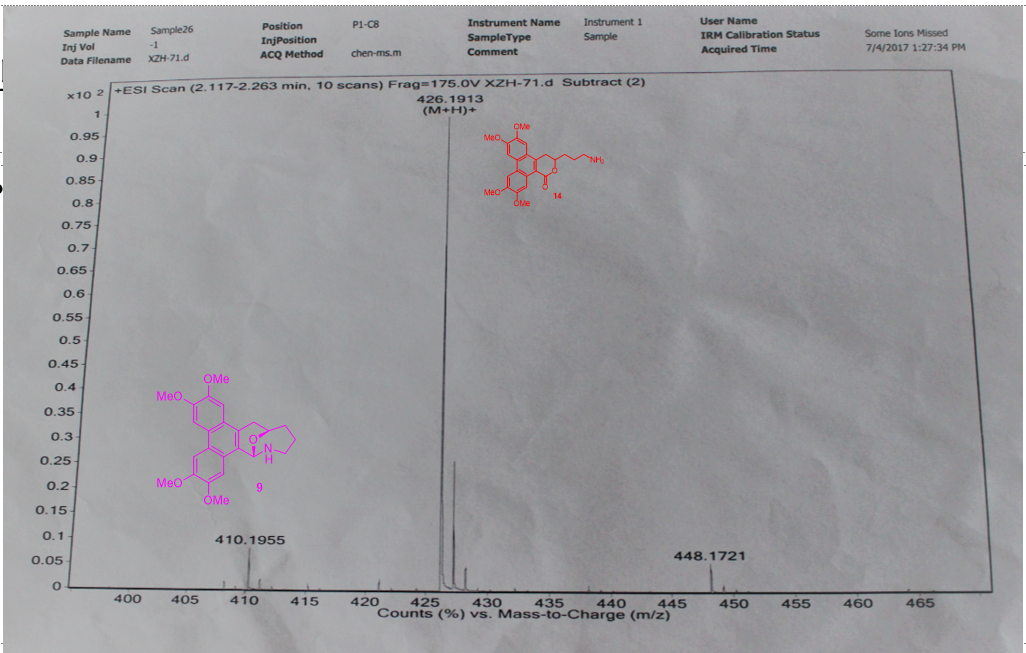


Figure S4. HRMS data for experiment 1.

Experiment 2: To a solution of 1 eq (1 mg) **14** in 0.1 mL MeOH. was added 5 eq NaBH_4_ (0.45 mg) at rt. After 20 mins, the substrate was consumed up; a drop of reaction mixture was absorbed and sent to HRMS.

Major signal at 412.2120 was assigned to product**3** (calcu for [M+ H^+^] 412.2118, found 412.2120.). And we also found the minor signal at 410.1961 for**9** (Figure S5).


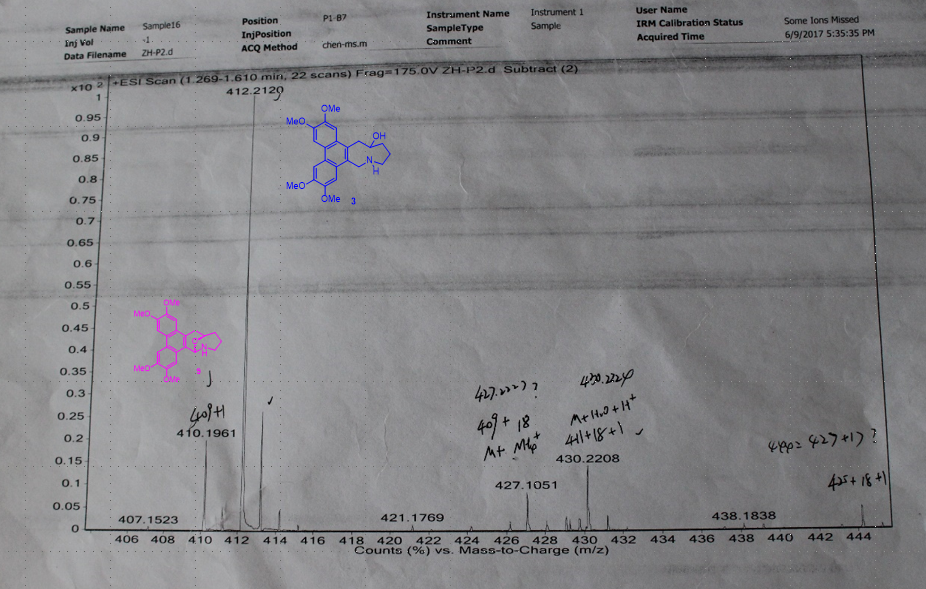


Figure S5. HRMS data for experiment 2.

To evaluate other pathways, the reaction solvent was removed when the reaction was conducting, and the residue was dissolved in *d*-CDCl_3_. The crude ^1^H NMR spectrumof the residue contained no signals for aldehyde X2 or Schiff base X3 (no signal above 9.0 ppm in Figure S6). Therefore, we thought path b and path c were not possible. Especially, direct condensation to form nine-membered Schiff base would suffer high transannular strain. In addition, the spectrum matched with the spectra of **9** (Figure S4), which indicated **9** was the most reasonable intermediate. As a result, the designed progress (path a) was favorable reaction pathway.


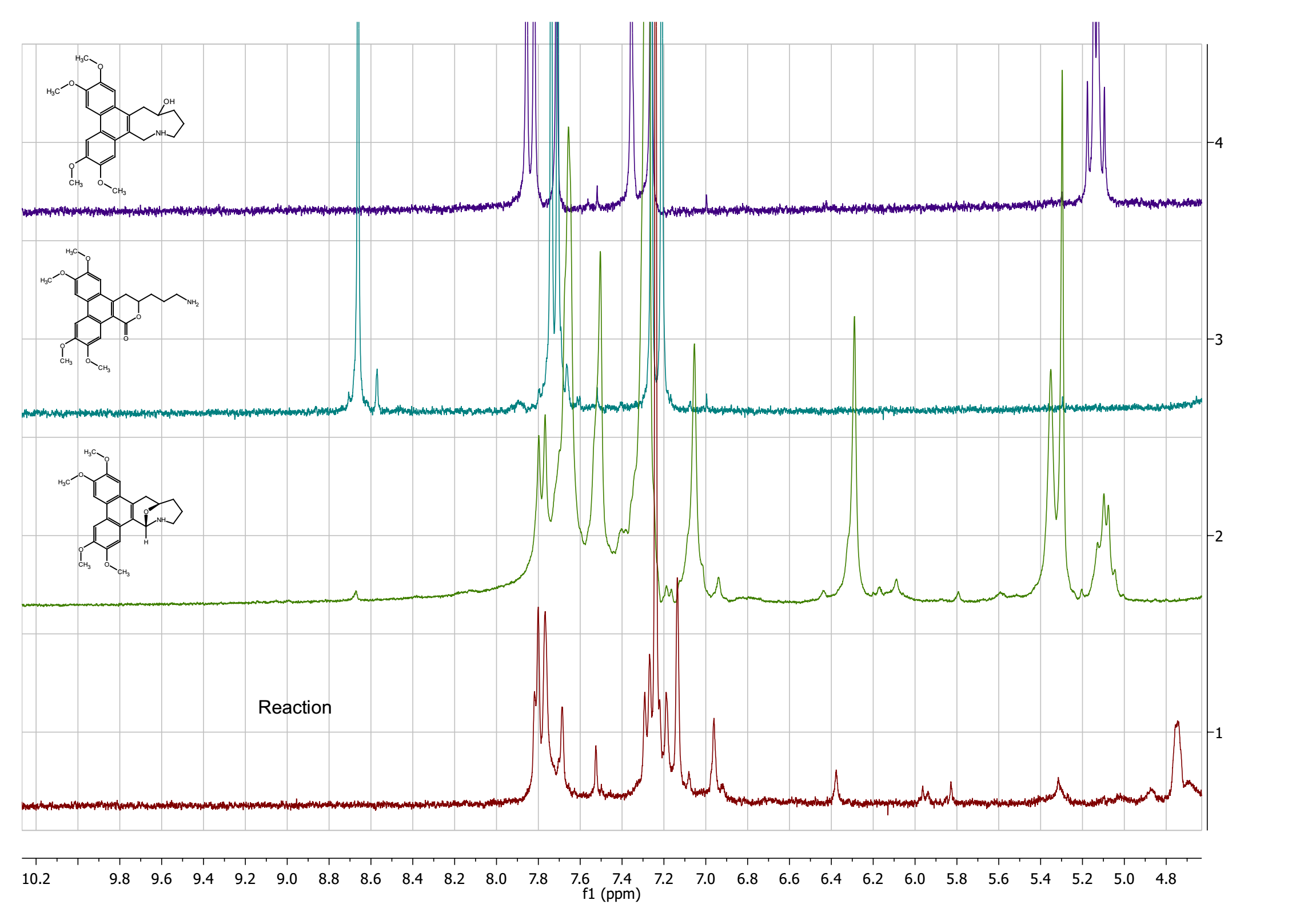


Figure S6. Comparison ^1^H NMR spectrums of **3**, **14**, **9** and reaction intermediate.

**Variation of ^1^H NMR Resonances of Synthetic 1**


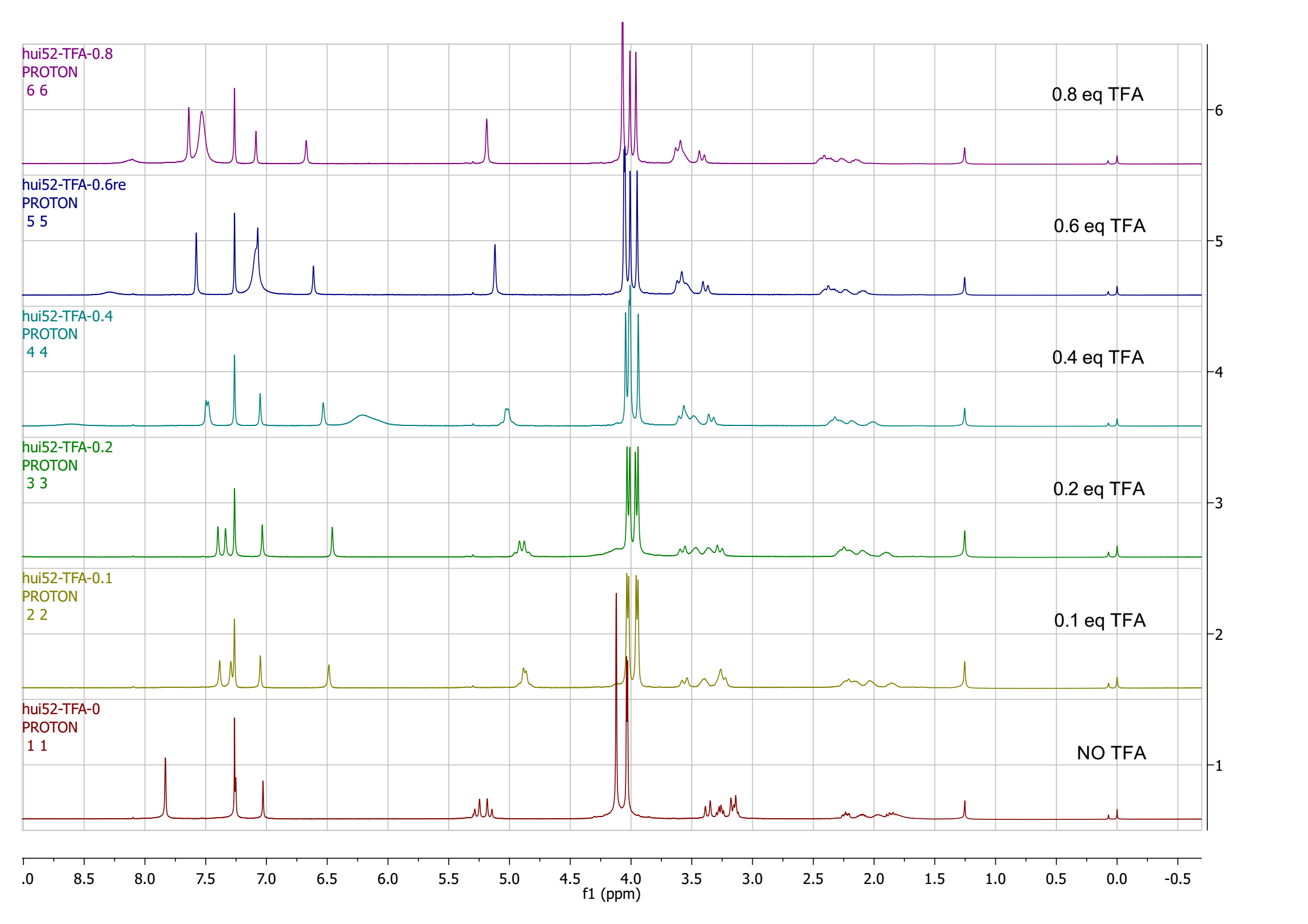


Figure S7. Variance of ^1^H NMR of aromatic protons with incremental amounts of TFA added. (CDCl_3_ was washed with K_2_CO_3_ to eliminate the influence of DCl before use. The sample could be recovered by dealt with K_2_CO_3_.)

Copies of ^1^H NMR spectrum for titration experiments


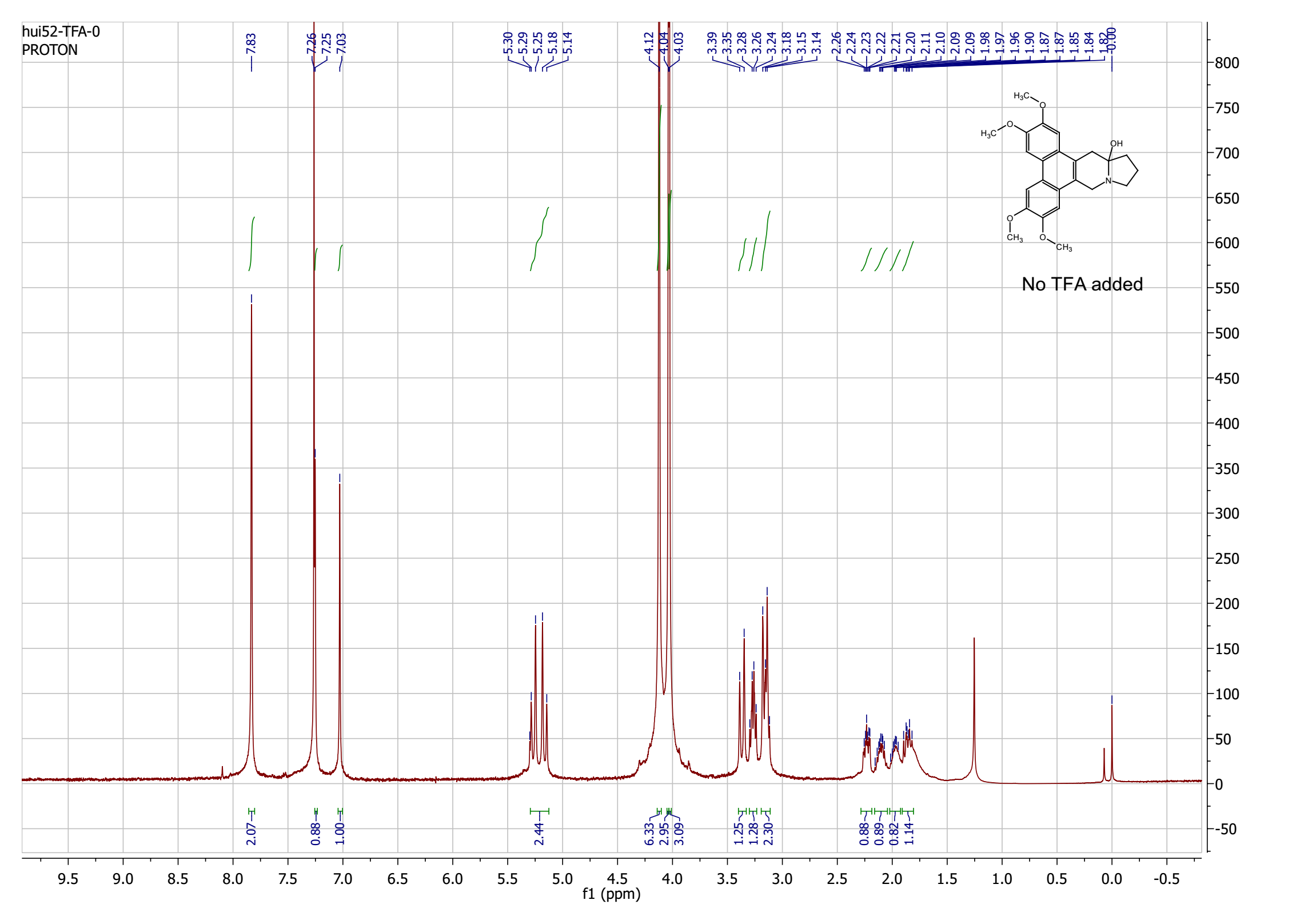


^1^H NMR spectrum of **1** with no TFA added


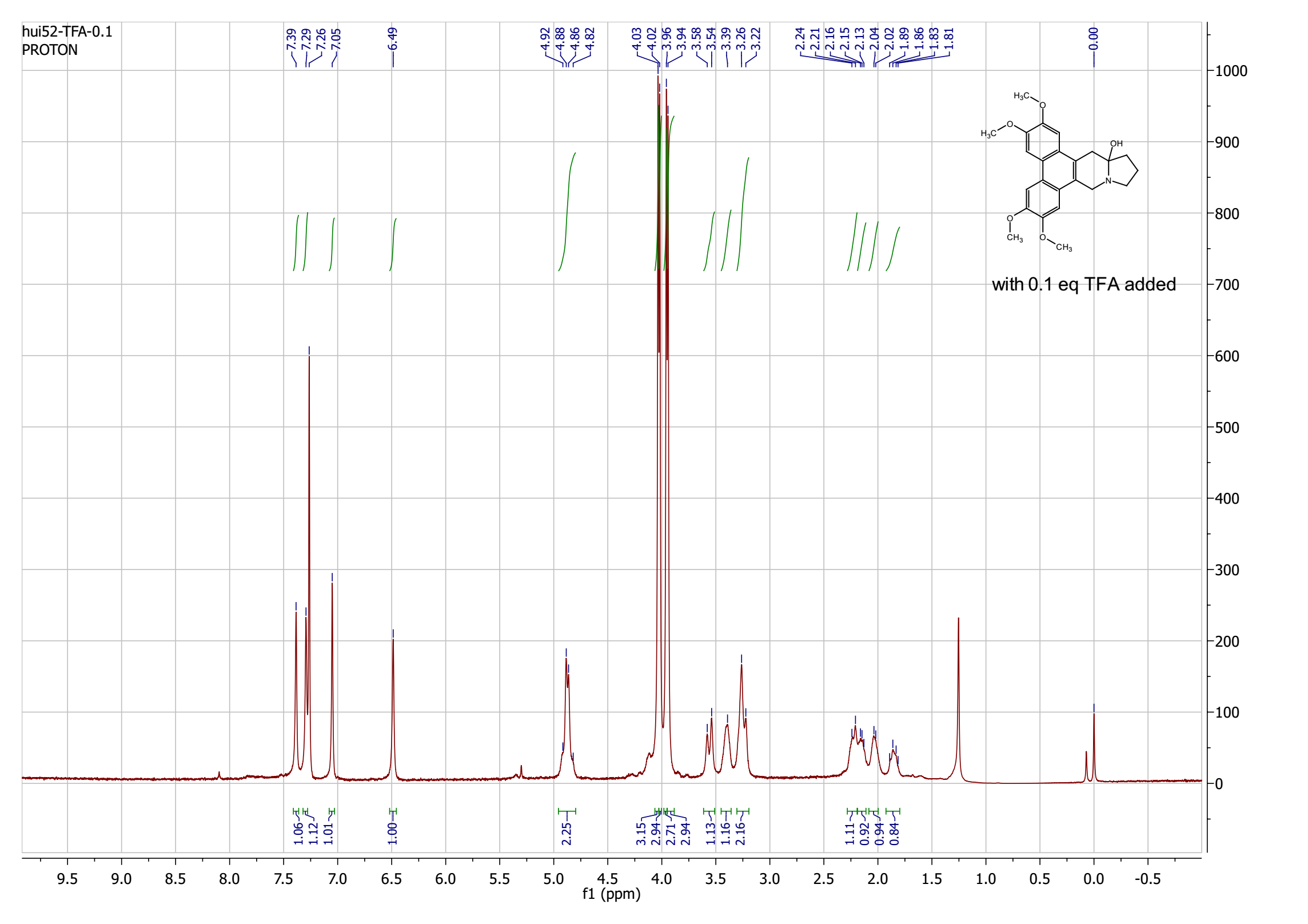


^1^H NMR spectrum of **1** with 0.1 eq TFA added


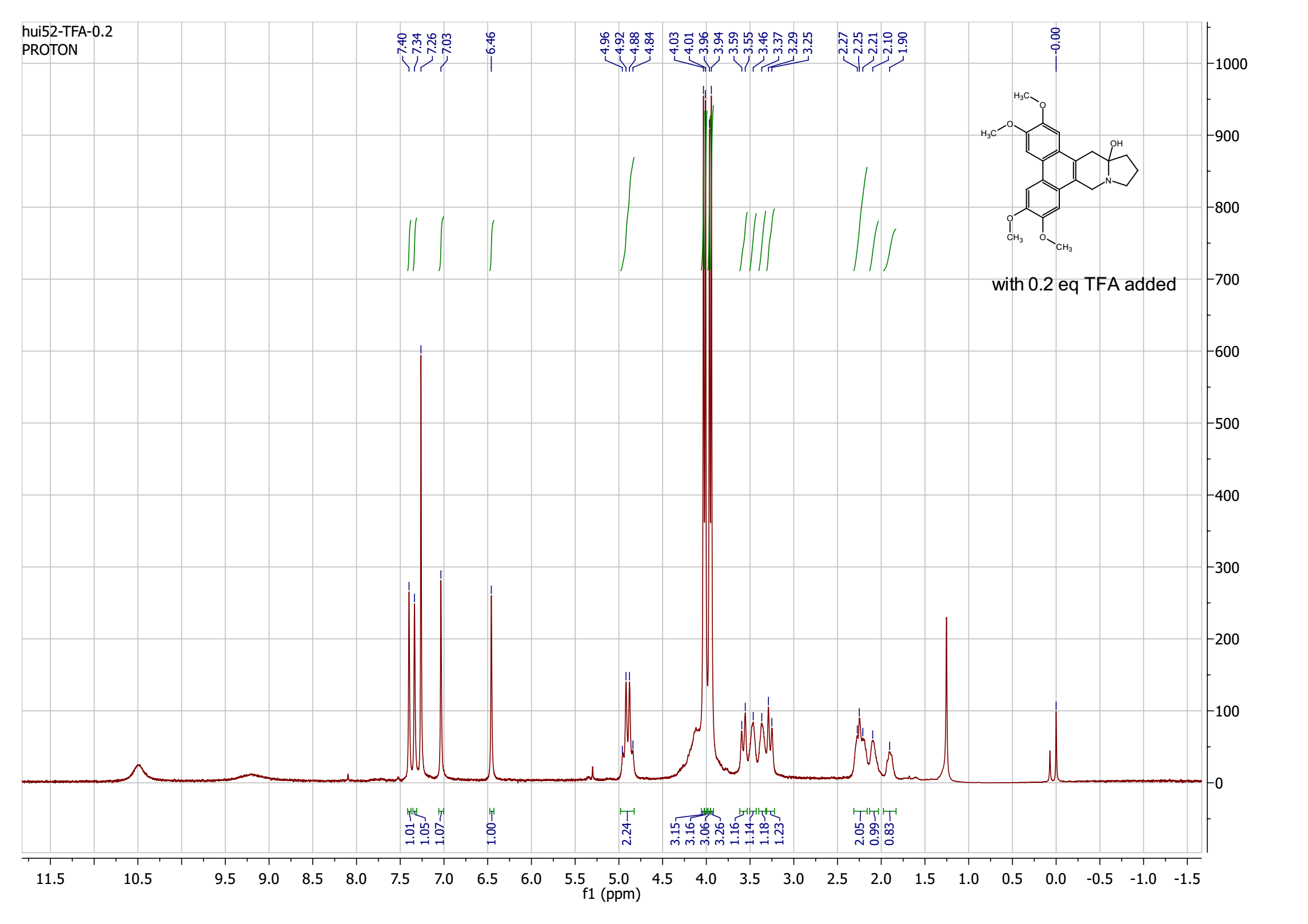


^1^H NMR spectrum of **1** with 0.2 eq TFA added


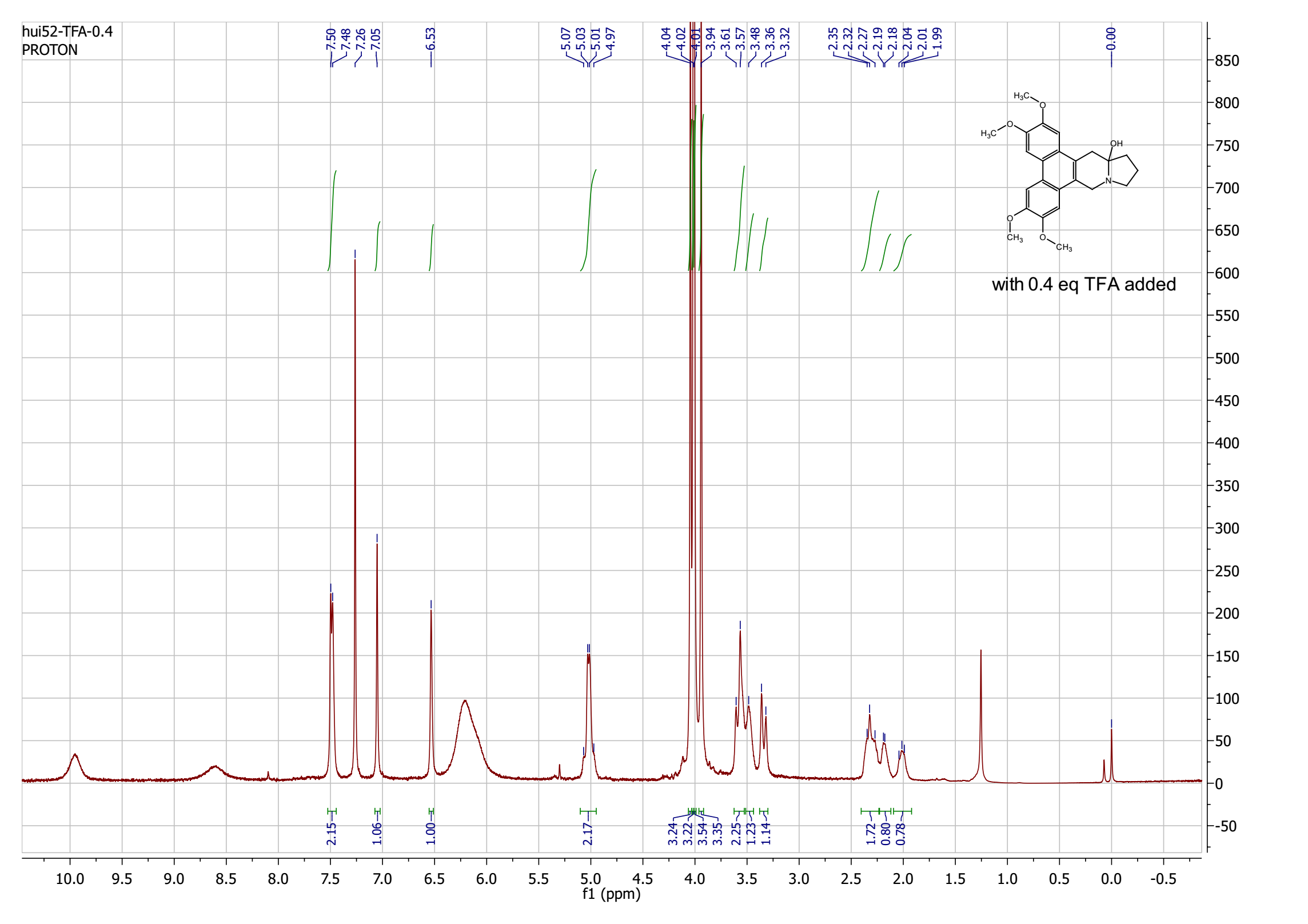


^1^H NMR spectrum of **1** with 0.4 eq TFA added


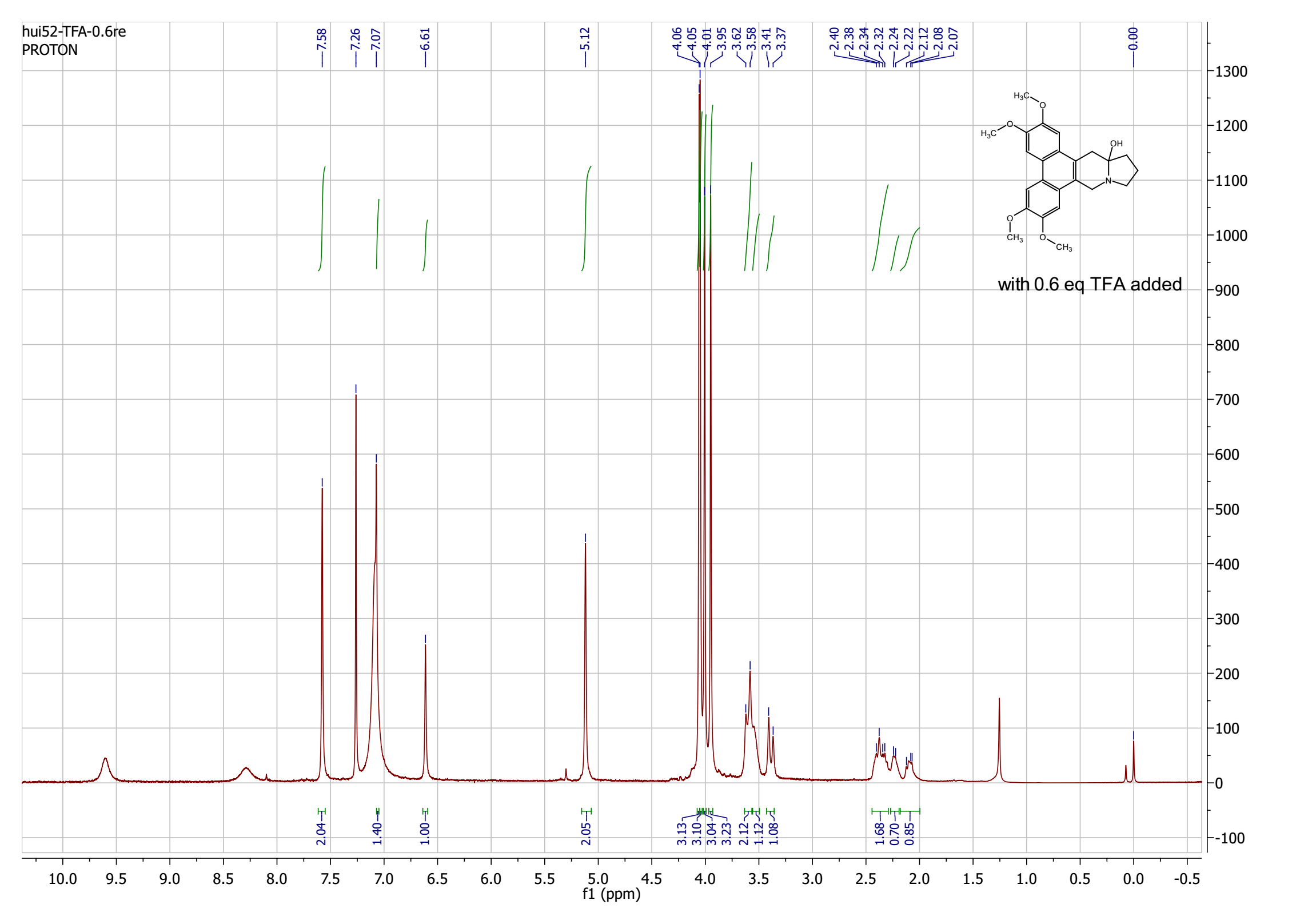


^1^H NMR spectrum of **1** with 0.6 eq TFA added


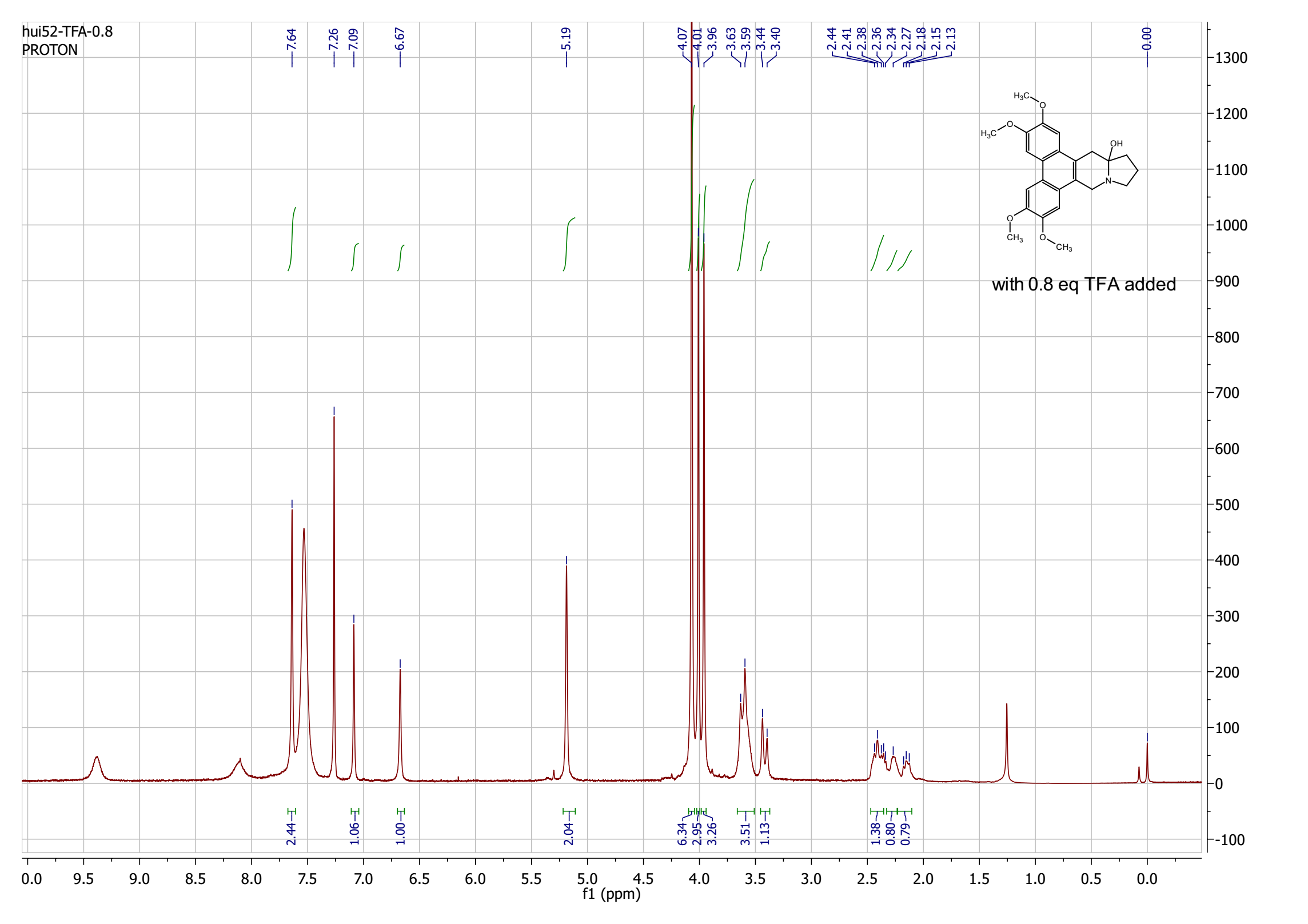


^1^H NMR spectrum of **1** with 0.8 eq TFA added

**Reference**

1. (a) Bates, R. W. & Dewey,M. R. A Formal Synthesis of Swainsonine by Gold-Catalyzed Allene Cyclization.*Org. Lett.***11**, 3706-3708 (2009);(b) Ott, M. M. & Little, D. Diyl Trapping Reactions to Synthesize Taxol Analogs. *J. Org. Chem.***62**, 1610-1616 (1997).
2. Bhutani, K. K., Ali. M. & Atal. C. K.13a-hydroxytylophorine from *Tylophora Hirsuta.Phytochemistry*. **24,** 2778-2780 (1985).
